# Analysis of the tonsillar microbiome in young adults with sore throat reveals a high relative abundance of *Fusobacterium necrophorum* with low diversity

**DOI:** 10.1371/journal.pone.0189423

**Published:** 2018-01-19

**Authors:** T. Prescott Atkinson, Robert M. Centor, Li Xiao, Fuchenchu Wang, Xiangqin Cui, William Van Der Pol, Casey D. Morrow, Amy E. Ratliff, Donna M. Crabb, Arthur H. Totten, Carlos A. Estrada, Michael B. Faircloth, Ken B. Waites

**Affiliations:** 1 Department of Pediatrics, School of Medicine, University of Alabama at Birmingham, Birmingham, Alabama, United States of America; 2 Department of Medicine, School of Medicine, University of Alabama at Birmingham, Birmingham, Alabama, United States of America; 3 Department of Biostatistics, School of Public Health, University of Alabama at Birmingham, Birmingham, Alabama, United States of America; 4 Center For Clinical & Translational Science, School of Medicine, University of Alabama at Birmingham, Birmingham, Alabama, United States of America; 5 Department of Cell, Developmental, & Integrative Biology, School of Medicine, University of Alabama at Birmingham, Birmingham, Alabama, United States of America; 6 Department of Pathology, School of Medicine, University of Alabama at Birmingham, Birmingham, Alabama, United States of America; 7 Department of Microbiology, School of Medicine, University of Alabama at Birmingham, Birmingham, Alabama, United States of America; 8 Department of Family & Community Medicine, School of Medicine, University of Alabama at Birmingham, Birmingham, Alabama, United States of America; University of Illinois at Urbana-Champaign, UNITED STATES

## Abstract

*Fusobacterium necrophorum* (*Fn*), a gram-negative anaerobe, is increasingly implicated as an etiologic agent in older adolescents and young adults with sore throat. Inadequately treated *Fn* pharyngitis may result in suppurative complications such as peritonsillar abscess and Lemierre’s syndrome. Data from the literature suggest that the incidence of life-threating complications in these age groups from *Fn* pharyngitis (Lemierre’s syndrome) in the United States exceeds those associated with group A beta-hemolytic streptococcal (GAS) pharyngitis (acute rheumatic fever). Using real-time PCR, we previously reported about a 10% prevalence of *Fn* in asymptomatic medical students and about 20% in students complaining of sore throat at a university student health clinic (p = 0.009). In this study, a comprehensive microbiome analysis of the same study samples confirms that *Fn* pharyngitis was more common than GAS pharyngitis. Eighteen patients were found to have *Fn* OTU values exceeding an arbitrary cutoff value of 0.1, i.e. greater than 10% of total sequences, with five subjects reaching values above 0.7. By contrast only 9 patients had GAS OTU values greater than 0.1 and none exceeded 0.6. When the data were analyzed using five separate assessments of alpha diversity, in each case for *Fn* there were statistically significant differences between *Fn* positive_high (OTU abundance > 0.1) vs control, *Fn* positive_high vs *Fn* negative (OTU abundance = 0), *Fn* positive_high vs *Fn* positive_low (OTU abundance > 0 and < 0.1). When the data were analyzed using three beta diversity indexes (Bray-Curtis, weighted unifrac, and unweighted unifrac), there were statistically significant differences between *Fn* positive_high (OTU abundance ≥ 0.1) vs control for all three. Statistically significant differences remained if we chose somewhat different OTU abundance cutoffs of 0.05 or 0.15. We conclude that Fn appears to play a dominant role in bacterial pharyngitis in the older adolescent and young adult age groups and that the development of a productive mucosal infection with *Fn* is linked to a significant decrease in the diversity of the associated tonsillar microbiome.

## Introduction

Several recent studies have provided strong evidence that *Fusobacterium necrophorum (Fn)* has a causative role in nonstreptococcal tonsillitis, particularly in adolescents and young adults and may occur at rates comparable to group A beta-hemolytic streptococcal (GAS) infection, sometimes exceeding 20% of cases.[1–6] *Fn* also causes up to 20% of recurrent, persistent sore throats.[7] Most epidemiologic studies looking at *Fn* prevalence in sore throats come from Europe. Outside of our recently published study, we are aware of only one other U.S. study, that of Van *et al*, which was recently conducted in children.[8] In this study of 300 children presenting with pharyngitis the prevalence of *Fn*, as determined by PCR and culture, was significantly higher in older adolescents (14–20 yo) (13.5%) than in children less than 14 yrs (1.9%) (p < 0.001). The clinical signs and symptoms of *Fn* positive subjects were similar to those with GAS. In an English study, Batty and Wren cultured 248 throat swabs and found that *Fn* was more commonly isolated than GAS from patients over 20 years of age.[4] Using real-time PCR to study tonsillitis patients aged 18 to 32 years and healthy controls in Denmark, Jensen and colleagues found that 48% of 105 nonstreptococcal tonsillitis patients were positive for *Fn* compared to 21% of controls, suggesting that this organism may be a major cause of bacterial pharyngitis.[3] In our published study of 312 sore throat patients and 180 controls in a U.S. university setting, Quantitative PCR (qPCR) identified *Fn* in 20.5% of patients and 9.4% of controls with GAS 10.3% and 1.1% respectively.[9] Finally, in a recent meta-analysis of 6 studies of acute tonsillitis, Klug and colleagues found that *Fn* was recovered significantly more frequently from patients (21.2%) compared to controls (7.6%) (p < 0.001).[6] Together these studies provide strong evidence that *Fn* is present in a substantial proportion of adolescents and young adults with bacterial pharyngitis.

*Fn* is established as a major cause of peritonsillar abscess.[10, 11] *Fn* pharyngitis or peritonsillar abscess may develop into a severe, life-threatening condition known as Lemierre’s syndrome in which fusobacterium invades the internal jugular vein causing a septic thrombophlebitis and then begins showering the body with septic emboli.[12, 13] Although it remains an uncommon complication, the incidence of Lemierre’s syndrome appears to have been increasing over the past two decades for unclear reasons and now is more common in the United States than acute rheumatic fever, the most serious complication of GAS pharyngitis.[1, 14] The incidence is increased among males, ranging from 1.5:1 to 3:1 in a few small published patient series, and the large majority of affected individuals are older adolescents or young adults (18–29 years).

Most primary care offices can perform quick antigen testing of throat swabs and overnight cultures for GAS. By contrast, no current method for diagnosing *Fn* pharyngitis exists for office practices despite the epidemiologic evidence that *Fn* causes at least as many cases of acute and recurrent bacterial pharyngitis in older adolescents and adults. Our recently published study provided the first data on the clinical characteristics of *Fn* pharyngitis although a second recently has been published. [6, 9] The Centor score, a clinical scoring system in which patients are awarded one point each for absence of cough, presence of tonsillar exudates, history of fever, and the presence of tender anterior cervical adenopathy, correlated with the presence of not only GAS but also with streptococcal groups C/G and *Fn* suggesting that this clinical scoring system is a quantitative summation of clinical signs and symptoms that develop in response to any invasive bacterial pharyngitis, not just GAS.[15] The Centor scoring system assigns one point each for a sore throat patient with fever, tonsillar exudates, tender adenopathy, and absence of cough, with higher scores having increasing probability of GAS pharyngitis. In response to our study, other investigators argue that, like GAS, *Fn* can be considered part of the normal flora and the simple presence of the organism should not be taken to represent infection.[16] We hypothesized that a bacterial tonsillitis could create a dysbiosis in the tonsillar microbial flora, although it might also be the case that an evolving dysbiosis associated with increasing predominance of *Fn* could play a role in the development of an inflammatory response. Therefore we carried out a comprehensive microbiome analysis to determine the relative proportion of *Fn* in comparison to other members of the oropharyngeal flora and to characterize potential differences in the microbial flora with regard to number and diversity in pharyngitis patients with high relative levels of *Fn* and those with low levels of *Fn* or no detectable *Fn* compared to asymptomatic controls.

## Materials and methods

### Patient and control samples

This work was conducted under a protocol approved by the UAB Institutional Review Board. We have previously described the procedures for enrollment in our IRB approved study and the patient and control demographics.[9] Briefly, the study subjects were between 15–30 years of age and included 341 university students presenting to a university student health clinic complaining of a sore throat, while the controls were 180 asymptomatic students. Potential study patients were not required to sign an informed consent by IRB since the procedure was innocuous and samples were anonymized. Patients received an information sheet that clearly informed them that they had the option whether or not to participate. The sore throat patients were enrolled sequentially in the student health clinic between March 2013 and March 2014 while the controls were enrolled mainly over four days in April 2013. Patients and controls were excluded if they were on antibiotics or had been on antibiotics during the previous four weeks. In our previous published study of these subjects, we analyzed data from only 312 of the 341 patients. Some were excluded because their ages fell outside the main range of interest (15–30 years), some because of incomplete demographics, and some because of incomplete PCR results. In this previously published study, DNA was extracted from throat swabs and analyzed by real-time PCR (RT-PCR) for the presence of β hemolytic streptococci, *Fusobacterium necrophorum* (*Fn*), and *Mycoplasma pneumoniae*. The latter organism was included because of previous reports suggesting that *M*. *pneumoniae* infection can be a cause of pharyngitis.[17] It is these previously acquired DNA samples that were used for microbiome analysis in the current study.

### Microbiome analysis using 16S/Next Generation Sequencing (NGS)

Using the DNA samples from all 341 patients and 30 controls, PCR was used with unique bar coded primers to amplify the variable region 4 (V4) region of the 16S rRNA gene to create a 16S V4 amplicon library from individual samples.[18–20] Only 30 of the 180 controls were used because it was felt that this subset of the normal controls would provide a representative sample of the healthy age-matched control oral microbiome for use as a comparator group. The PCR product was ~255 bases from the V4 segment of the 16S rRNA gene, and we sequenced 251 base single end reads using Illumina MiSeq. [18, 19] FASTQ conversion of the raw data files was performed following de-multiplexing using MiSeq reporter. Quality assessment of the FASTQ files was performed using FASTQC (http://www.bioinformatics.babraham.ac.uk/projects/fastqc/), and then quality filtering was done using the FASTX toolkit. Due to low quality of a single base toward the 3’ ends of the read, the last base was trimmed for all reads, making the read length 250 bases. Any read with an average base quality Q score of < 20 and the reads with unknown bases (“N”) were discarded. The remainder of the steps was performed with the Quantitative Insight into Microbial Ecology (QIIME) suite, version 1.8 as described below. [18, 21, 22] Chimeric sequences were filtered using the “identify_chimeric_seqs.py” module of USEARCH. [23] Sequences were grouped into operational taxonomic units (OTUs) using the clustering program UCLUST at a similarity threshold of 97%. [23] The Ribosomal Database Program (RDP) classifier trained using the Greengenes (v13.8) 16S rRNA database [24] was used to make taxonomic assignments for all OTUs at confidence threshold of 80% (0.8). [25] The resulting OTU table included all OTUs, their taxonomic identification, and abundance information. OTUs whose average abundance was less than 0.005% were filtered out. OTUs were then grouped together to summarize taxon abundance at different hierarchical levels of classification (e.g. phylum, class, order, family, genus, and species). These taxonomy tables were also used to generate stacked column bar charts of taxon abundance using Microsoft Excel software (Microsoft, Seattle, WA). Multiple sequence alignment of OTUs was performed with PyNAST. [26]

### Statistical analyses

Equal variances among groups were assessed by Levene’s Test. The Welch’s ANOVA was used for testing alpha diversities, Shannon and Simpson, that had unequal variances. For pair-wise comparisons among groups, Tukey’s HSD post hoc t-test was used to test for alpha diversities with homogeneous variance, Chao1, observed species, and PD Whole Tree. Bonferroni correction was used for alpha diversities with unequal variances, Shannon and Simpson. PERMANOVA test [27] was performed to test the statistical significance between the four groups for three beta diversity indices (Bray Curtis, weighted unifrac and unweighted unifrac).[28] Student’s t test was used to compare the Centor scores for different patient groups. Significance level of 0.05 was used for all statistical tests. All the analyses were conducted using R (Version 3.3.2) except for the correlation of qPCR Ct values with OTU values, which was performed using GraphPad Prism, and t tests, which were performed in Excel spreadsheets.

## Results

### Predominance of *Fn* in the tonsillar microbiome in young adults with sore throat

**Fig 1:**
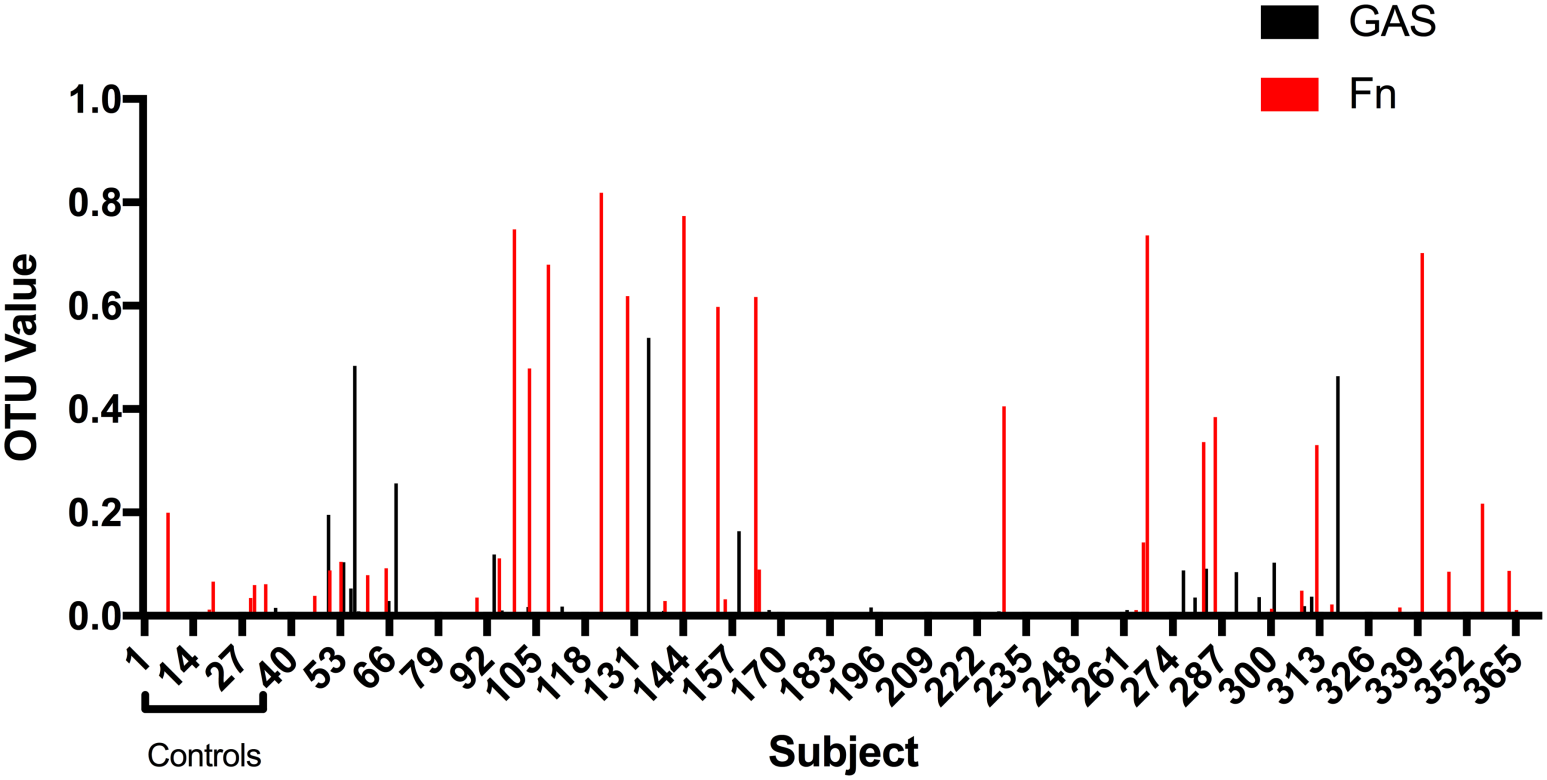
Microbiome analysis of throat swabs from 367 subjects (30 controls and 337 sore throat patients). Shown are the relative proportions of OTUs with higher values represented by *Fusobacterium necrophorum* and *Streptococcus pyogenes*. Many positive samples (OTU values less than 0.01) cannot be seen on this scale.

Of the 341 patient samples and 30 control samples submitted for microbiome analysis, four sore throat patient samples did not yield interpretable results leaving 337 patient samples and 30 control samples for a total of 367. An OTU corresponding to *Fn* was detected in 217 patients (64.4%) and 23 controls (76.7%) (Table 1) (Fig 1) suggesting *Fn* is both a part of the normal pharyngeal tonsillar flora and present in infection. We reasoned that higher OTU values were more likely to represent true infection and arbitrarily selected a cutoff value of 0.1 for patients with possible infection due to *Fn*. In 18 sore throat patients (5.3%) the OTU value for *Fn* was greater than or equal to 0.1; however, one of 30 controls also had an OTU value in this range (3.3%).

**Table 1:** Comparison of findings in original PCR-based sample testing and current microbiome-based analyses.

	RT-PCR		Microbiome	
	Patients (312)	Controls (180)	Patients (337)	Controls (30)
***Fn***	**67 (21.5%)**	**18 (10%)**	**217 (64.45%)**	**23 (76.7%)**
**GAS**	**32 (10.3%)**	**2 (1.1%)**	**129 (38.3%)**	**9 (30%)**

**Fig 2:**
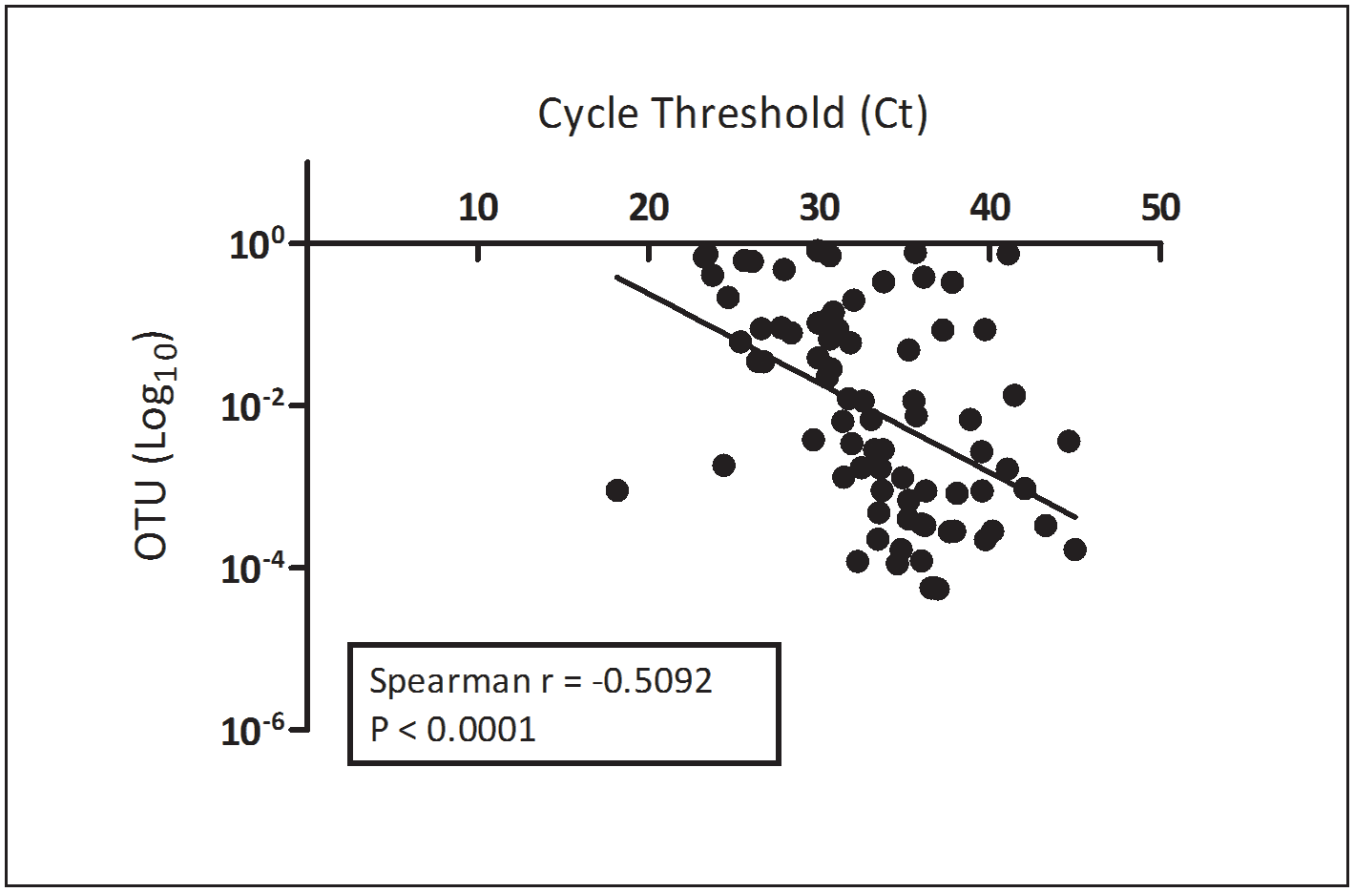
Correlation between Ct values for all *Fn* PCR positive samples and the corresponding OTU value for that sample.

In contrast, in our published report, using an extensively validated in-house qPCR assay, 85 samples were positive for *Fn* using the *Fn* rpoB gene as target: 67 patients (n = 312, 21.5%) and 18 controls (n = 180, 10%). Four controls and three sore throat patients who were positive by PCR were negative by 16S/NGS, and no samples were negative by PCR that were positive by 16S/NGS sequencing. Although the two techniques (qPCR and 16S/NGS sequencing) yielded very different results, there was a significant correlation between the cycle threshold (Ct) values obtained by qPCR (the threshold value for positivity, which correlates with the amount of template present) with the OTU value for *Fn* positive samples (Fig 2).

An OTU corresponding to *Streptococcus pyogenes* (GAS) was detected in 129 of 337 patient samples (38.3%) and 9 of 30 controls (30%). Of these, nine OTU values were greater than 0.1, and all were within the patient samples. Eight of these nine samples with high positive OTU values for *Streptococcus pyogenes* were also positive for *Fn* with low OTU values and the ninth sample was negative. Ten of 18 patient samples with high *Fn* OTU values were positive for GAS at low levels and the remaining samples were negative. Again, fewer patients (32, 10.3%) were found to have GAS in our published study with only 2 controls (1.1%) positive.

### Analysis of diversity in patient subgroups and control samples

We compared the diversity of bacteria in different patient sample sets and control subjects using all five alpha diversities (chao1, observed species, phylogenetic diversity—whole tree, Shannon and Simpson). In each case there exist statistically significant differences between *Fn* positive_high patients (OTU abundance > 0.1) and the other three groups: *Fn* positive_low patients (OTU abundance > 0 and < 0.1), *Fn* negative patients (OTU abundance = 0), and control subjects (Table 2 and Table A in S1 File). There were statistically significant differences among the four groups (positive_high and positive_low, negative, and control) in three beta diversities (Bray-Curtis, weighted unifrac, and unweighted unifrac) (Table I in S1 File) by overall PERMANOVA. We also did pair-wise PERMANOVA comparisons between every two groups. *Fn* positive_high and the control group have a statistically significant difference when the data are analyzed using the three beta diversity indexes for three different abundance cutoffs (*Fn*_high ≥0.1, *Fn*_high≥0.05 and *Fn*_high≥0.15). There exist statistically significant differences between control vs negative, control vs positive_high and control vs positive_low when we use Bray-Curtis and Unweighted unifrac as beta diversity methods for three different abundance cutoffs (*Fn*_high ≥0.1, *Fn*_high ≥0.05 and *Fn*_high ≥0.15). Thus, *Fn* positive samples with high OTU values suggestive of an infection have lower alpha and beta diversity.

**Table 2:** Comparison of alpha diversity by five measures in *Fn* and GAS positive and negative patient groups, and control subjects.

**Fn Using Positive High OTU Values > 0.1**									
	**Stratified by Group**								
	**Control**		**Negative**		**Positive_high**		**Positive_low**		
**n**	**30**		**120**		**18**		**199**		
	**Mean**	**SD**	**Mean**	**SD**	**Mean**	**SD**	**Mean**	**SD**	**p**
**chao1**	**260.24**	**41.29**	**253.18**	**36.27**	**209.38**	**53.68**	**257.08**	**42.47**	**<0.001**
**observed_species**	**210.6**	**30.04**	**205.1**	**32.28**	**165.72**	**46**	**210.69**	**34.92**	**<0.001**
**PD_whole_tree**	**14.18**	**1.43**	**13.33**	**1.56**	**12.41**	**2.12**	**13.76**	**1.87**	**0.001**
**shannon**	**4.52**	**0.49**	**4.34**	**0.48**	**3.06**	**1.08**	**4.39**	**0.55**	**<0.001**
**simpson**	**0.91**	**0.05**	**0.89**	**0.06**	**0.67**	**0.2**	**0.9**	**0.08**	**<0.001**
**GAS Using Positive_High OTU Values > 0.05**									
	**Stratified by Group**								
	**Control**		**Negative**		**Positive_high**		**Positive_low**		
**n**	**30**		**208**		**13**		**116**		
	**Mean**	**SD**	**Mean**	**SD**	**Mean**	**SD**	**Mean**	**SD**	**p**
**chao1**	**260.24**	**41.29**	**252.44**	**40.64**	**252.64**	**33.87**	**254.47**	**46.09**	**0.814**
**observed_species**	**210.6**	**30.04**	**205.72**	**34.32**	**201.54**	**33.34**	**207.88**	**39.26**	**0.821**
**PD_whole_tree**	**14.18**	**1.43**	**13.37**	**1.73**	**13.88**	**1.85**	**13.78**	**1.92**	**0.044**
**shannon**	**4.52**	**0.49**	**4.3**	**0.6**	**4.25**	**0.61**	**4.3**	**0.7**	**0.312**
**simpson**	**0.91**	**0.05**	**0.89**	**0.09**	**0.87**	**0.08**	**0.88**	**0.11**	**0.437**
**GAS using Positive_high OTU Values > 0.1**									
	**Stratified by Group**								
	**Control**		**Negative**		**Positive_high**		**Positive_low**		
**n**	**208**		**30**		**9**		**120**		
	**Mean**	**SD**	**Mean**	**SD**	**Mean**	**SD**	**Mean**	**SD**	**p**
**chao1**	**260.24**	**41.29**	**252.44**	**40.64**	**251.6**	**33.91**	**254.49**	**45.72**	**0.81**
**observed_species**	**210.6**	**30.04**	**205.72**	**34.32**	**196.67**	**31.5**	**208.03**	**39.11**	**0.705**
**PD_whole_tree**	**14.18**	**1.43**	**13.37**	**1.73**	**13.92**	**1.63**	**13.78**	**1.93**	**0.044**
**shannon**	**4.52**	**0.49**	**4.3**	**0.6**	**4.12**	**0.64**	**4.31**	**0.7**	**0.231**
**simpson**	**0.91**	**0.05**	**0.89**	**0.09**	**0.85**	**0.09**	**0.88**	**0.11**	**0.346**

In these three tables, *Fn* positive_high is the set of patient samples with *Fn* OTU values > 0.1 and *Fn* positive_low is the set with nonzero *Fn* OTU values ≤ 0.1. GAS positive_high is that set of patient samples > 0.05 (middle table) or > 0.1 (lower table) and GAS positive_low is the set with nonzero GAS values ≤ 0.05 (middle table) or ≤ 0.1 (lower table). The p values shown are the probabilities that the diversity in the set of *Fn* positive_high patients (upper table) or GAS positive_high (lower two tables) is different from the other two patient sets and control subjects.

Analysis of alpha diversity for GAS with high OTU values compared to other patient groups reveals only a marginally significant difference between any two groups with Phylogenetic Diversity–Whole Tree and no significant difference among the remaining four alpha diversities, no matter whether we choose OTU abundance > 0.05 or >0.1 as the threshold for subgroup "positive_high" (Table 3, Table K in S1 File and Table U in S1 File). When the data are analyzed using beta diversity indexes Bray-Curtis and Unweighted, for both cutoffs (GAS> 0.05 and >0.1), there is a statistically significant difference between the control vs GAS negative, control vs GAS positive_low and the control vs GAS positive_high. There exists a statistically significant difference between GAS positive_high and GAS positive_low if we conduct pair-wise comparison by using Weighted unifrac index. We feel it is likely that the discrepancy between the results from weighted and unweighted unifrac simply indicates that our grouping based on *Fn* or GAS relative abundance affects the survival (presence or absence) of different taxa but not the abundance, which is not a unique phenomenon in microbiome analyses.

**Table 3:** OTU values showing the relative proportion of total *Fn* or *S*. *pyogenes* sequences in which the OTU value in microbiome analysis exceeded 0.1 (i.e. more than 10% of total bacterial 16S sequences obtained) and the corresponding Centor scores among 312 sore throat patients.

*Fn* samples > 0.1			*Strep pyogenes* > 0.1			Neither *Strep pyogenes* nor *Fn* and Score = 4		
Study #	O TU	SCORE	Study #	O TU	SCORE	Study #	Largest O T U	Value
**4023**	**0.10445**	**2**	**4020**	**0.19552**	**2**	**4028**	***Veillonella dispar***	**0.306143**
**4066**	**0.11127**	**2**	**4024**	**0.10397**	**1**	**4063**	***Gemella sp***.	**0.34881**
**4070**	**0.7483**	**4**	**4027**	**0.48395**	**3**	**4141**	*Prevotella melanogenica	**0.23466**
**4074**	**0.47896**	**4**	**4038**	**0.25662**	**2**	**4148**	*Haemophilus parainfluenzae	**0.121262**
**4079**	**0.67939**	**4**	**4065**	**0.11886**	**4**	**4190**	***Fusobacterium periodonticum***	**0.193189**
**4093**	**0.81857**	**2**	**4107**	**0.5376**	**3**	**4196**	***Veillonella dispar***	**0.205305**
**4100**	**0.61893**	**2**	**4133**	**0.16335**	**2**	**4212**	***Prevotella melanogenica***	**0.192268**
**4118**	**0.77347**	**1**	**4278**	**0.10316**	**2**	**4237**	***Prevotella melanogenica***	**0.192879**
**4127**	**0.5981**	**4**	**4295**	**0.46406**	**1**	**4260**	*Prevotella melanogenica	**0.107159**
**4137**	**0.61707**	**2**				**4292**	***Neisseria sp***.	**0.279068**
**4204**	**0.40553**	**4**				**4316**	***Neisseria sp***.	**0.238371**
**4243**	**0.14194**	**2**						
**4244**	**0.73648**	**3**						
**4259**	**0.33666**	**2**						
**4262**	**0.38465**	**4**						
**4289**	**0.33073**	**4**						
**4319**	**0.70206**	**2**						
**4335**	**0.21701**	**2**						
	**Mean**	**2.78**		**Mean**	**2.22**			
	**SD**	**1.06**		**SD**	**0.97**			

*Other OTUs with similar values (polymicrobial?)

For comparison the most abundant OTUs in all other patients with Centor scores of 4 are shown.

**Fig 3:**
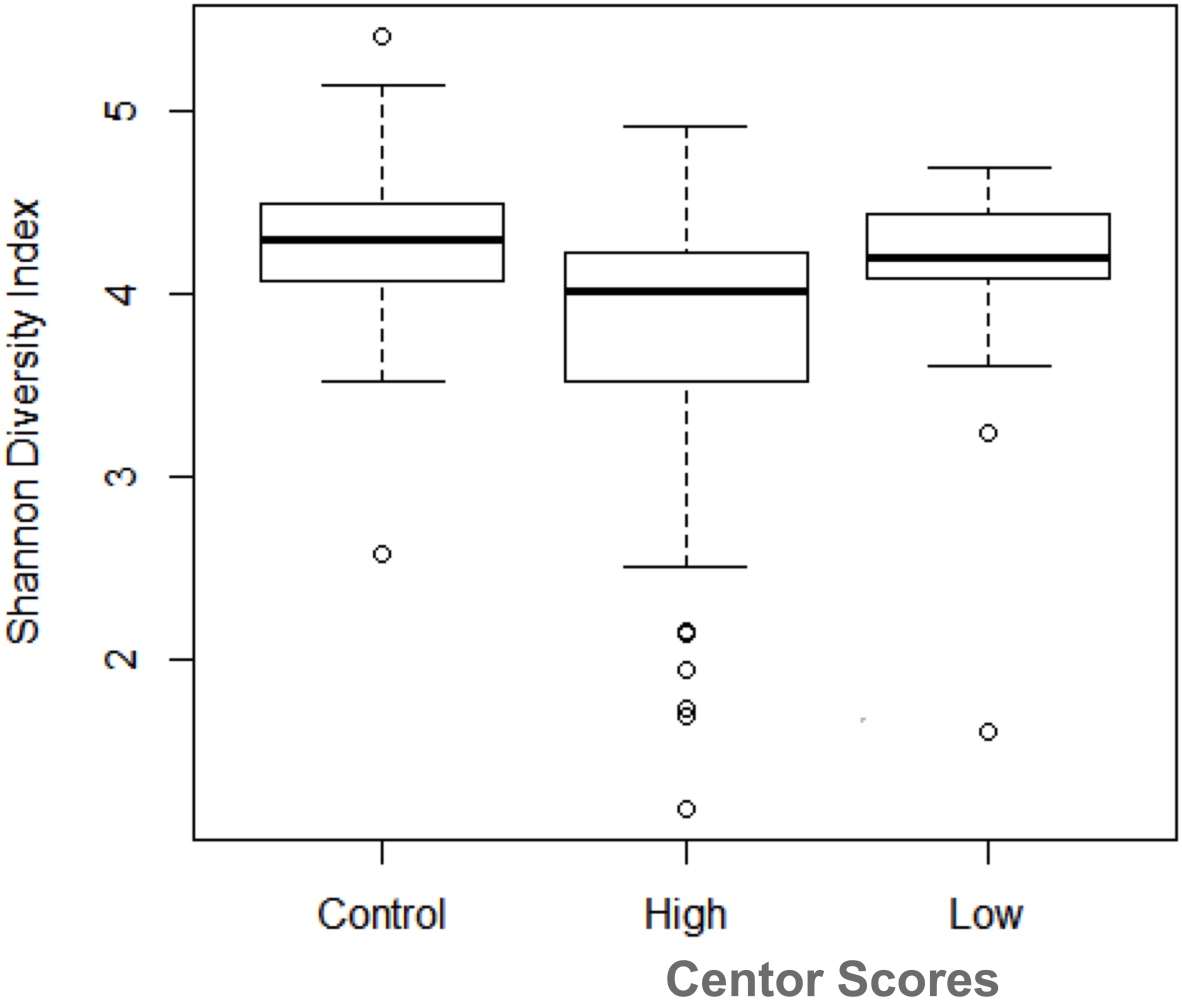
Lower diversity in *Fn* positive patient samples with higher Centor scores. *Fn* positive sore throat patient samples with higher Centor scores (≥ 2) are significantly less diverse than control samples but *Fn* positive patient samples with lower Centor scores (0 or 1) do not differ significantly from controls in diversity.

Samples with *Fn* OTU values > 0.1 (*Fn* positive_high) had similar Centor scores to the GAS positive samples with OTU values > 0.1 (2.78 ± 1.06 vs 2.22 ± 0.97, p = 0.2 (student’s t test)) (Table 3). Eleven other patients with Centor scores of 4 had OTU values for other taxa ranging from 0.107 to 0.349; these are listed in Table 3 for comparison. The relatively high prevalence of these other species in these sore throat patients support previous studies which have implicated species such as *Prevotella melanogenica* (along with *Fn*) in recurrent tonsillitis.[29] The *Fn* positive_high group had significantly higher scores than those for the entire sore throat patient cohort (1.64 ± 1.05, p = 0.0002), the *Fn* positive_low patients (nonzero OTU values ≤ 0.1) (1.58 ± 1.04, p = 0.0002), or the *Fn* negative patients (1.61 ± 0.99, p = 0.0004). These differences remained strongly significant if we chose somewhat different arbitrary OTU cutoff values for *Fn* high positive samples of 0.05 or 1.15. Finally, within the *Fn* positive group, those samples with Centor scores ≥ 2 were significantly less diverse than the control samples (Fig 3) (p = 0.00375) while those *Fn* positive samples with lower Centor scores did not differ from the controls in Shannon diversity.

## Discussion

Lemierre’s syndrome, a dangerous complication of *Fn* pharyngitis, now appears to be more frequent among adolescents and young adults in North America and Europe than acute rheumatic fever. It has long been appreciated that invasive *Fn* infections are much more common in the older adolescent/young adult age group. The reasons for the relatively high prevalence in this age range is unclear but it is interesting that one group found in a study of 411 university students that those who were *Fn* positive were more likely to report lip-to-lip kissing within the previous 4 weeks.[30] Our previously published report, in which 20.5% of sore throat patients’ throat swabs were positive for *Fn* compared to 10.3% positive for GAS (with 9.4% and 1.1% respectively positive in the controls), now augmented by the data presented in this manuscript, suggests that *Fn* pharyngitis may be more common among college students than GAS pharyngitis or pharyngitis due to other types of streptococci. High levels of *Fn* in the oropharynx of sore throat patients were not only more frequent than GAS, but the proportion of microbial sequences made up by the *Fn* OTU strongly suggests that this organism may be actually causing an infection in this subset of *Fn* positive patients as opposed to representing an incidental detection. Furthermore, the correlation of the Centor scores in the *Fn* high subset of patients also indicates that, as with GAS, the Centor scoring system is identifying patients with a bacterial pharyngitis. Together our data comprise strong evidence adding to a growing body of data that *Fn* is a common and underappreciated pathogen specifically affecting older adolescents and young adults. Unfortunately, at present there are no ready ways for primary care physicians to test for this common infection and little is known regarding how it spreads through the population or its patterns of antibiotic resistance.

Perhaps unsurprisingly, bacterial diversity is decreased in subjects with high levels of *Fn* or GAS. As the proportion of a species increases within a sample, the proportions of most other species should lead to a decrease in the overall diversity. The emerging dominance of a single species could result in conditions (e.g. changes in local pH, unavailability of necessary nutrients, presence of toxins) that favor the lower diversity. In the five *Fn* samples with the highest OTU values, over 70% of sequences were made up of that OTU alone. Those *Fn* positive samples with higher OTU values or higher Centor scores were associated with lower diversity. The data suggest that as an infection develops in the tonsillar microbial community, one pathogenic species becomes increasingly prevalent and concomitantly the overall diversity of the population declines. The reasons for this decline in diversity are likely to be numerous, ranging from an apparent decline in numbers of other species simply because of overwhelming predominance of a single species to inhibition of growth of other species by depletion of vital nutrients or the creation of an actively hostile environment. Regardless, similar changes have been documented in a wide range of infections and hosts ranging from pulmonary tuberculosis in humans [31], to *Cryptosporidium* intestinal infection in lower primates [32], to herpesvirus infection in fish. [33]

Our study has several deficiencies. As noted by reviewers in our original report, the control subjects were all collected at about the same time from healthy graduate and medical students while the patient samples were collected over more than a year during routine student health clinic operation. Thus, any normal seasonal variation in the relative abundance of organisms in the oropharyngeal microbiome might have been masked. Further, the detection of an organism by PCR in a site does not necessarily indicate that it is a true part of the microbiome of that site as nucleic acids in the oropharynx may not represent live bacteria. We did not perform cultures for *Fn* during this study so confirmatory identification of isolates by conventional microbiological testing could not be done.

As noted previously, the two techniques by which the samples were analyzed were quite different and the results were in some ways different as well. Our previous analysis of samples for *Fn* utilized an in-house developed and validated real-time PCR assay (a modification of the assay published by Aliyu et al [2]) with the *Fn* rpoB gene as target while the microbiome analysis utilizes the 16S rRNA gene with degenerate primers corresponding to more conserved sequences flanking the hypervariable V4 region so as to detect the maximum number of species. The amplified product is then sequenced using Next Generation Sequencing and sequence products are analyzed for OTU content. The specific OTU corresponding to *Fn* does not distinguish between *F*. *necrophorum necrophorum*, a subspecies generally isolated from animal species, and *F*. *necrophorum funduliforme*, the subspecies associated with human disease; however our *Fn* RT-PCR assay was also similarly limited in specificity. Similarly, targets other than the 16S rRNA gene were used for the RT-PCR assays for *Mycoplasma pneumoniae* and β-hemolytic streptococci in our previous report. Nonetheless, the microbiome analysis revealed a much larger set of subjects who were positive for *Fn* (239 of 337 patients and 23 of 30 controls) or GAS (129 of 337 patients and 9 of 30 respectively) than that in our original report, although most were at very low relative abundance. The reason(s) for these discrepancies are unclear. One possibility is that the OTUs corresponding to GAS and *Fn* detected by 16S/NGS represent separate but closely related species that did not amplify with the specific primers utilized in our original analysis. An alternate explanation could be that the sensitivity of the 16S/NGS analysis is significantly higher than the RT-PCR assays. Arguing against this is the fact that the 16S/NGS assay did not detect any of the six samples that were positive by RT-PCR for *M*. *pneumoniae*. However, we believe the failure to detect M. pneumoniae was at least partly due to a 1 base mismatch in the primer set for *M*. *pneumoniae* (as well as *M*. *genitalium*) that was discovered after the testing was complete. Regardless, despite the difference in numbers detected, there was a correlation between the OTU values for *Fn* positive microbiome samples and the Cp value for RT-PCR detection in PCR positive samples.

In summary, the data presented in the current report supplement those in our previous manuscript [9], providing additional confirmatory evidence for a potential causal role of *Fn* in pharyngitis in older adolescents and young adults. High levels of *Fn* and GAS in oropharyngeal samples were associated with lower microbial diversity, and those *Fn* samples with higher Centor scores also had lower diversity, both suggesting a shift in microbial population occurring during the development of an active pharyngeal infection.

## Supporting information

S1 File Diversity in samples with high OTU values for *Fusobacterium necrophorum* or *Streptococcus pyogenes*. (PDF)

## References

[1] Centor RM. Expand the pharyngitis paradigm for adolescents and young adults. Ann Intern Med. 2009;151(11):812–5. doi: 10.7326/0003-4819-151-11-200912010-00011 19949147 10.7326/0003-4819-151-11-200912010-00011

[2] Aliyu SH, Marriott RK, Curran MD, Parmar S, Bentley N, Brown NM, et al Real-time PCR investigation into the importance of Fusobacterium necrophorum as a cause of acute pharyngitis in general practice. J Med Microbiol. 2004;53(Pt 10):1029–35. doi: 10.1099/jmm.0.45648-0 15358827 10.1099/jmm.0.45648-0

[3] Jensen A, Hagelskjaer Kristensen L, Prag J. Detection of Fusobacterium necrophorum subsp. funduliforme in tonsillitis in young adults by real-time PCR. Clin Microbiol Infect. 2007;13(7):695–701. doi: 10.1111/j.1469-0691.2007.01719.x 17403128 10.1111/j.1469-0691.2007.01719.x

[4] Batty A, Wren MW. Prevalence of Fusobacterium necrophorum and other upper respiratory tract pathogens isolated from throat swabs. Br J Biomed Sci. 2005;62(2):66–70. 10.1080/09674845.2005.11732687 15997879

[5] Amess JA, O'Neill W, Giollariabhaigh CN, Dytrych JK. A six-month audit of the isolation of Fusobacterium necrophorum from patients with sore throat in a district general hospital. Br J Biomed Sci. 2007;64(2):63–5. 10.1080/09674845.2007.11732757 17633139

[6] Klug TE, Rusan M, Fuursted K, Ovesen T, Jorgensen AW. A systematic review of Fusobacterium necrophorum-positive acute tonsillitis: prevalence, methods of detection, patient characteristics, and the usefulness of the Centor score. Eur J Clin Microbiol Infect Dis. 2016;27:27. 10.1007/s10096-016-2757-y 27568201

[7] Batty A, Wren MW, Gal M. Fusobacterium necrophorum as the cause of recurrent sore throat: comparison of isolates from persistent sore throat syndrome and Lemierre's disease. J Infect. 2005;51(4):299–306. doi: 10.1016/j.jinf.2004.09.013 16051369 10.1016/j.jinf.2004.09.013

[8] Van TT, Cox LM, Cox ME, Dien Bard J. Prevalence of Fusobacterium necrophorum in Children Presenting with Pharyngitis. J Clin Microbiol. 2017;55(4):1147–53. doi: 10.1128/JCM.02174-16 Epub 2017 Jan 25. 28122872 10.1128/JCM.02174-16 PMC5377842

[9] Centor RM, Atkinson TP, Ratliff AE, Xiao L, Crabb DM, Estrada CA, et al The Clinical Presentation of Fusobacterium-Positive and Streptococcal-Positive Pharyngitis in a University Health Clinic: A Cross-sectional Study. Ann Intern Med. 2015;162(4):241–7. doi: 10.7326/M14-1305 25686164 10.7326/M14-1305

[10] Ehlers Klug T, Rusan M, Fuursted K, Ovesen T. Fusobacterium necrophorum: most prevalent pathogen in peritonsillar abscess in Denmark. Clin Infect Dis. 2009;49(10):1467–72. doi: 10.1086/644616 19842975 10.1086/644616

[11] Jousimies-Somer H, Savolainen S, Makitie A, Ylikoski J. Bacteriologic findings in peritonsillar abscesses in young adults. Clin Infect Dis. 1993;16 Suppl 4:S292–8. 8324134 10.1093/clinids/16.supplement_4.s292

[12] Riordan T. Human infection with Fusobacterium necrophorum (Necrobacillosis), with a focus on Lemierre's syndrome. Clin Microbiol Rev. 2007;20(4):622–59. doi: 10.1128/CMR.00011-07 17934077 10.1128/CMR.00011-07 PMC2176048

[13] Kuppalli K, Livorsi D, Talati NJ, Osborn M. Lemierre's syndrome due to Fusobacterium necrophorum. Lancet Infect Dis. 2012;12(10):808–15. doi: 10.1016/S1473-3099(12)70089-0 22633566 10.1016/S1473-3099(12)70089-0

[14] Brazier JS. Human infections with Fusobacterium necrophorum. Anaerobe. 2006;12(4):165–72. Epub 2005 Dec 22. doi: 10.1016/j.anaerobe.2005.11.003 16962962 10.1016/j.anaerobe.2005.11.003

[15] Centor RM, Witherspoon JM, Dalton HP, Brody CE, Link K. The diagnosis of strep throat in adults in the emergency room. Med Decis Making. 1981;1(3):239–46. doi: 10.1177/0272989X8100100304 6763125 10.1177/0272989X8100100304

[16] Linder JA. Sore Throat: Avoid Overcomplicating the Uncomplicated. Ann Intern Med. 2015;162(12):878–9. doi: 10.7326/L15-5100-2 26075770 10.7326/L15-5100-2

[17] Foy HM. Infections caused by Mycoplasma pneumoniae and possible carrier state in different populations of patients. [Review] [66 refs]. Clinical Infectious Diseases. 1993;17 Suppl 1:S37–46. 8399936 10.1093/clinids/17.supplement_1.s37

[18] Kozich JJ, Westcott SL, Baxter NT, Highlander SK, Schloss PD. Development of a dual-index sequencing strategy and curation pipeline for analyzing amplicon sequence data on the MiSeq Illumina sequencing platform. Applied and environmental microbiology. 2013;79(17):5112–20. doi: 10.1128/AEM.01043-13 23793624 10.1128/AEM.01043-13 PMC3753973

[19] Kumar R, Eipers P, Little RB, Crowley M, Crossman DK, Lefkowitz EJ, et al Getting started with microbiome analysis: sample acquisition to bioinformatics. Curr Protoc Hum Genet. 2014;82:18.8.1–29. 25042718 10.1002/0471142905.hg1808s82 PMC4383038

[20] Kumar R, Maynard CL, Eipers P, Goldsmith KT, Ptacek T, Grubbs JA, et al Colonization potential to reconstitute a microbe community in patients detected early after fecal microbe transplant for recurrent C. difficile. BMC Microbiol. 2016;16:5 doi: 10.1186/s12866-015-0622-2 26758906 10.1186/s12866-015-0622-2 PMC4711103

[21] Lozupone CA, Hamady M, Kelley ST, Knight R. Quantitative and qualitative beta diversity measures lead to different insights into factors that structure microbial communities. Applied and environmental microbiology. 2007;73(5):1576–85. doi: 10.1128/AEM.01996-06 17220268 10.1128/AEM.01996-06 PMC1828774

[22] Navas-Molina JA, Peralta-Sanchez JM, Gonzalez A, McMurdie PJ, Vazquez-Baeza Y, Xu Z, et al Advancing our understanding of the human microbiome using QIIME. Methods Enzymol. 2013;531:371–444. doi: 10.1016/B978-0-12-407863-5.00019-8 24060131 10.1016/B978-0-12-407863-5.00019-8 PMC4517945

[23] Edgar RC. Search and clustering orders of magnitude faster than BLAST. Bioinformatics (Oxford, England). 2010;26(19):2460–1. 10.1093/bioinformatics/btq461 20709691

[24] McDonald D, Price MN, Goodrich J, Nawrocki EP, DeSantis TZ, Probst A, et al An improved Greengenes taxonomy with explicit ranks for ecological and evolutionary analyses of bacteria and archaea. ISME J. 2012;6(3):610–8. doi: 10.1038/ismej.2011.139 22134646 10.1038/ismej.2011.139 PMC3280142

[25] Wang Q, Garrity GM, Tiedje JM, Cole JR. Naive Bayesian classifier for rapid assignment of rRNA sequences into the new bacterial taxonomy. Applied and environmental microbiology. 2007;73(16):5261–7. doi: 10.1128/AEM.00062-07 17586664 10.1128/AEM.00062-07 PMC1950982

[26] Caporaso JG, Bittinger K, Bushman FD, DeSantis TZ, Andersen GL, Knight R. PyNAST: a flexible tool for aligning sequences to a template alignment. Bioinformatics (Oxford, England). 2010;26(2):266–7. 10.1093/bioinformatics/btp636 PMC2804299 19914921

[27] Anderson MJ, Walsh DCI. PERMANOVA, ANOSIM, and the Mantel test in the face of heterogeneous dispersions: What null hypothesis are you testing? Ecological Monographs. 2013;83(4):557–74.

[28] Baselga A. Partitioning the turnover and nestedness components of beta diversity. Global Ecology and Biogeography. 2010;19(1):134–43.

[29] Jensen A, Fago-Olsen H, Sorensen CH, Kilian M. Molecular mapping to species level of the tonsillar crypt microbiota associated with health and recurrent tonsillitis. PLoS One. 2013;8(2):e56418 doi: 10.1371/journal.pone.0056418 23437130 10.1371/journal.pone.0056418 PMC3578847

[30] Ludlam H, Howard J, Kingston B, Donachie L, Foulkes J, Guha S, et al Epidemiology of pharyngeal carriage of Fusobacterium necrophorum. J Med Microbiol. 2009;58(Pt 9):1264–5. doi: 10.1099/jmm.0.006718-0 19528186 10.1099/jmm.0.006718-0

[31] Wood MR, Yu EA, Mehta S. The Human Microbiome in the Fight Against Tuberculosis. Am J Trop Med Hyg. 2017;96(6):1274–84. doi: 10.4269/ajtmh.16-0581 28719264 10.4269/ajtmh.16-0581 PMC5462560

[32] McKenney EA, Greene LK, Drea CM, Yoder AD. Down for the count: Cryptosporidium infection depletes the gut microbiome in Coquerel's sifakas. Microb Ecol Health Dis. 2017;28(1):1335165 doi: 10.1080/16512235.2017.1335165 eCollection 2017. 28740461 10.1080/16512235.2017.1335165 PMC5508644

[33] She R, Li TT, Luo D, Li JB, Yin LY, Li H, et al Changes in the Intestinal Microbiota of Gibel Carp (Carassius gibelio) Associated with Cyprinid herpesvirus 2 (CyHV-2) Infection. Curr Microbiol. 2017;26(10):017–1294. 10.1007/s00284-017-1294-y 28748273

