# Supplementary material for: Analysis of the tonsillar microbiome in young adults with sore throat reveals a high relative abundance of Fusobacterium necrophorum with low diversity
Source: PLoS One. 2018 Jan 19;13(1):e0189423. doi: 10.1371/journal.pone.0189423 (PMC5774679; doi:10.1371/journal.pone.0189423)
Supplement: S1 File — (PDF) [file pone.0189423.s001.pdf]

Diversity in Samples with High OTU Values for Fusobacterium necrophorum* or *Streptococcus pyogenes

Input data link:

The raw data were downloaded from UAB Microbiome Facility:

https://genome-bmidb.ad.uab.edu/ccts/bmi/microbiome/result2016/REDO/Centor500/microbiome_report.html

I. Fusobacterium necrophorum

Analysis of Fn Positive Samples Using Positive_high Cutoff for Samples with OTU Values > 0.1

Total samples 367

control: 30

positive-high: 18

positive-low: 199

negative: 120

Table A. Alpha Diversity.

Global Comparison

	Stratified by Group				p
	Control	Negative	Positive high	Positive low	
n	30	120	18	199	
chao1 (mean (sd))	260.24 (41.29)	253.18 (36.27)	209.38 (53.68)	257.08 (42.47)	<0.001
observed_species (mean (sd))	210.60 (30.04)	205.10 (32.28)	165.72 (46.00)	210.69 (34.92)	<0.001
PD_whole_tree (mean (sd))	14.18 (1.43)	13.33 (1.56)	12.41 (2.12)	13.76 (1.87)	0.001
shannon (mean (sd))	4.52 (0.49)	4.34 (0.48)	3.06 (1.08)	4.39 (0.55)	<0.001
simpson (mean (sd))	0.91 (0.05)	0.89 (0.06)	0.67 (0.20)	0.90 (0.08)	<0.001

Table B. Check Assumptions: Homogeneity of Variance (Levene's Test).

P values of Homogeneity of Variance

	p value
chao1	0.19498
observed species	0.05975
PD whole tree	0.11428
shannon	0.00000
simpson	0.00000

For chao1, observed species and pd whole tree -- Variances equal

For Shannon and Simpson -- Variances unequal

Table C. P Values of ANOVA.

	p value
chao1	0.00005
observed species	0.00000
PD whole tree	0.00133
shannon	0.00003
simpson	0.00016

Conclusion: At least one group mean is different from the others for five Alpha diversities.

Table D. Chao1 (comparison in Tukey HSD).

Tukey multiple comparisons of means (95% family-wise confidence level)				
	diff	lwr	upr	adj-p
Negative vs Control	-7.0529	-28.6863	14.5805	0.8346
Positive_high vs Control	-50.8548	-82.4525	-19.2572	0.0002
Positive_low vs Control	-3.1551	-23.9120	17.6018	0.9795
Positive_high vs Negative	-43.8019	-70.5901	-17.0137	0.0002
Positive_low vs Negative	3.8978	-8.3514	16.1470	0.8444
Positive_low vs Positive_high	47.6997	21.6143	73.7851	0.0000

Table E. Observed Species (comparison in Tukey HSD).

Tukey multiple comparisons of means (95% family-wise confidence level)				
	diff	lwr	upr	adj-p
Negative vs Control	-5.5000	-23.5802	12.5803	0.8612
Positive_high vs Control	-44.8778	-71.2857	-18.4699	0.0001
Positive_low vs Control	0.0935	-17.2542	17.4411	1.0000
Positive_high vs Negative	-39.3778	-61.7662	-16.9894	0.0000
Positive_low vs Negative	5.5935	-4.6439	15.8308	0.4938
Positive_low vs Positive_high	44.9712	23.1702	66.7723	0.0000

Table F. PD Whole Tree (comparison in Tukey HSD).

Tukey multiple comparisons of means (95% family-wise confidence level)				
	diff	lwr	upr	adj-p
Negative vs Control	-0.8504	-1.7743	0.0736	0.0837
Positive_high vs Control	-1.7655	-3.1150	-0.4160	0.0045
Positive_low vs Control	-0.4207	-1.3072	0.4657	0.6114
Positive_high vs Negative	-0.9151	-2.0592	0.2290	0.1668
Positive_low vs Negative	0.4296	-0.0935	0.9528	0.1488
Positive_low vs Positive_high	1.3447	0.2307	2.4588	0.0106

Table G. Shannon (Pairwise comparisons with the Bonferroni correction).

Pairwise comparisons using t tests with non-pooled SD			
	Control	Negative	Positive_high
Negative	0.41748	-	-
Positive_high	0.00013	0.00064	-
Positive_low	1	1	0.00043

Table H. Simpson (Pairwise comparisons with the Bonferroni correction).

Pairwise comparisons using t tests with non-pooled SD			
	Control	Negative	Positive_high

Negative	1.0000	-	-
Positive_high	0.0007	0.0012	-
Positive_low	1.0000	1.0000	0.0011

Beta Diversity Analysis of *Fn* Positive Samples

Figure A. Bray Curtis Analysis of *Fn* Positive Samples Using OTU > 0.1 as Positive_high Cutoff.

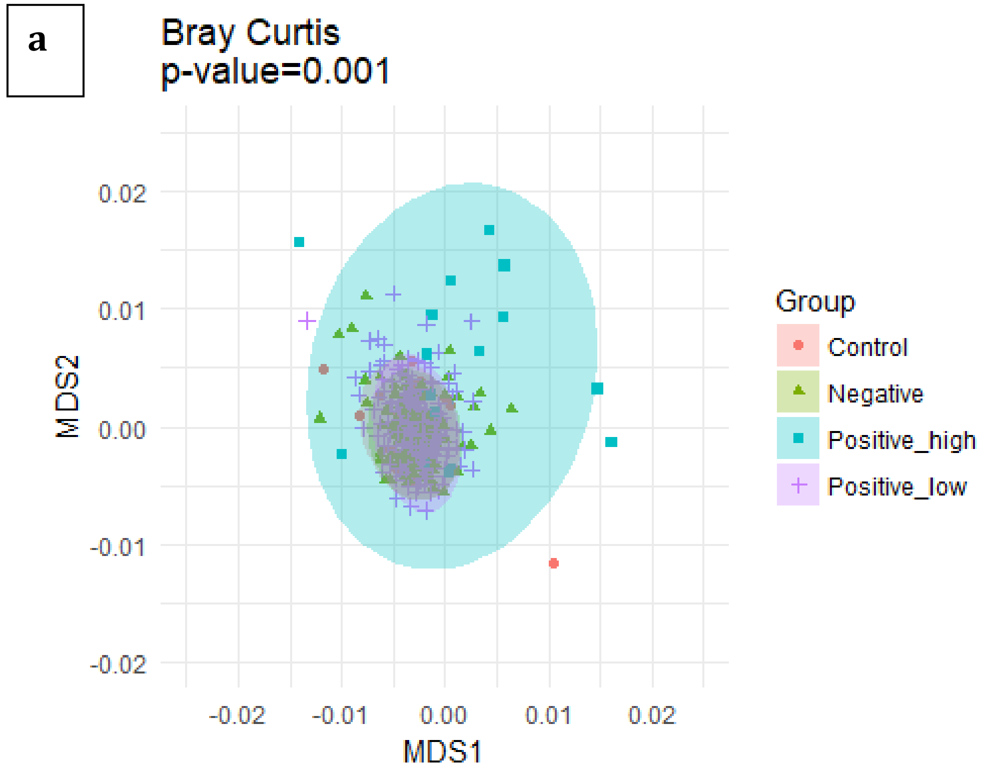

b

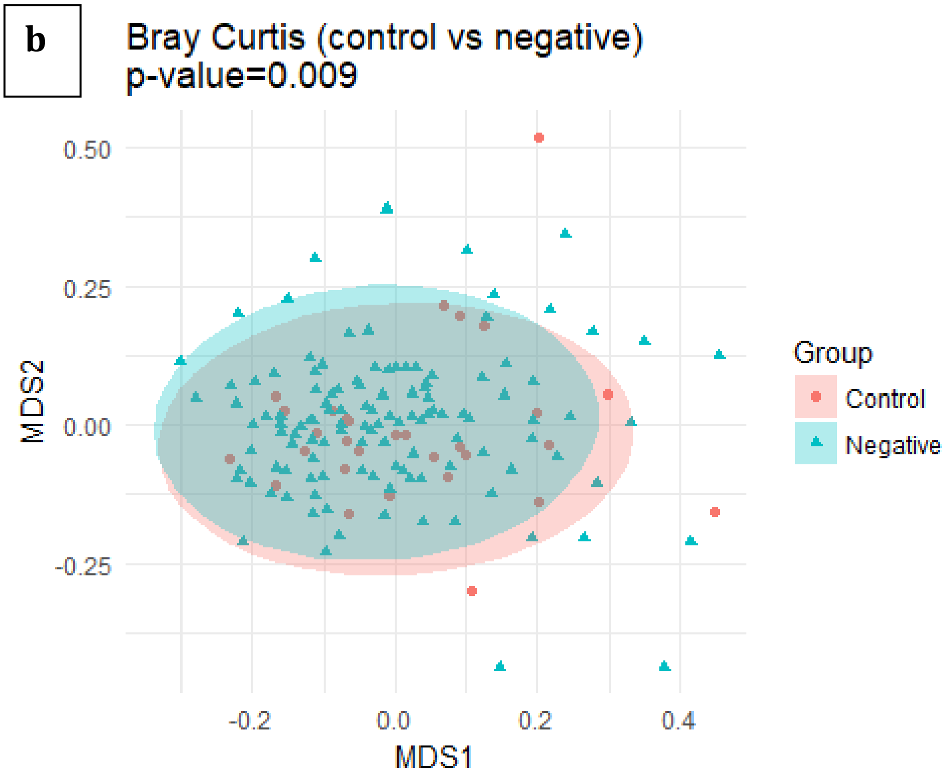
Bray Curtis (control vs negative)
p-value=0.009
0.50
0.25
0.00
-0.25
MDS2
-0.2
0.0
0.2
0.4
MDS1
Group
Control
Negative

c

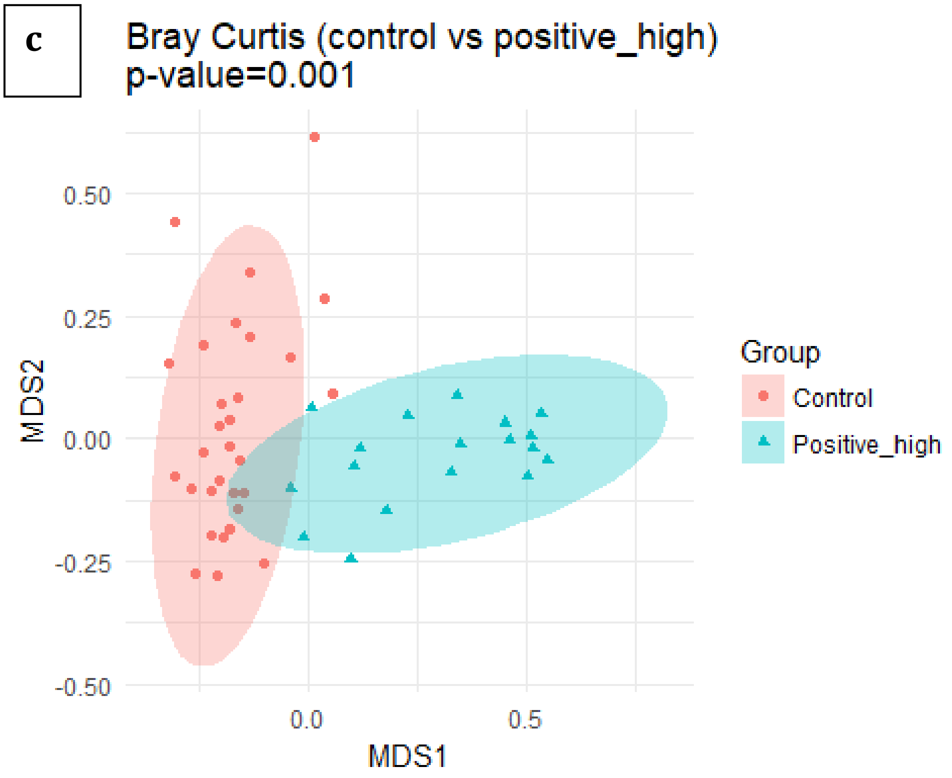
Bray Curtis (control vs positive_high)
p-value=0.001
0.50
0.25
0.00
-0.25
-0.50
MDS2
0.0
0.5
MDS1
Group
Control
Positive_high

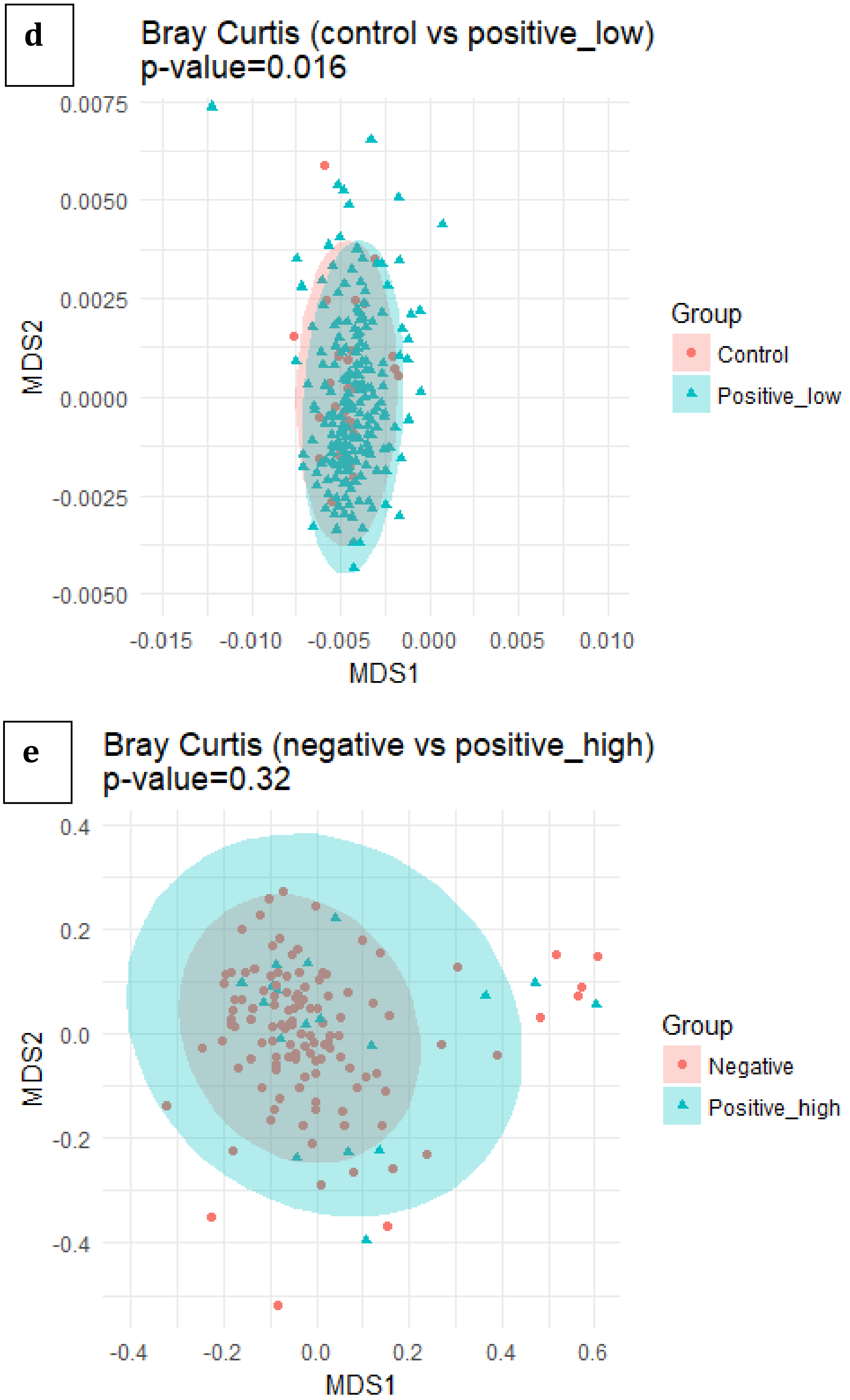
d
Bray Curtis (control vs positive_low)
p-value=0.016
0.0075
0.0050
0.0025
0.0000
-0.0025
-0.0050
MDS2
-0.015
-0.010
-0.005
0.000
0.005
0.010
MDS1
Group
Control
Positive_low
e
Bray Curtis (negative vs positive_high)
p-value=0.32
0.4
0.2
0.0
-0.2
-0.4
MDS2
-0.4
-0.2
0.0
0.2
0.4
0.6
MDS1
Group
Negative
Positive_high

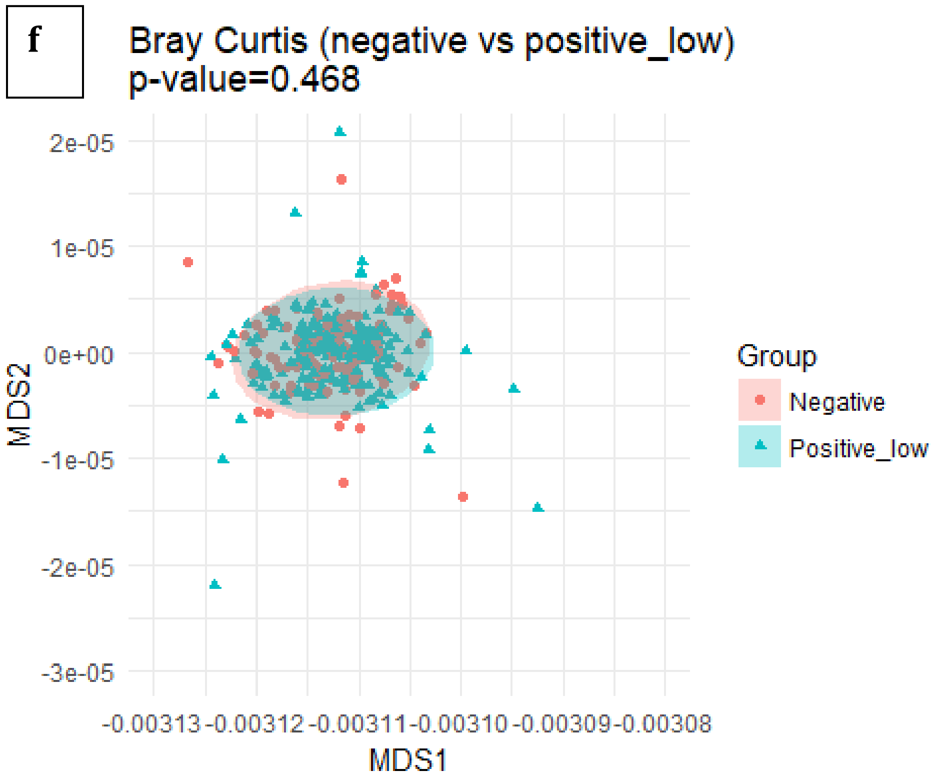
f
Bray Curtis (negative vs positive_low)
p-value=0.468
2e-05
1e-05
0e+00
-1e-05
-2e-05
-3e-05
MDS2
-0.00313
-0.00312
-0.00311
-0.00310
-0.00309
-0.00308
MDS1
Group
Negative
Positive_low

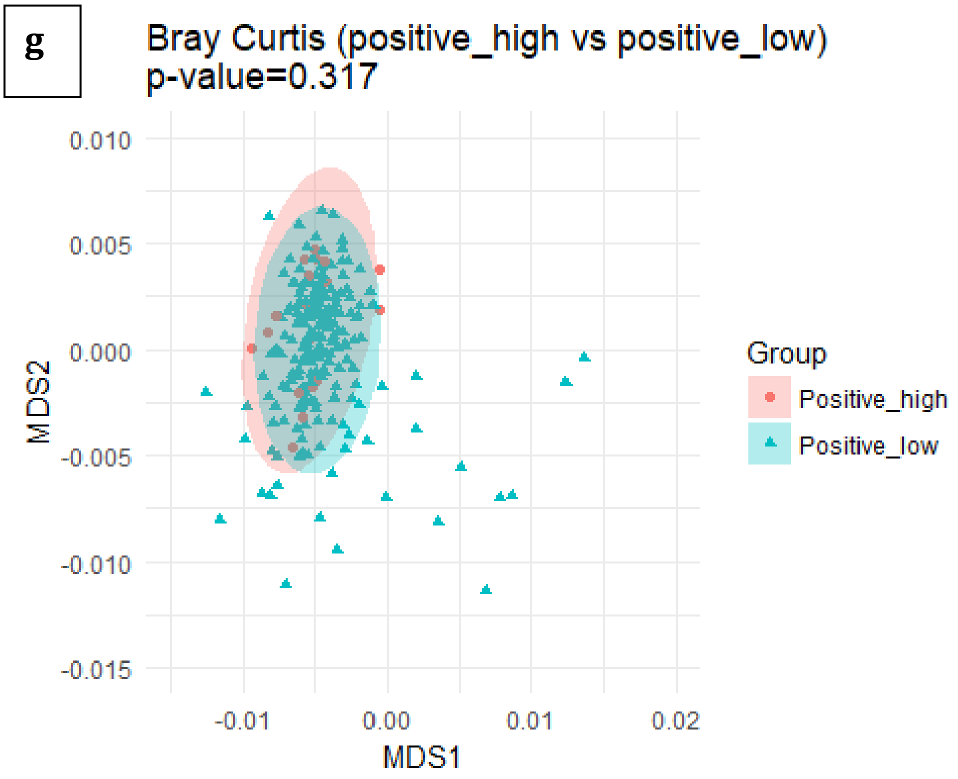
g
Bray Curtis (positive_high vs positive_low)
p-value=0.317
0.010
0.005
0.000
-0.005
-0.010
-0.015
MDS2
-0.01
0.00
0.01
0.02
MDS1
Group
Positive_high
Positive_low

Fig B. Weighted Unifrac Analysis of *Fn* Positive Samples Using OTU > 0.1 as Positive_high Cutoff.

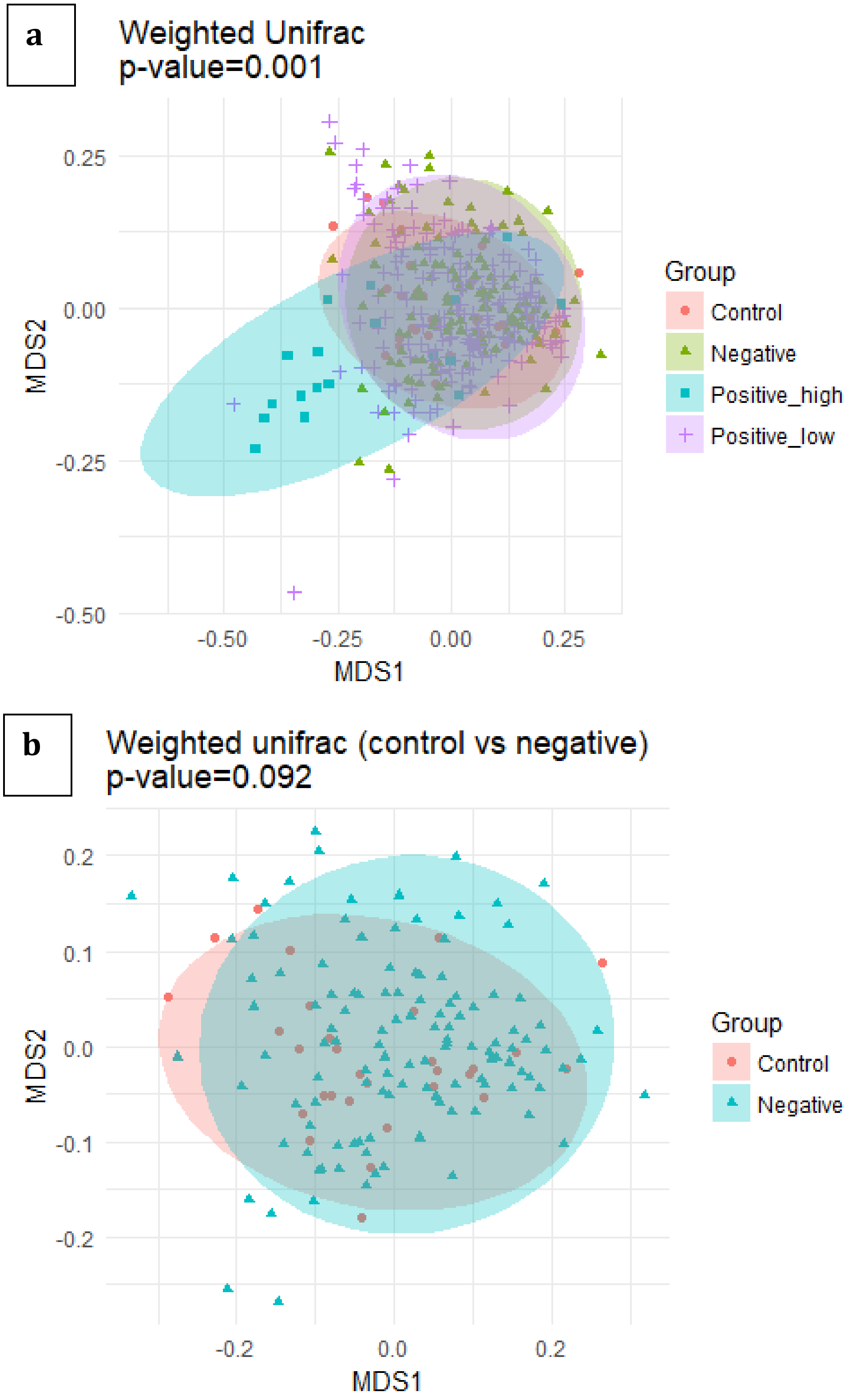

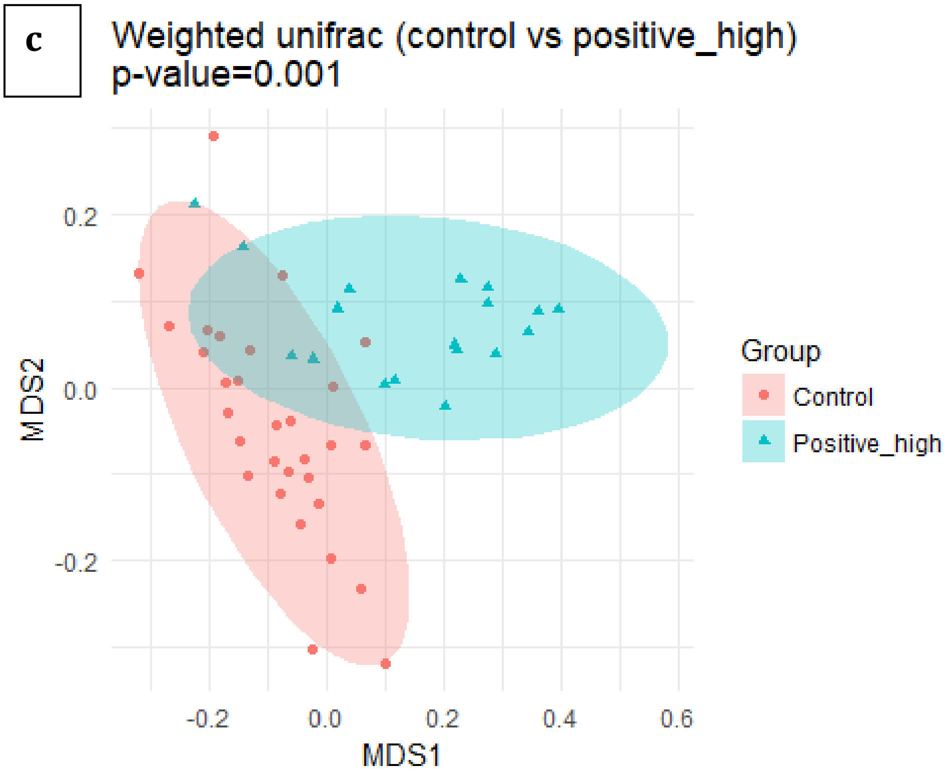
c
Weighted unifrac (control vs positive_high)
p-value=0.001
0.2
0.0
-0.2
MDS2
-0.2
0.0
0.2
0.4
0.6
MDS1
Group
Control
Positive_high

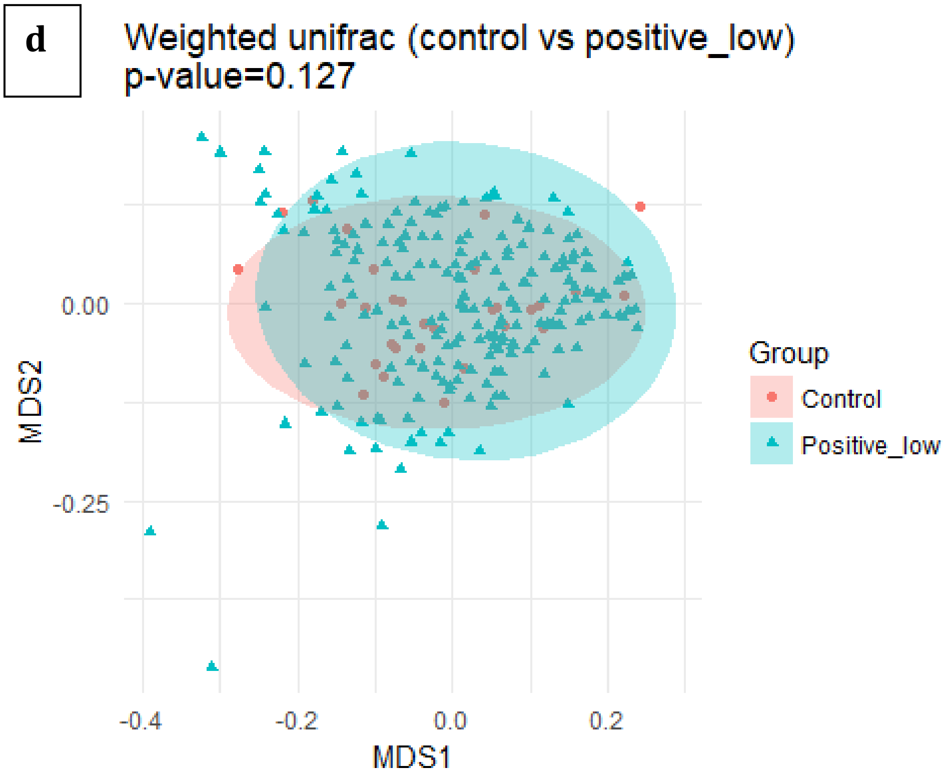
d
Weighted unifrac (control vs positive_low)
p-value=0.127
0.00
-0.25
MDS2
-0.4
-0.2
0.0
0.2
MDS1
Group
Control
Positive_low

e

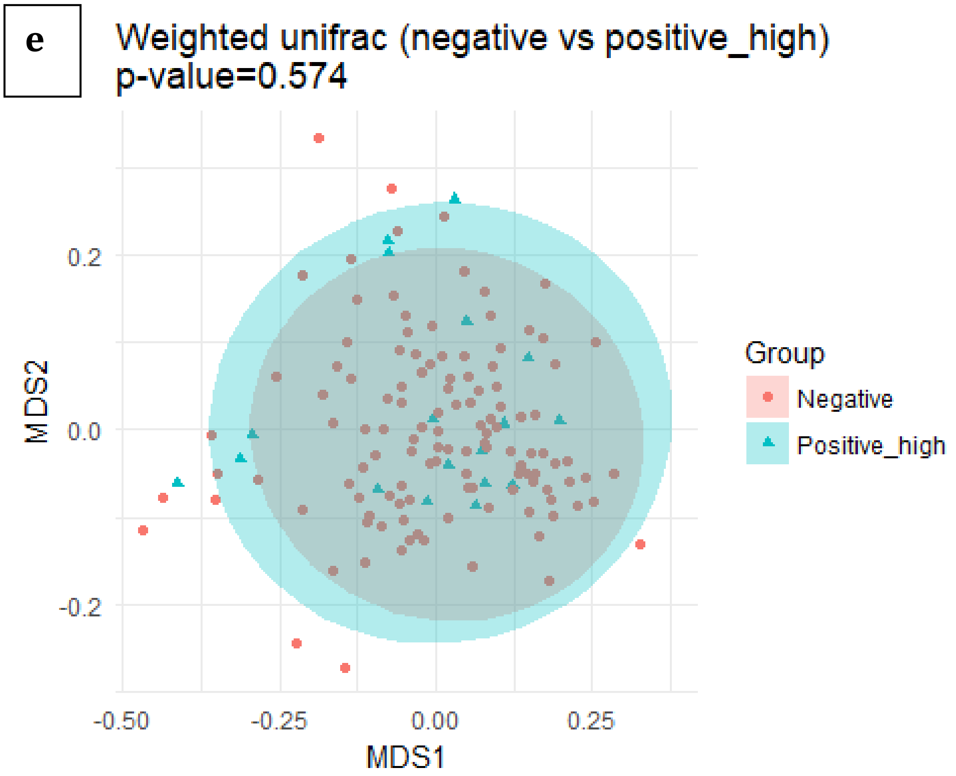
Weighted unifrac (negative vs positive_high)
p-value=0.574
MDS2
0.2
0.0
-0.2
-0.50
-0.25
0.00
0.25
MDS1
Group
Negative
Positive_high

f

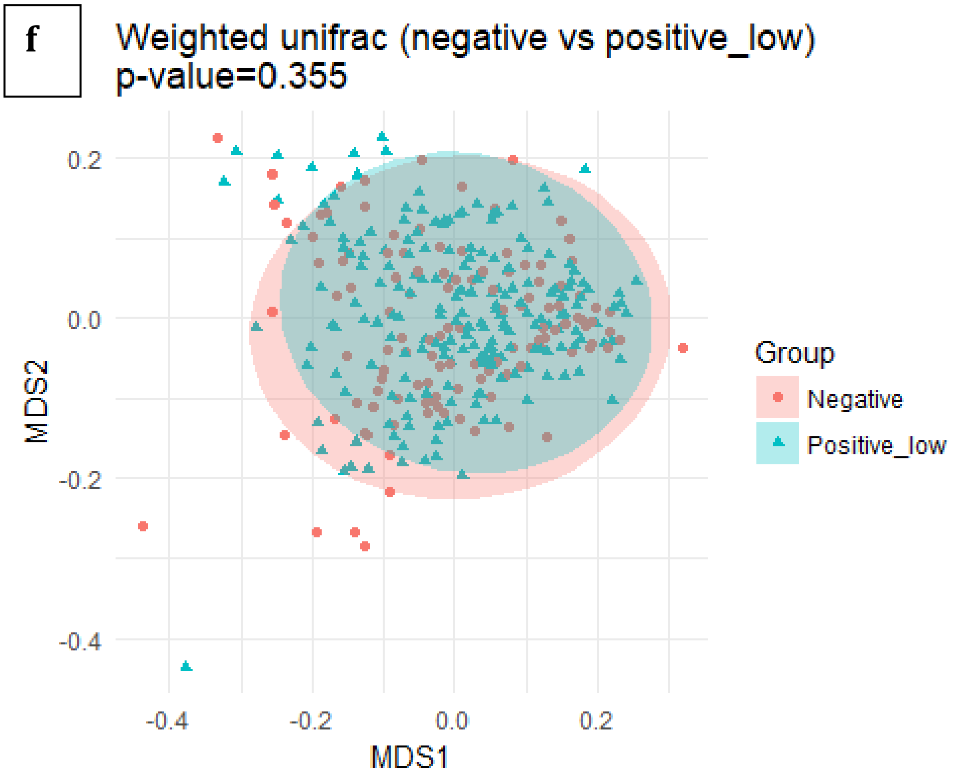
Weighted unifrac (negative vs positive_low)
p-value=0.355
MDS2
0.2
0.0
-0.2
-0.4
-0.4
-0.2
0.0
0.2
MDS1
Group
Negative
Positive_low

g

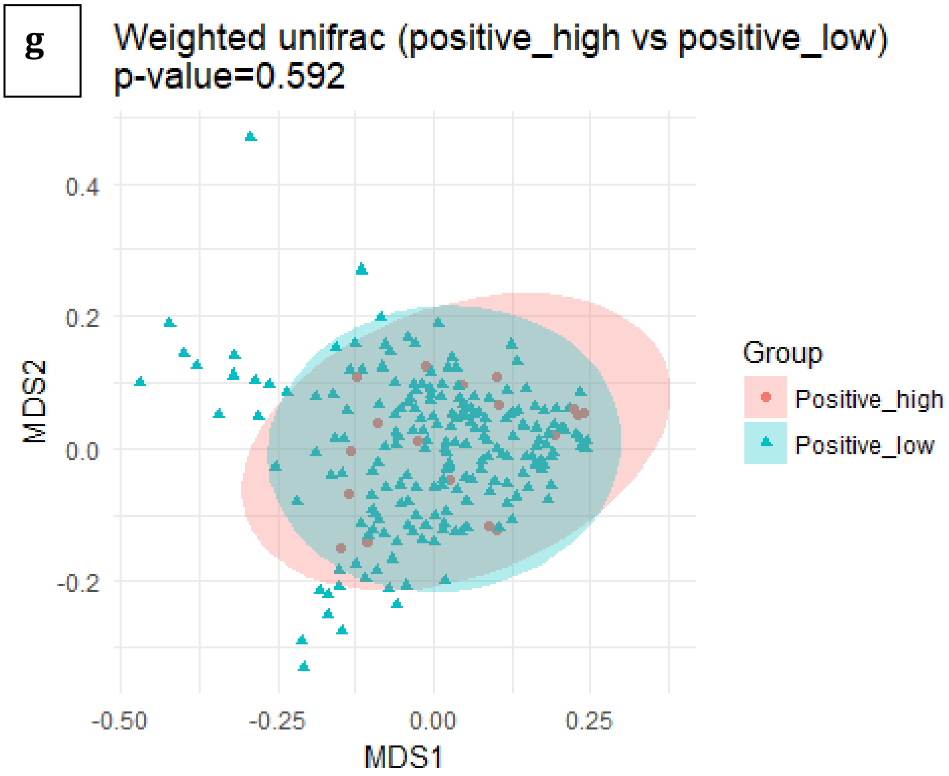
Weighted unifrac (positive_high vs positive_low)
p-value=0.592
MDS2
MDS1
0.4
0.2
0.0
-0.2
-0.50
-0.25
0.00
0.25
Group
Positive_high
Positive_low

Fig C. Unweighted Unifrac Analysis of *Fn* Positive Samples Using OTU > 0.1 as Positive_high Cutoff.

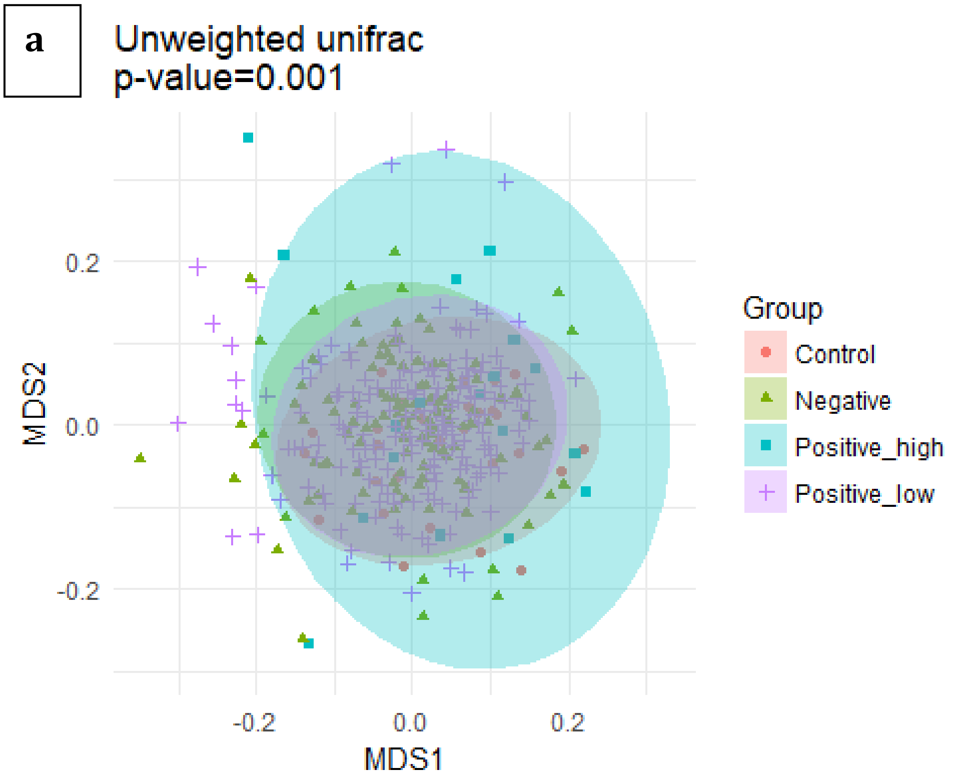

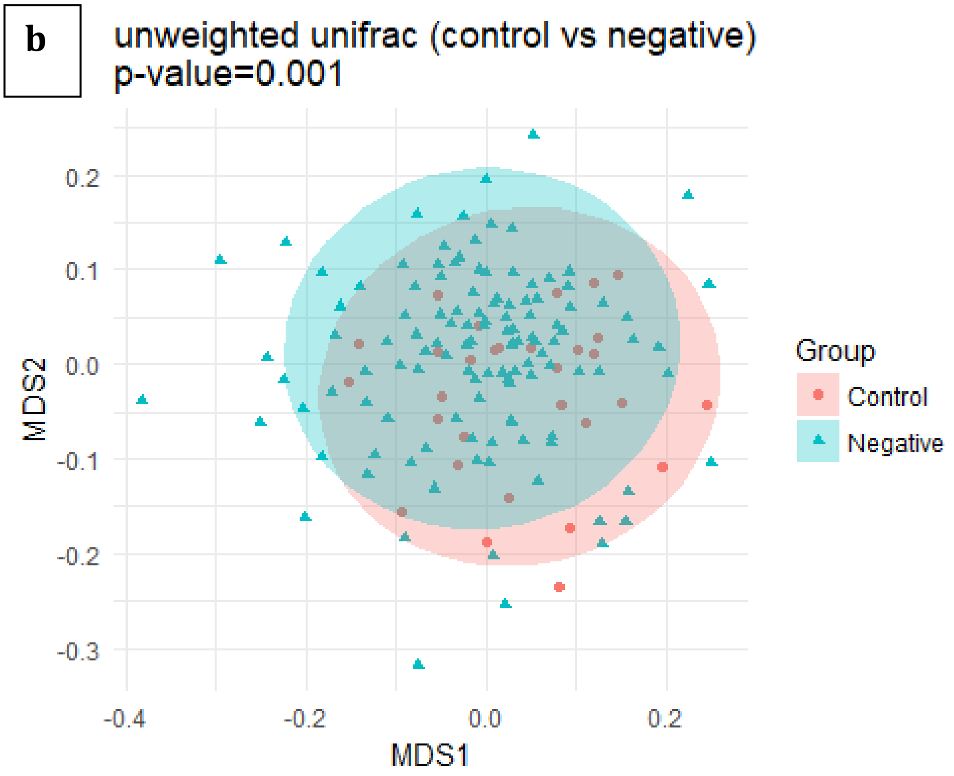

c

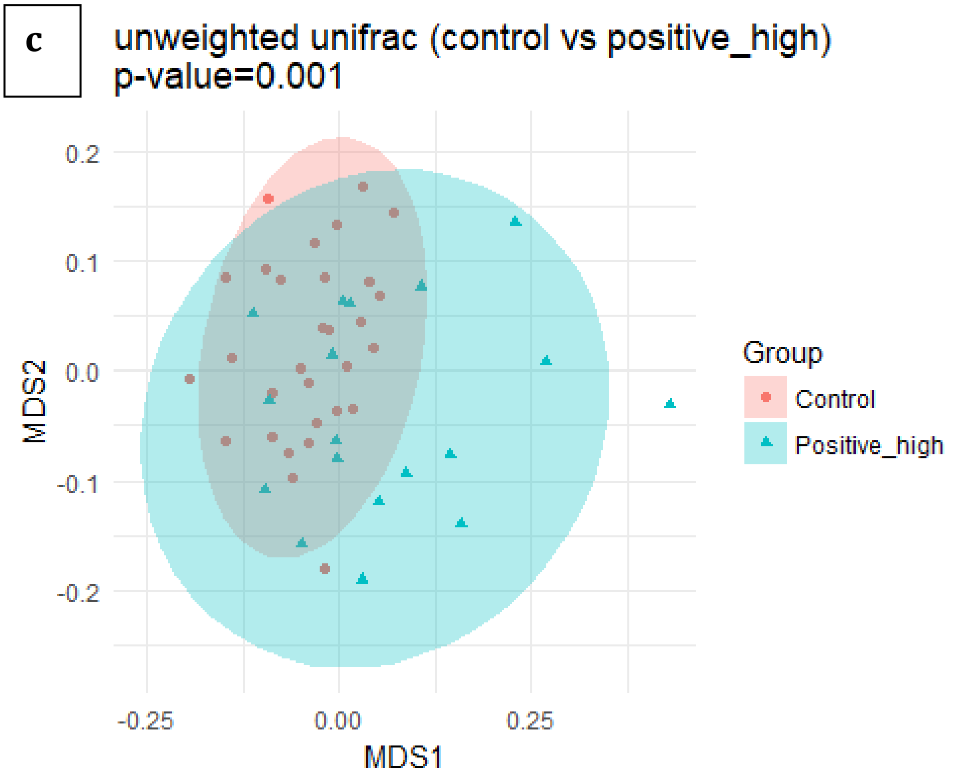
unweighted unifrac (control vs positive_high)
p-value=0.001
MDS2
MDS1
0.2
0.1
0.0
-0.1
-0.2
-0.25
0.00
0.25
Group
Control
Positive_high

d

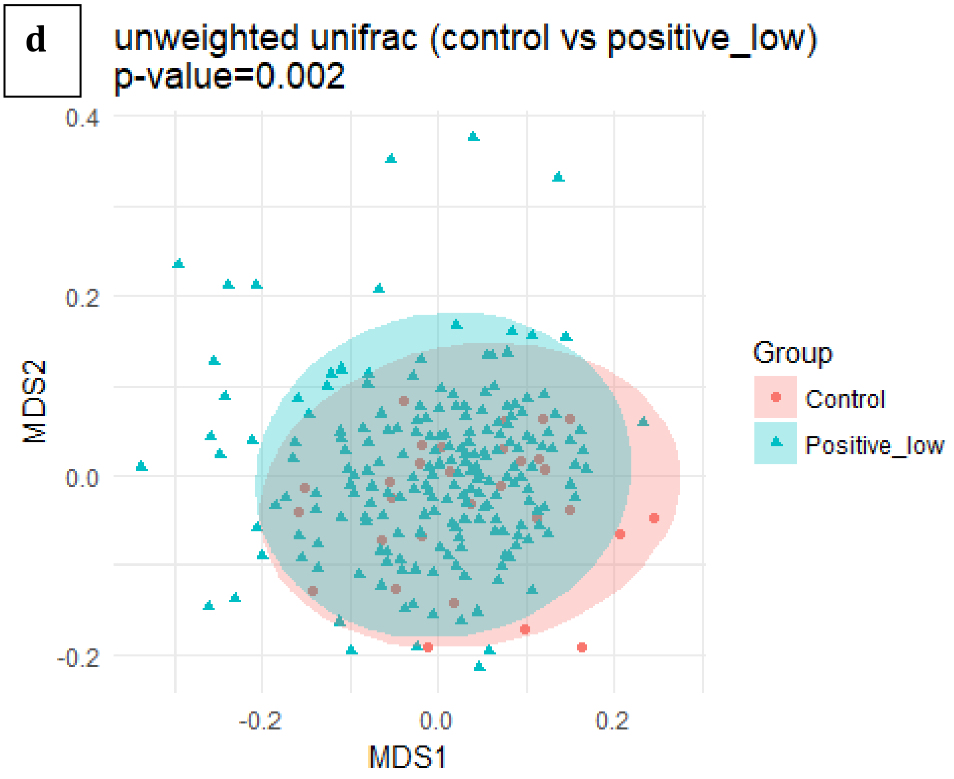
unweighted unifrac (control vs positive_low)
p-value=0.002
MDS2
MDS1
0.4
0.2
0.0
-0.2
-0.2
0.0
0.2
Group
Control
Positive_low

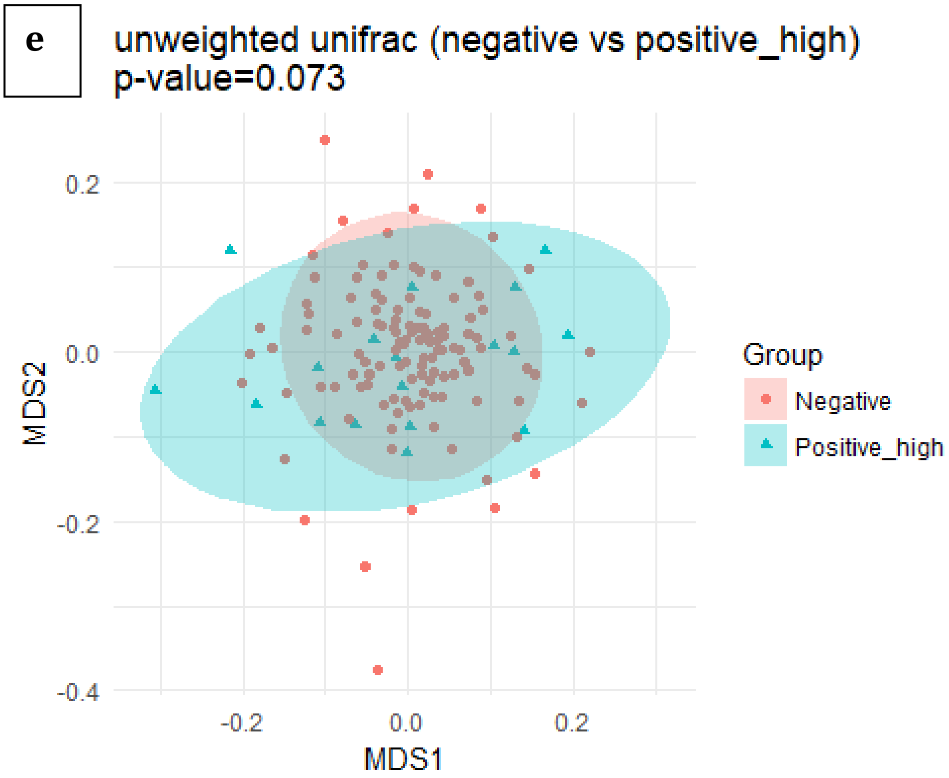
e
unweighted unifrac (negative vs positive_high)
p-value=0.073
MDS2
MDS1
0.2
0.0
-0.2
-0.4
-0.2
0.0
0.2
Group
Negative
Positive_high

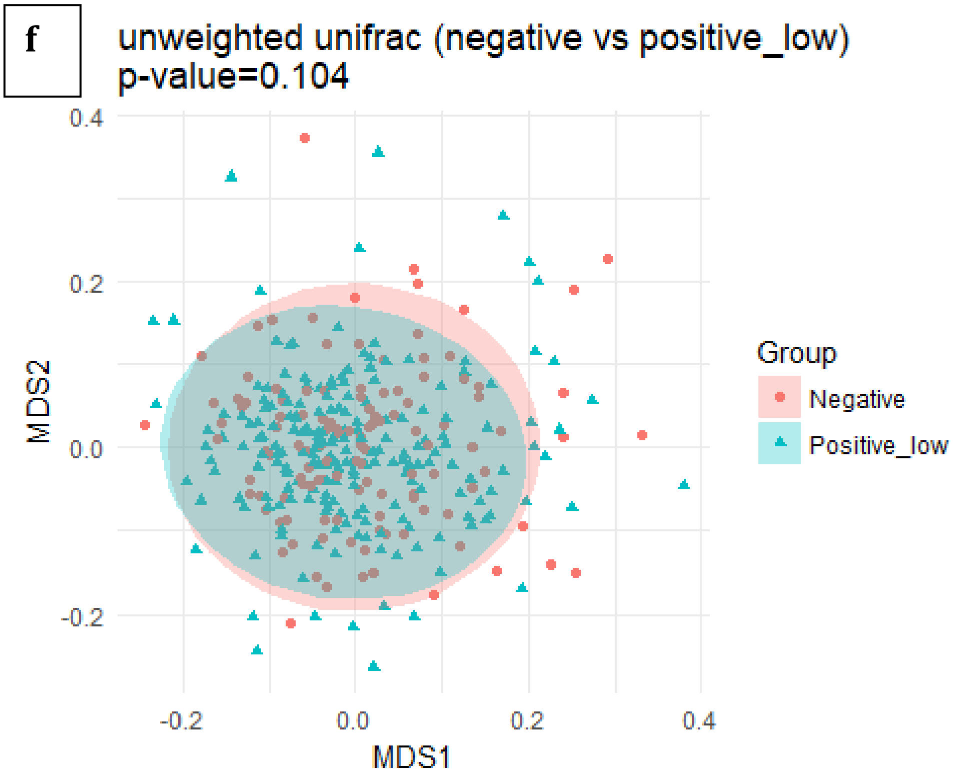
f
unweighted unifrac (negative vs positive_low)
p-value=0.104
MDS2
MDS1
0.4
0.2
0.0
-0.2
-0.2
0.0
0.2
0.4
Group
Negative
Positive_low

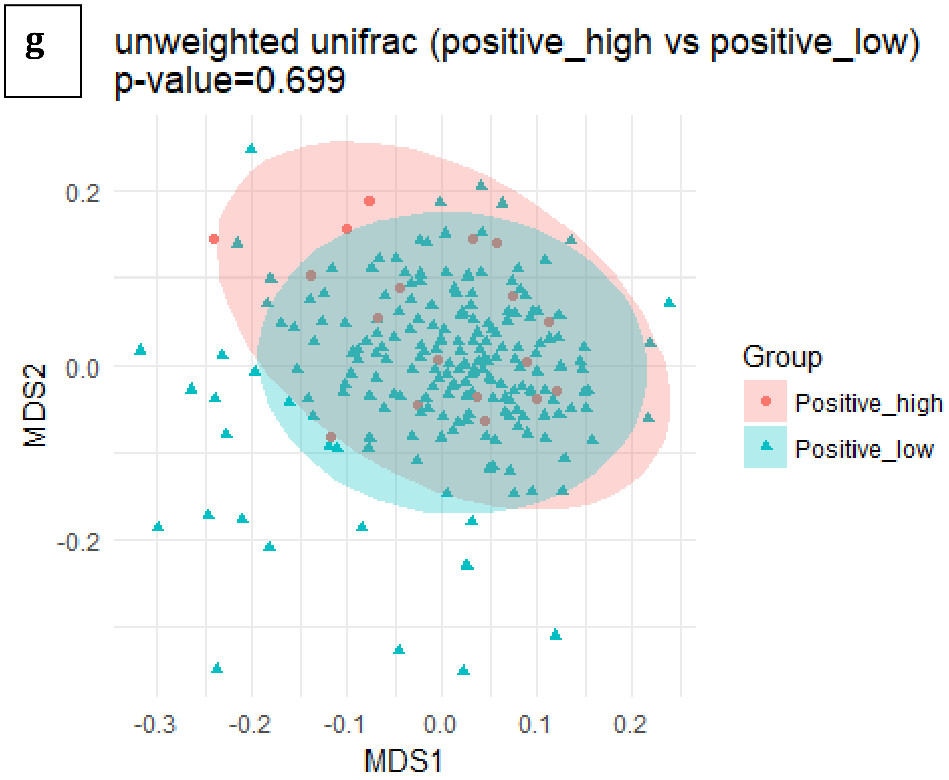

Permutational Multivariate Analysis of Variance (PERMANOVA)

Table I. P values of Overall PERMANOVA.

	p-value
Bray Curtis	0.001
Weighted unifrac	0.001
Unweighted unifrac	0.001

Table J. P Values of Pair-Wise PERMANOVA.

	p-value
Bray Curtis	
control vs negative	0.009
control vs positive_high	0.001
control vs positive_low	0.016

negative vs positive_high	0.320
negative vs positive_low	0.468
positive_high vs positive_low	0.317

Weighted unifrac

control vs negative	0.092
control vs positive_high	0.001
control vs positive_low	0.127
negative vs positive_high	0.574
negative vs positive_low	0.355
positive_high vs positive_low	0.592

Unweighted unifrac

control vs negative	0.001
control vs positive_high	0.001
control vs positive_low	0.002
negative vs positive_high	0.073
negative vs positive_low	0.104
positive_high vs positive_low	0.699

II. GAS (Streptococcus pyogenes):

Analysis of GAS Positive Samples Using Positive_high Cutoff for Samples with OTU Values > 0.05

Total samples 367

control: 30

positive-high: 13

positive-low: 116

negative: 208

Table K. Alpha Diversity.

Global Comparison

	Stratified by Group				adj-p
	Control	Negative	Positive_high	Positive_low	
n	30	208	13	116	
chao1 (mean (sd))	260.24 (41.29)	252.44 (40.64)	252.64 (33.87)	254.47 (46.09)	0.814
observed_species (mean (sd))	210.60 (30.04)	205.72 (34.32)	201.54 (33.34)	207.88 (39.26)	0.821
PD_whole_tree (mean (sd))	14.18 (1.43)	13.37 (1.73)	13.88 (1.85)	13.78 (1.92)	0.044
shannon (mean (sd))	4.52 (0.49)	4.30 (0.60)	4.25 (0.61)	4.30 (0.70)	0.312
simpson (mean (sd))	0.91 (0.05)	0.89 (0.09)	0.87 (0.08)	0.88 (0.11)	0.437

Table L. Check Assumptions: Homogeneity of Variance (Levene's Test).

P values of Homogeneity of Variance

	p value
chao1	0.62695
observed species	0.47512
PD whole tree	0.42443
shannon	0.57345
simpson	0.47314

For chao1, observed species, pd whole tree, Shannon and Simpson-- Variances equal

Table M. P Values of ANOVA.

	p value
chao1	0.81367
observed species	0.82111
PD whole tree	0.04422
shannon	0.31171
simpson	0.43700

Table N. Chao1 (comparison in Tukey HSD).

Tukey multiple comparisons of means (95% family-wise confidence level)				
	diff	lwr	upr	adj-p
Negative vs Control	-7.7992	-29.1176	13.5192	0.7810
Positive_high vs Control	-7.5916	-43.8377	28.6544	0.9490
Positive_low vs Control	-5.7644	-28.1230	16.5942	0.9100
Positive_high vs Negative	0.2076	-30.9994	31.4145	1.0000
Positive_low vs Negative	2.0348	-10.6146	14.6843	0.9759
Positive_low vs Positive_high	1.8272	-30.0994	33.7539	0.9988

Table O. Observed Species (comparison in Tukey HSD).

Tukey multiple comparisons of means (95% family-wise confidence level)				
	diff	lwr	upr	adj-p
Negative vs Control	-4.8837	-22.8372	13.0699	0.8962
Positive_high vs Control	-9.0615	-39.5866	21.4635	0.8696
Positive_low vs Control	-2.7207	-21.5503	16.1089	0.9823
Positive_high vs Negative	-4.1779	-30.4592	22.1034	0.9767
Positive_low vs Negative	2.1630	-8.4899	12.8158	0.9533
Positive_low vs Positive_high	6.3408	-20.5465	33.2282	0.9293

Table P. PD Whole Tree (comparison in Tukey HSD).

Tukey multiple comparisons of means (95% family-wise confidence level)				
	diff	lwr	upr	adj-p
Negative vs Control	-0.8064	-1.6996	0.0869	0.0932
Positive_high vs Control	-0.2966	-1.8153	1.2221	0.9581
Positive_low vs Control	-0.3963	-1.3331	0.5405	0.6948
Positive_high vs Negative	0.5098	-0.7978	1.8173	0.7459
Positive_low vs Negative	0.4101	-0.1199	0.9401	0.1910
Positive_low vs Positive_high	-0.0997	-1.4374	1.2380	0.9975

Table Q. Shannon (Pairwise comparisons with the Bonferroni correction).

Tukey multiple comparisons of means (95% family-wise confidence level)				
	diff	lwr	upr	adj-p
Negative vs Control	-0.2247	-0.5418	0.0925	0.2616
Positive_high vs Control	-0.2711	-0.8103	0.2682	0.5652
Positive_low vs Control	-0.2184	-0.5510	0.1142	0.3280
Positive_high vs Negative	-0.0464	-0.5106	0.4179	0.9940
Positive_low vs Negative	0.0063	-0.1819	0.1945	0.9998
Positive_low vs Positive_high	0.0527	-0.4223	0.5276	0.9918

Table R. Simpson (Pairwise comparisons with the Bonferroni correction).

Tukey multiple comparisons of means (95% family-wise confidence level)				
	diff	lwr	upr	adj-p
Negative vs Control	-0.0207	-0.0673	0.0259	0.6619
Positive_high vs Control	-0.0337	-0.1129	0.0456	0.6922
Positive_low vs Control	-0.0296	-0.0785	0.0193	0.4016
Positive_high vs Negative	-0.0130	-0.0812	0.0553	0.9611
Positive_low vs Negative	-0.0089	-0.0366	0.0187	0.8393
Positive_low vs Positive_high	0.0041	-0.0657	0.0739	0.9988

Beta Diversity Analysis of GAS Positive Samples Using OTU Values > 0.05 as GAS Positive_High

Fig D. Bray Curtis Analysis of GAS Positive Samples Using OTU > 0.05 as Positive_high Cutoff.

a

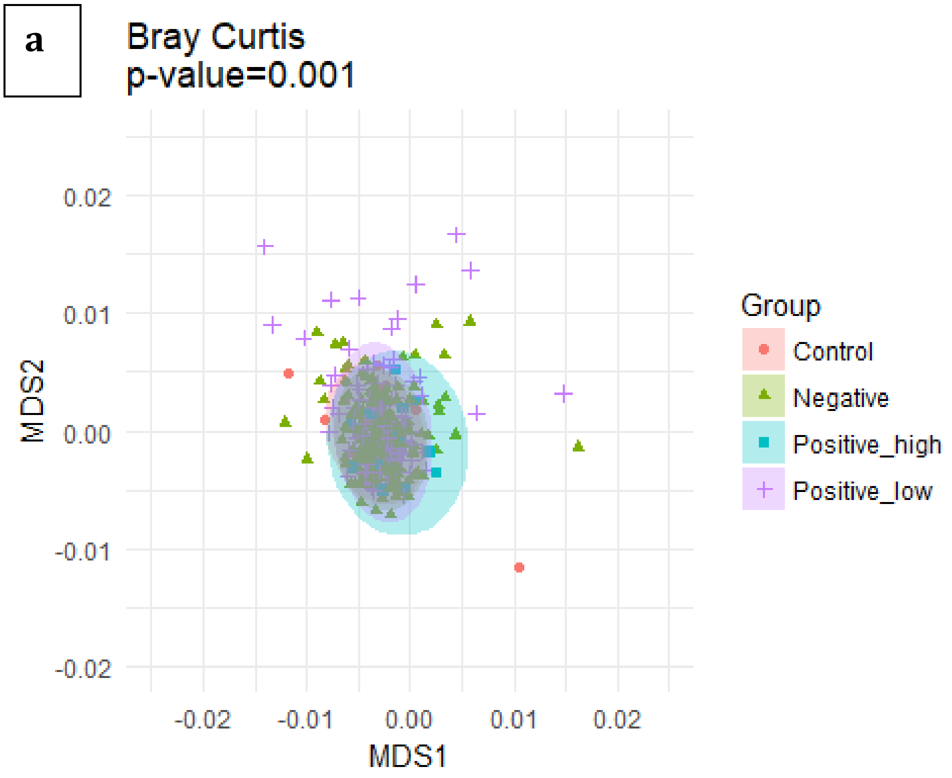

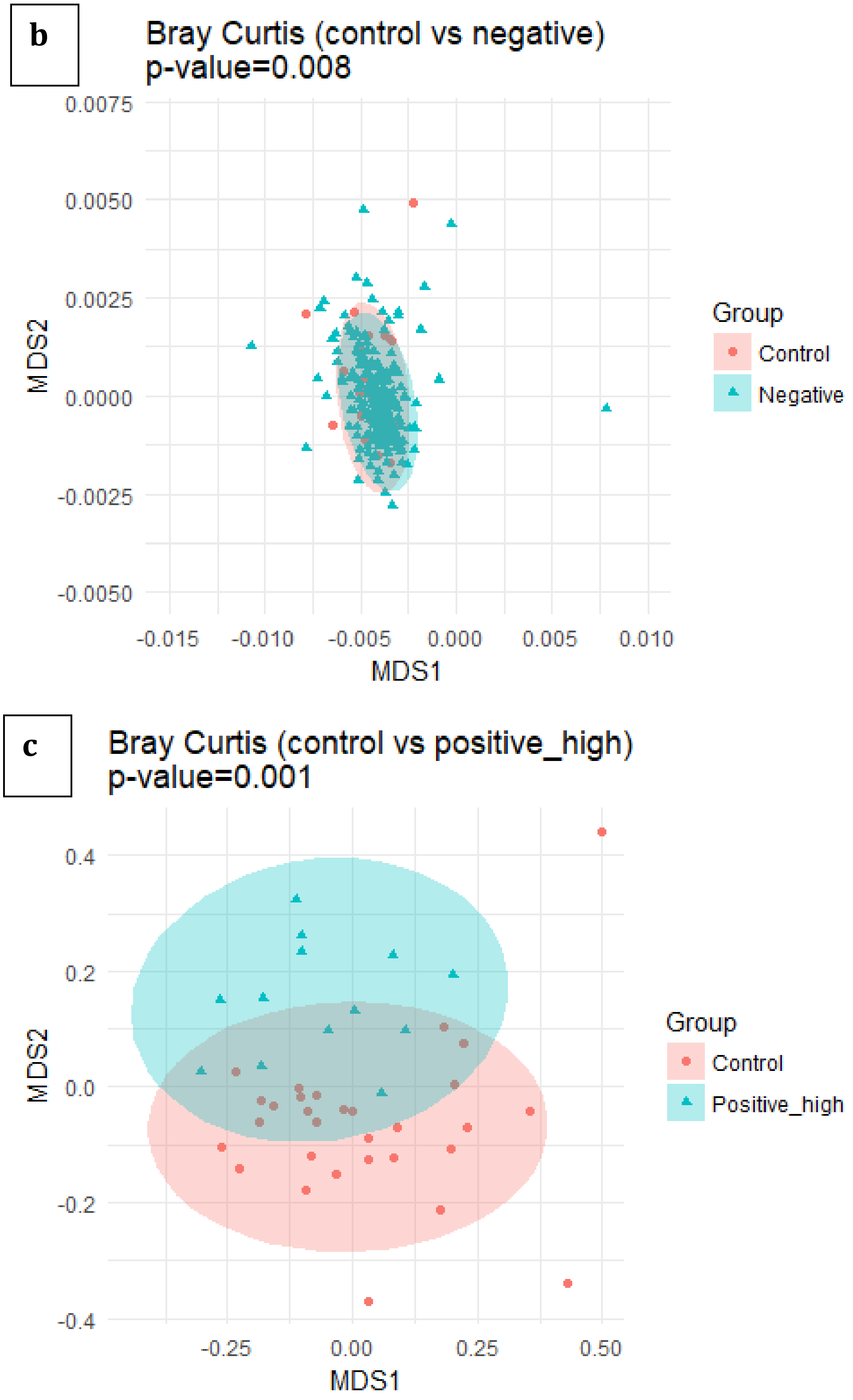

b
Bray Curtis (control vs negative)
p-value=0.008
0.0075
0.0050
0.0025
0.0000
-0.0025
-0.0050
MDS2
-0.015
-0.010
-0.005
0.000
0.005
0.010
MDS1
Group
Control
Negative
c
Bray Curtis (control vs positive_high)
p-value=0.001
0.4
0.2
0.0
-0.2
-0.4
MDS2
-0.25
0.00
0.25
0.50
MDS1
Group
Control
Positive_high

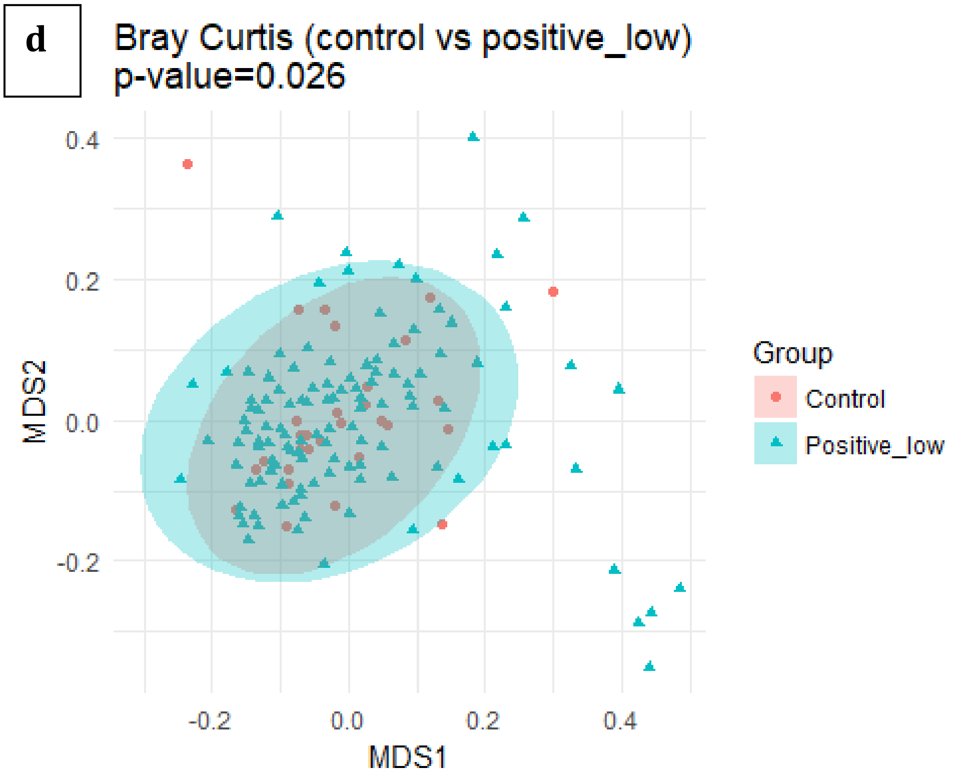

d
Bray Curtis (control vs positive_low)
p-value=0.026
0.4
0.2
0.0
-0.2
MDS2
-0.2
0.0
0.2
0.4
MDS1
Group
Control
Positive_low

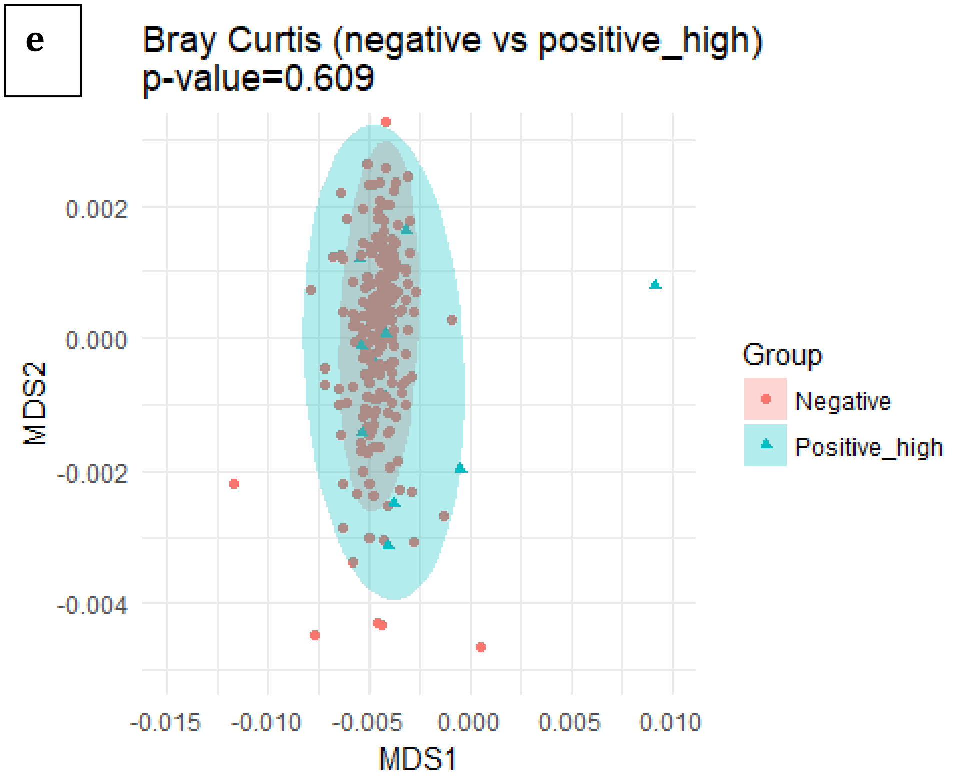

e
Bray Curtis (negative vs positive_high)
p-value=0.609
0.002
0.000
-0.002
-0.004
MDS2
-0.015
-0.010
-0.005
0.000
0.005
0.010
MDS1
Group
Negative
Positive_high

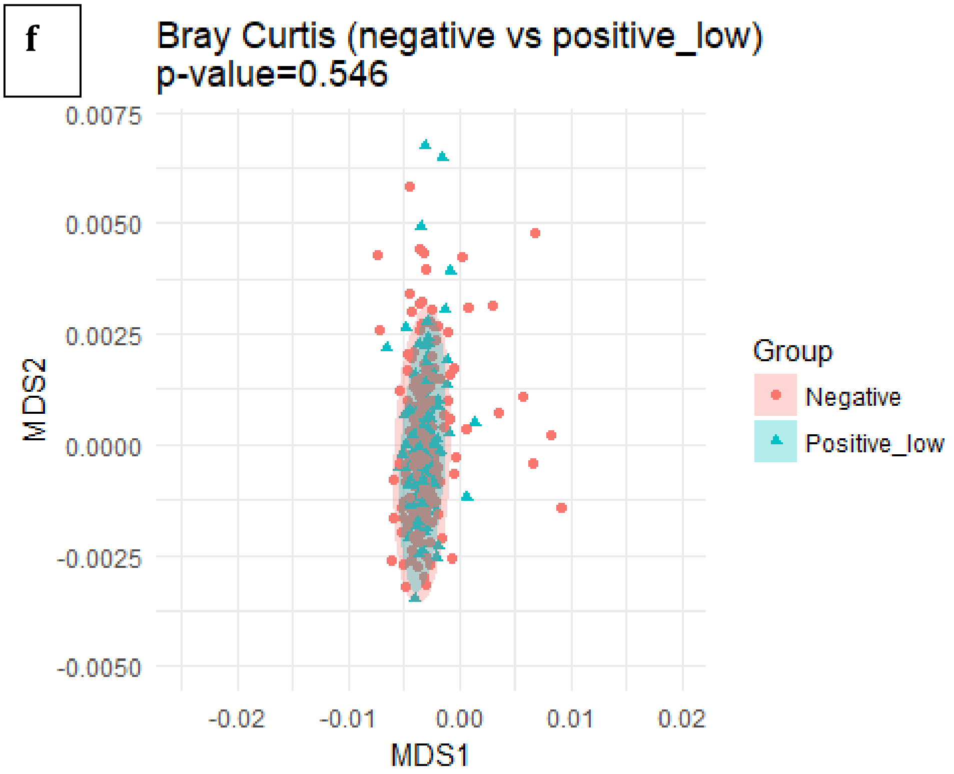
f
Bray Curtis (negative vs positive_low)
p-value=0.546
0.0075
0.0050
0.0025
0.0000
-0.0025
-0.0050
MDS2
-0.02
-0.01
0.00
0.01
0.02
MDS1
Group
Negative
Positive_low

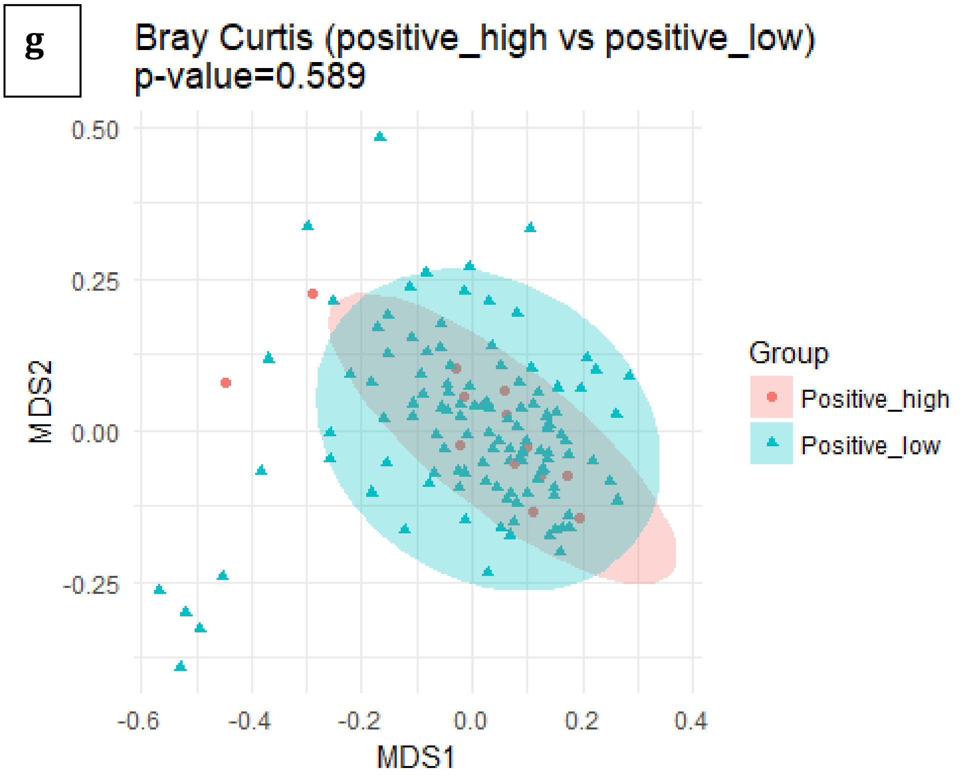
g
Bray Curtis (positive_high vs positive_low)
p-value=0.589
0.50
0.25
0.00
-0.25
MDS2
-0.6
-0.4
-0.2
0.0
0.2
0.4
MDS1
Group
Positive_high
Positive_low

Fig E. Weighted Unifrac Analysis of GAS Positive Samples Using OTU > 0.05 as Positive_high Cutoff.

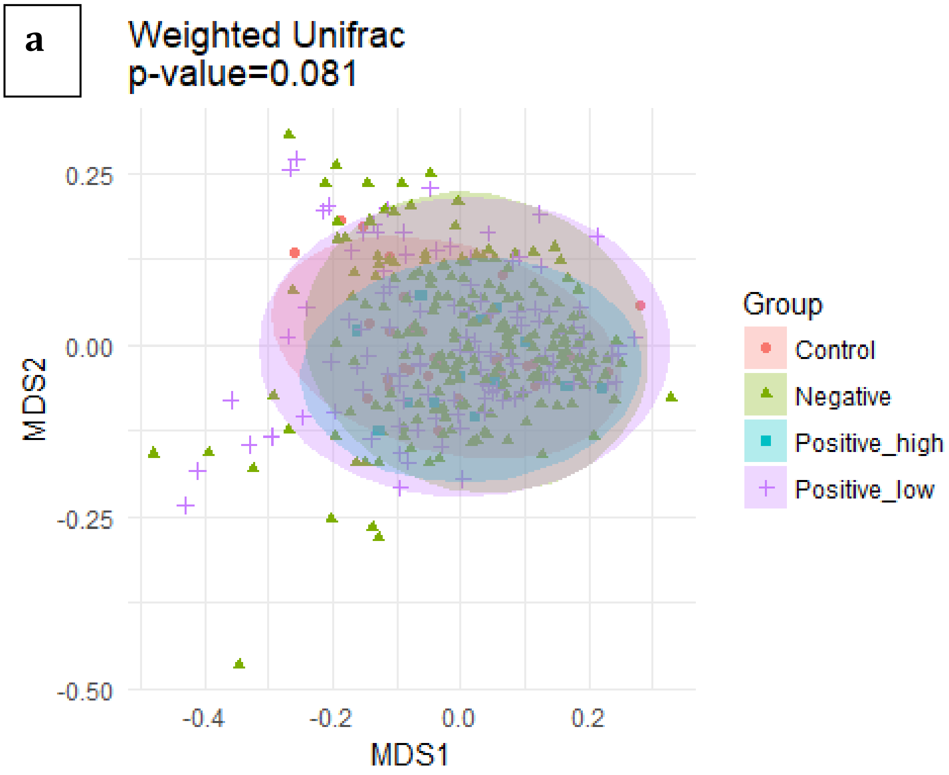

b

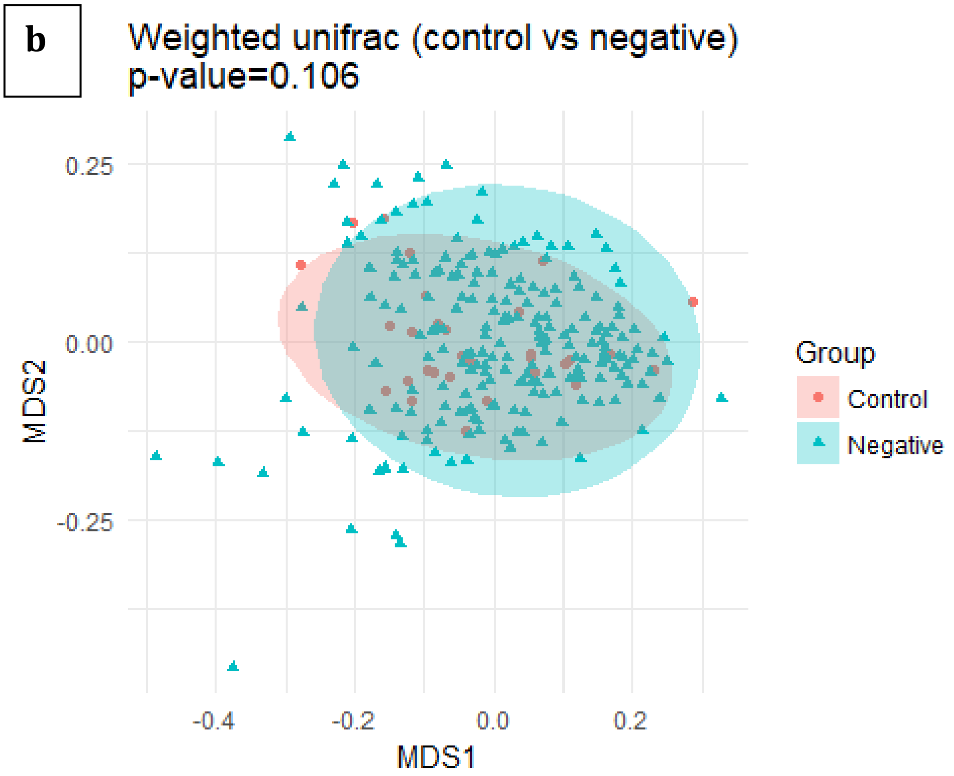
Weighted unifrac (control vs negative)
p-value=0.106
0.25
0.00
-0.25
MDS2
-0.4
-0.2
0.0
0.2
MDS1
Group
Control
Negative

c

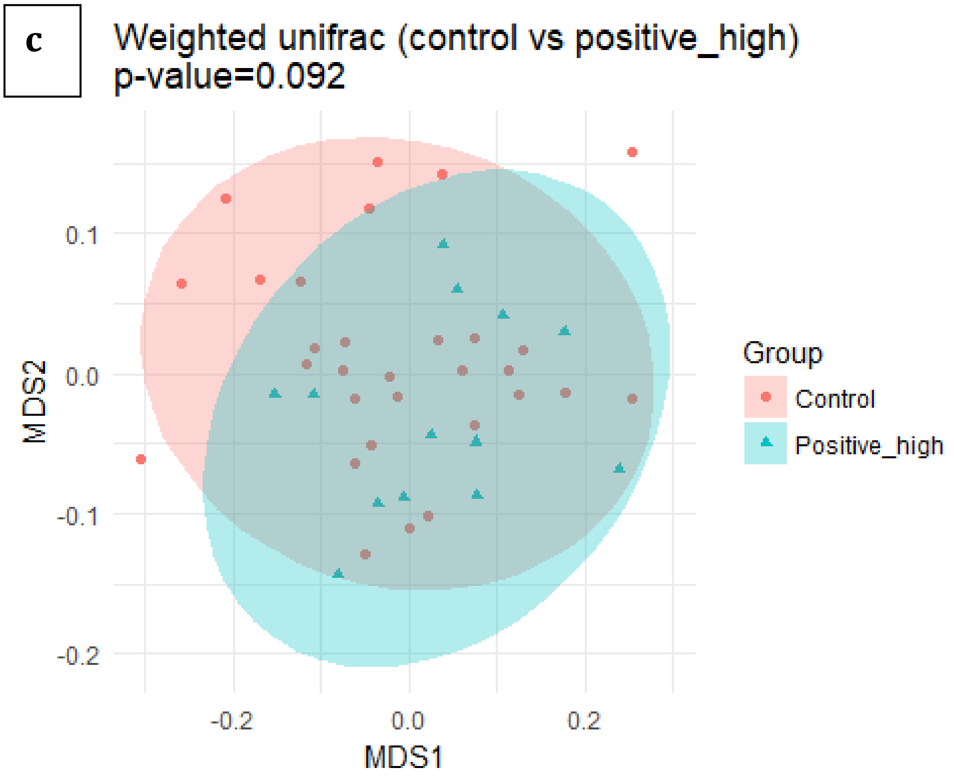
Weighted unifrac (control vs positive_high)
p-value=0.092
0.1
0.0
-0.1
-0.2
MDS2
-0.2
0.0
0.2
MDS1
Group
Control
Positive_high

d

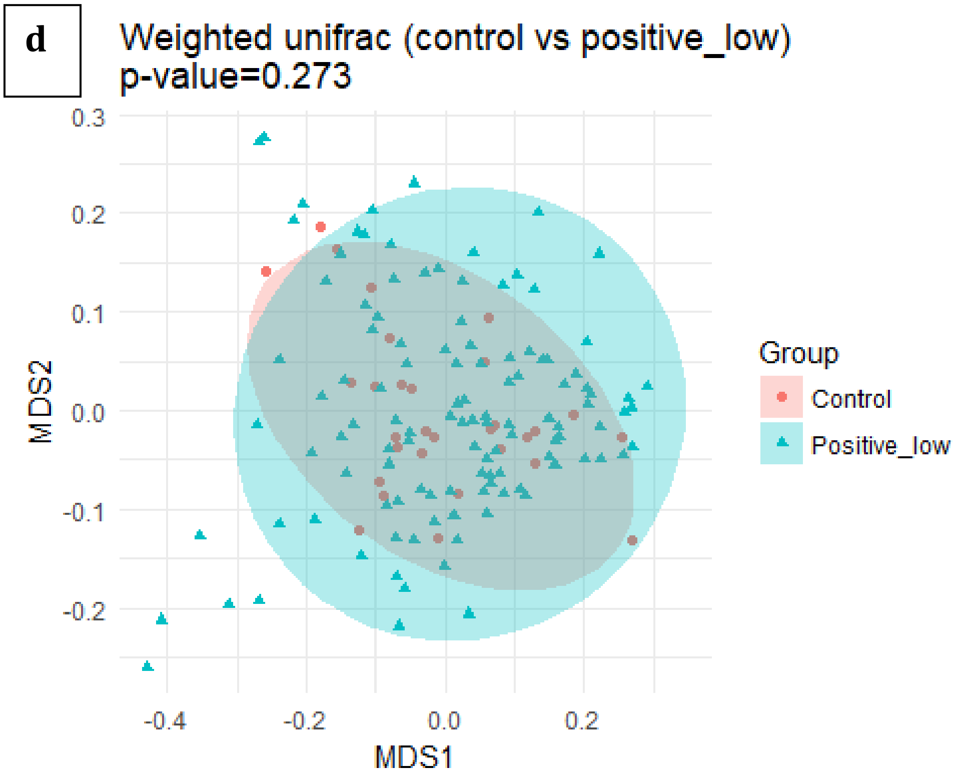
Weighted unifrac (control vs positive_low)
p-value=0.273
0.3
0.2
0.1
0.0
-0.1
-0.2
MDS2
-0.4
-0.2
0.0
0.2
MDS1
Group
Control
Positive_low

e

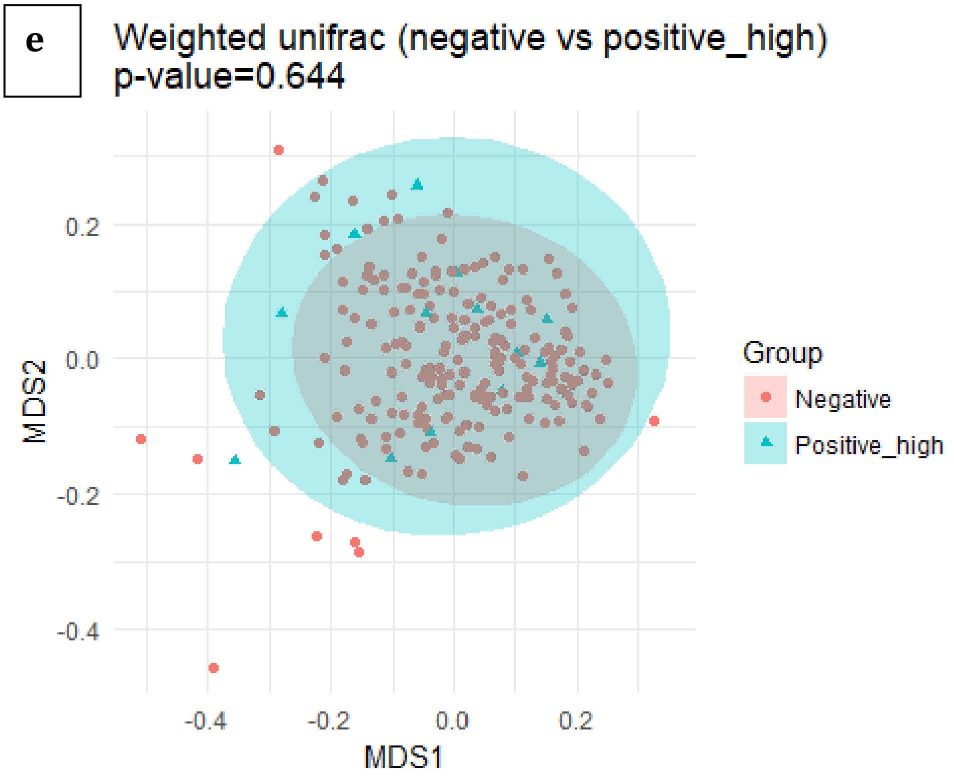
Weighted unifrac (negative vs positive_high)
p-value=0.644
0.2
0.0
-0.2
-0.4
MDS2
-0.4
-0.2
0.0
0.2
MDS1
Group
Negative
Positive_high

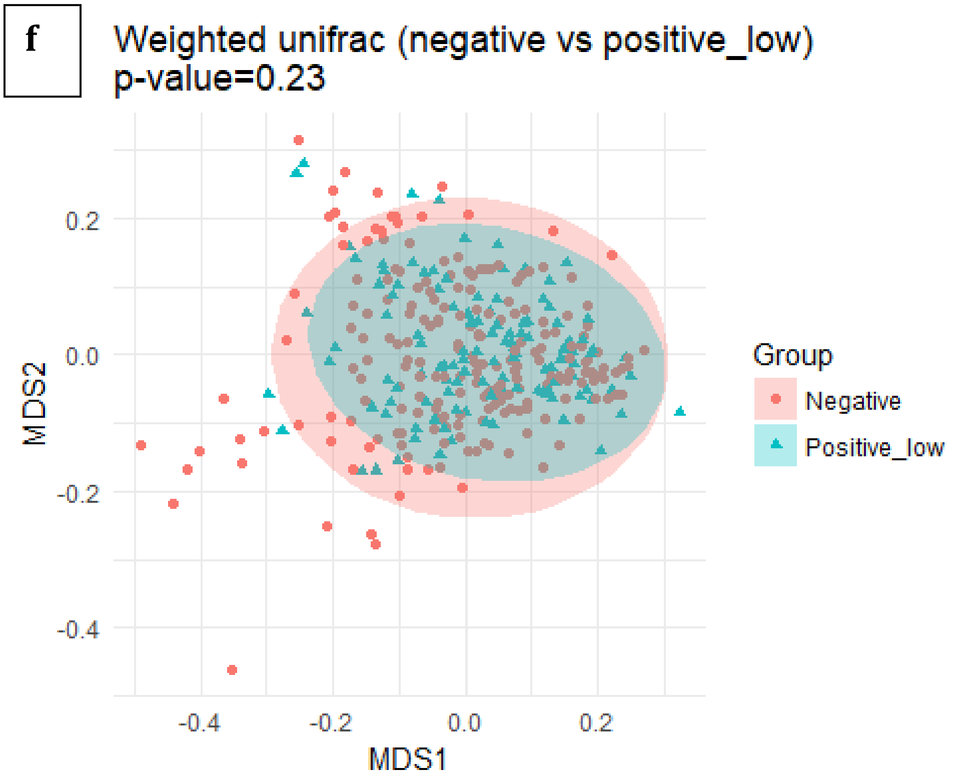
f
Weighted unifrac (negative vs positive_low)
p-value=0.23
MDS2
MDS1
0.2
0.0
-0.2
-0.4
-0.4
-0.2
0.0
0.2
Group
Negative
Positive_low

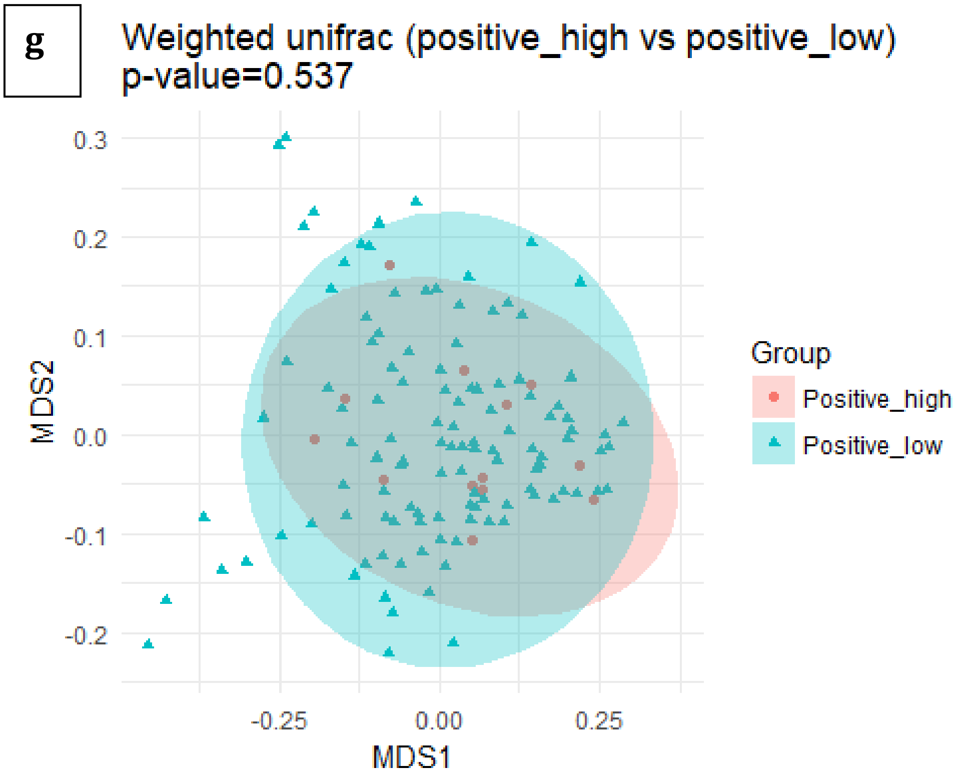
g
Weighted unifrac (positive_high vs positive_low)
p-value=0.537
MDS2
MDS1
0.3
0.2
0.1
0.0
-0.1
-0.2
-0.25
0.00
0.25
Group
Positive_high
Positive_low

Fig F. Unweighted Unifrac Analysis of GAS Positive Samples Using OTU > 0.05 as Positive_high Cutoff.

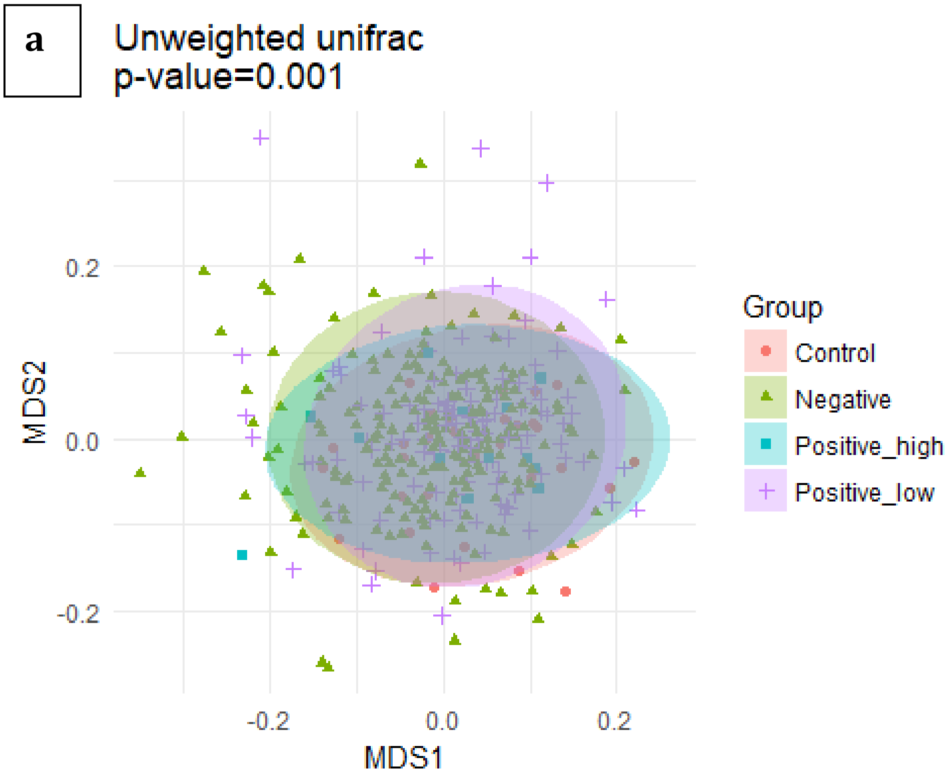

b

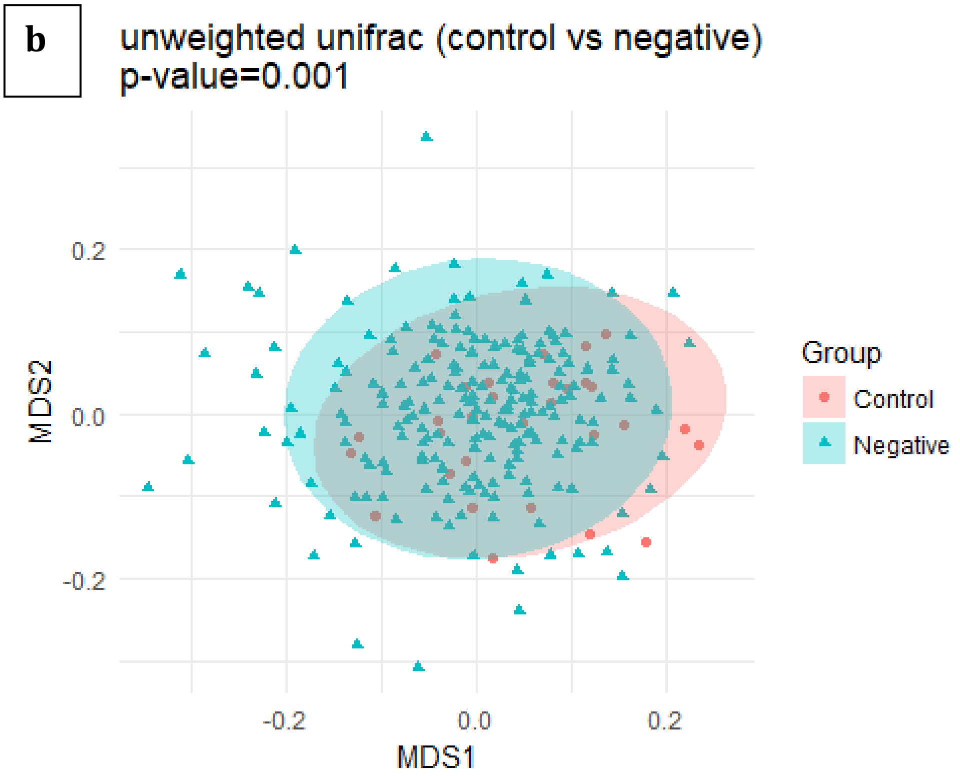
unweighted unifrac (control vs negative)
p-value=0.001
MDS2
0.2
0.0
-0.2
-0.2
0.0
0.2
MDS1
Group
Control
Negative

c

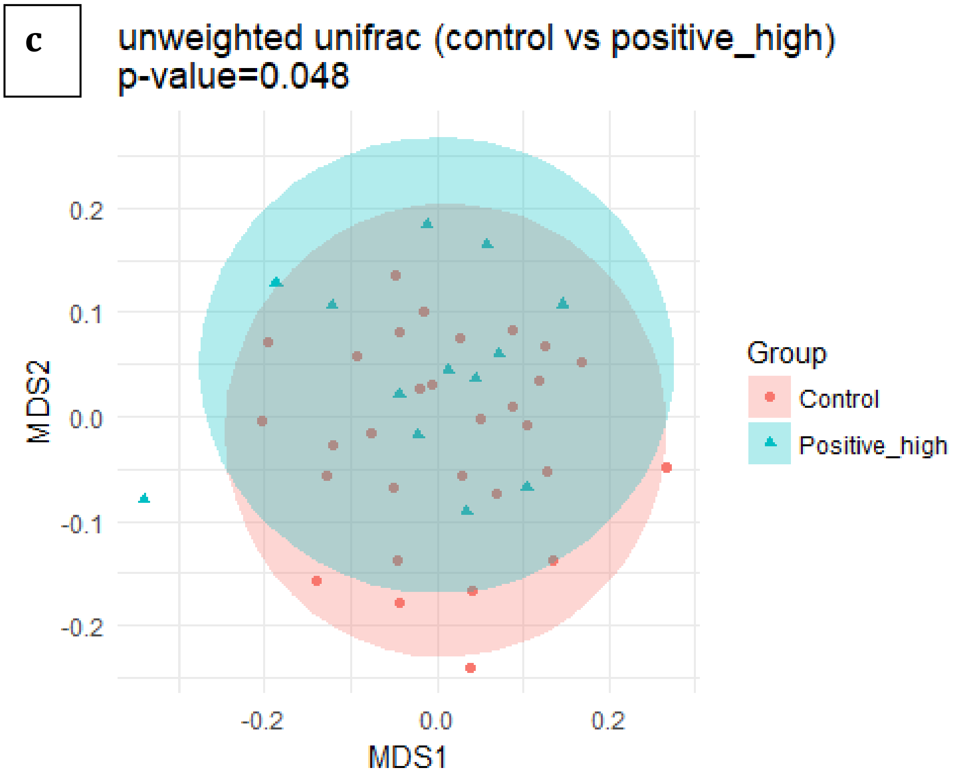
unweighted unifrac (control vs positive_high)
p-value=0.048
MDS2
0.2
0.1
0.0
-0.1
-0.2
-0.2
0.0
0.2
MDS1
Group
Control
Positive_high

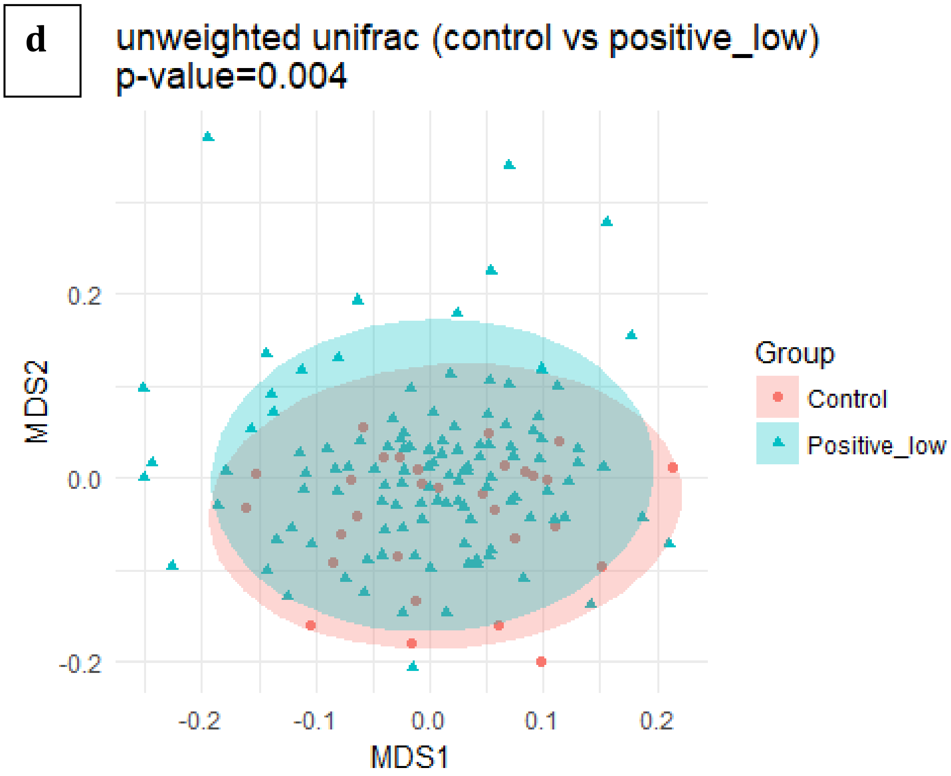
d
unweighted unifrac (control vs positive_low)
p-value=0.004
MDS2
MDS1
0.2
0.0
-0.2
-0.2
-0.1
0.0
0.1
0.2
Group
Control
Positive_low

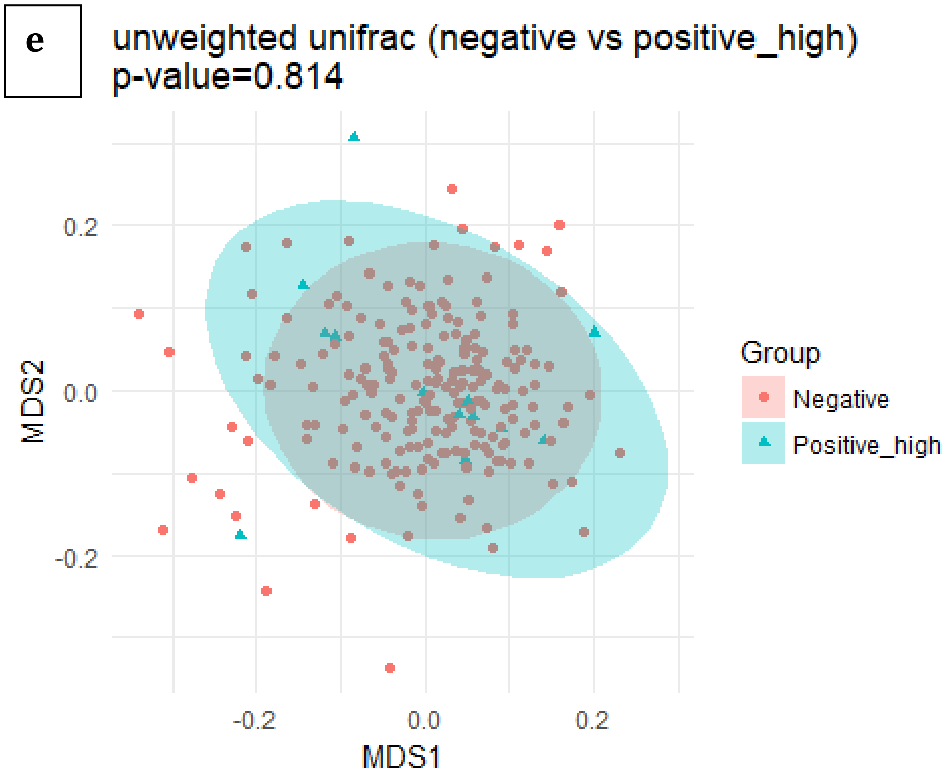
e
unweighted unifrac (negative vs positive_high)
p-value=0.814
MDS2
MDS1
0.2
0.0
-0.2
-0.2
0.0
0.2
Group
Negative
Positive_high

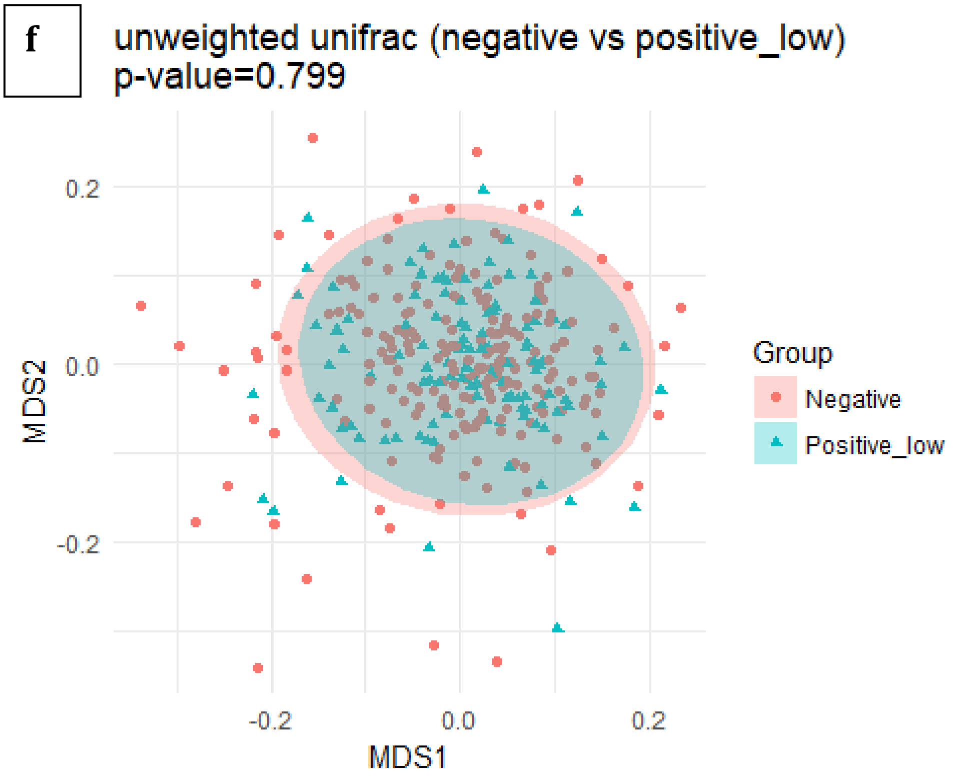
f
unweighted unifrac (negative vs positive_low)
p-value=0.799
0.2
0.0
-0.2
MDS2
-0.2
0.0
0.2
MDS1
Group
Negative
Positive_low

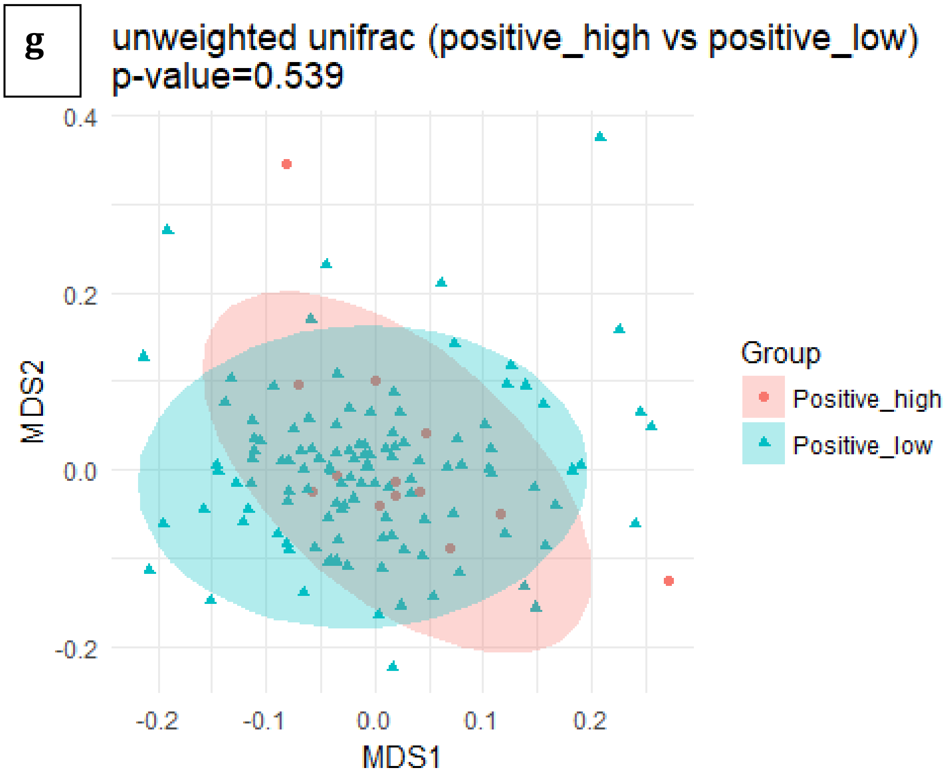
g
unweighted unifrac (positive_high vs positive_low)
p-value=0.539
0.4
0.2
0.0
-0.2
MDS2
-0.2
-0.1
0.0
0.1
0.2
MDS1
Group
Positive_high
Positive_low

Table S. P values of overall PERMANOVA.

	p-value
Bray Curtis	0.001
Weighted unifrac	0.081
Unweighted unifrac	0.001

Table T. P Values of Pair-Wise PERMANOVA.

	p-value
Bray Curtis	
control vs negative	0.008
control vs positive_high	0.001
control vs positive_low	0.026
negative vs positive_high	0.609
negative vs positive_low	0.546
positive_high vs positive_low	0.589
Weighted unifrac	
control vs negative	0.106
control vs positive_high	0.092
control vs positive_low	0.273
negative vs positive_high	0.644
negative vs positive_low	0.230
positive_high vs positive_low	0.537
Unweighted unifrac	

control vs negative	0.001
control vs positive_high	0.048
control vs positive_low	0.004
negative vs positive_high	0.814
negative vs positive_low	0.799
positive_high vs positive_low	0.539

III. GAS *(Streptococcus pyogenes):*

Analysis of GAS Positive Samples Using Positive_high Cutoff for Samples with OTU values > 0.1

Total samples 367

control: 30

positive-high: 9

positive-low: 120

negative: 208

Table U. Alpha Diversity.

Global Comparison

	Stratified by Group				p
	Control	Negative	Positive high	Positive low	
n	30	208	9	120	
chao1 (mean (sd))	260.24 (41.29)	252.44 (40.64)	251.60 (33.91)	254.49 (45.72)	0.81
observed_species (mean (sd))	210.60 (30.04)	205.72 (34.32)	196.67 (31.50)	208.03 (39.11)	0.705
PD_whole_tree (mean (sd))	14.18 (1.43)	13.37 (1.73)	13.92 (1.63)	13.78 (1.93)	0.044
shannon (mean (sd))	4.52 (0.49)	4.30 (0.60)	4.12 (0.64)	4.31 (0.70)	0.231
simpson (mean (sd))	0.91 (0.05)	0.89 (0.09)	0.85 (0.09)	0.88 (0.11)	0.346

Table V. Check Assumptions: Homogeneity of Variance (Levene's Test).
P values of Homogeneity of Variance

	p value
chao1	0.63456
observed species	0.45718
PD whole tree	0.38723
shannon	0.56577
simpson	0.41329

For chao1, observed species, pd whole tree, Shannon and Simpson-- Variances equal

Table W. P Values of ANOVA.

	p value
chao1	0.80951
observed species	0.70530
PD whole tree	0.04393
shannon	0.23149
simpson	0.34618

Table X. Chao1 (comparison in Tukey HSD).

Tukey multiple comparisons of means (95% family-wise confidence level)				
	diff	lwr	upr	adj-p
Negative vs Control	-7.7992	-29.1171	13.5187	0.7810
Positive_high vs Control	-8.6339	-50.1197	32.8518	0.9499
Positive_low vs Control	-5.7471	-28.0285	16.5343	0.9099
Positive_high vs Negative	-0.8347	-37.9990	36.3295	0.9999
Positive_low vs Negative	2.0521	-10.4610	14.5651	0.9745
Positive_low vs Positive_high	2.8868	-34.8384	40.6120	0.9973

Table Y. Observed Species (comparison in Tukey HSD).

Tukey multiple comparisons of means (95% family-wise confidence level)				
	diff	lwr	upr	adj-p
Negative vs Control	-4.8837	-22.8253	13.0580	0.8960
Positive_high vs Control	-13.9333	-48.8487	20.9820	0.7320
Positive_low vs Control	-2.5667	-21.3192	16.1859	0.9849
Positive_high vs Negative	-9.0497	-40.3280	22.2286	0.8780
Positive_low vs Negative	2.3170	-8.2143	12.8483	0.9416
Positive_low vs Positive_high	11.3667	-20.3837	43.1170	0.7920

Table Z. PD Whole Tree (comparison in Tukey HSD).

Tukey multiple comparisons of means (95% family-wise confidence level)				
	diff	lwr	upr	adj-p
Negative vs Control	-0.8064	-1.6996	0.0868	0.0932
Positive_high vs Control	-0.2570	-1.9953	1.4812	0.9811
Positive_low vs Control	-0.3959	-1.3295	0.5377	0.6931
Positive_high vs Negative	0.5493	-1.0078	2.1065	0.7993
Positive_low vs Negative	0.4104	-0.1139	0.9347	0.1823
Positive_low vs Positive_high	-0.1389	-1.7196	1.4418	0.9959

Table AA. Shannon (Pairwise comparisons with the Bonferroni correction).

Tukey multiple comparisons of means (95% family-wise confidence level)				
	diff	lwr	upr	adj-p
Negative vs Control	-0.2247	-0.5415	0.0921	0.2607
Positive_high vs Control	-0.4049	-1.0215	0.2116	0.3277
Positive_low vs Control	-0.2101	-0.5412	0.1210	0.3589
Positive_high vs Negative	-0.1802	-0.7326	0.3721	0.8342
Positive_low vs Negative	0.0146	-0.1714	0.2006	0.9971
Positive_low vs Positive_high	0.1948	-0.3658	0.7555	0.8065

Table BB. Simpson (Pairwise comparisons with the Bonferroni correction).

Tukey multiple comparisons of means (95% family-wise confidence level)				
	diff	lwr	upr	adj-p
Negative vs Control	-0.0207	-0.0673	0.0259	0.6614
Positive_high vs Control	-0.0533	-0.1439	0.0374	0.4283
Positive_low vs Control	-0.0283	-0.0769	0.0204	0.4397
Positive_high vs Negative	-0.0326	-0.1138	0.0486	0.7283
Positive_low vs Negative	-0.0076	-0.0349	0.0198	0.8910
Positive_low vs Positive_high	0.0250	-0.0574	0.1074	0.8620

Beta Diversity Analysis of GAS Positive Samples Using OTU Values > 0.1 as GAS Positive_High

Fig G. Bray Curtis Analysis of GAS Positive Samples Using OTU > 0.1 as Positive_high Cutoff.

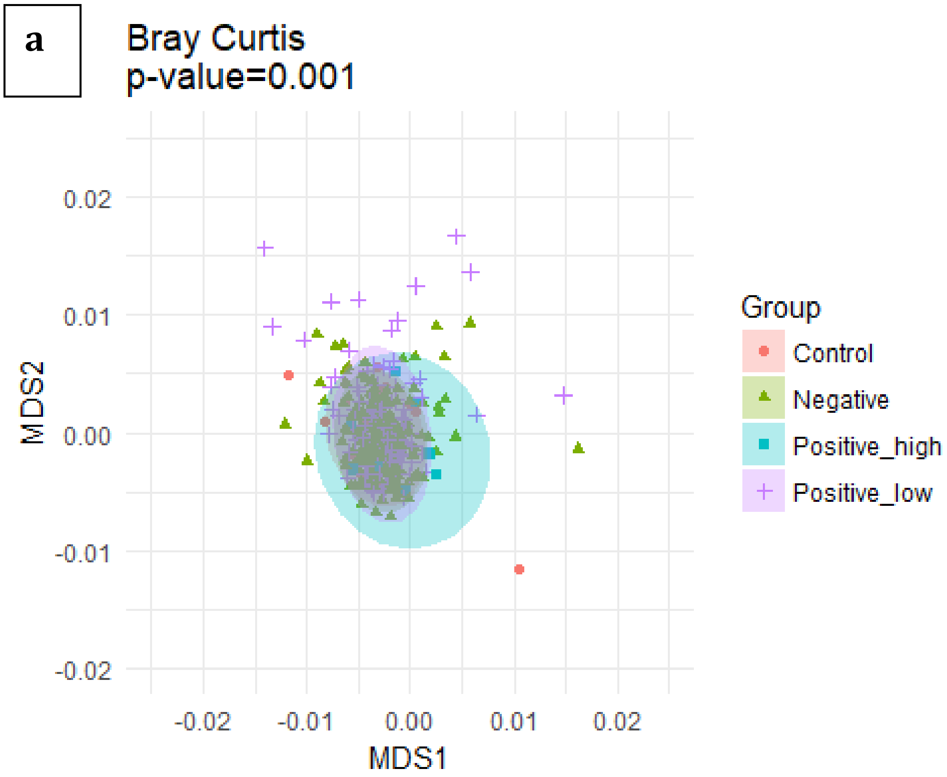

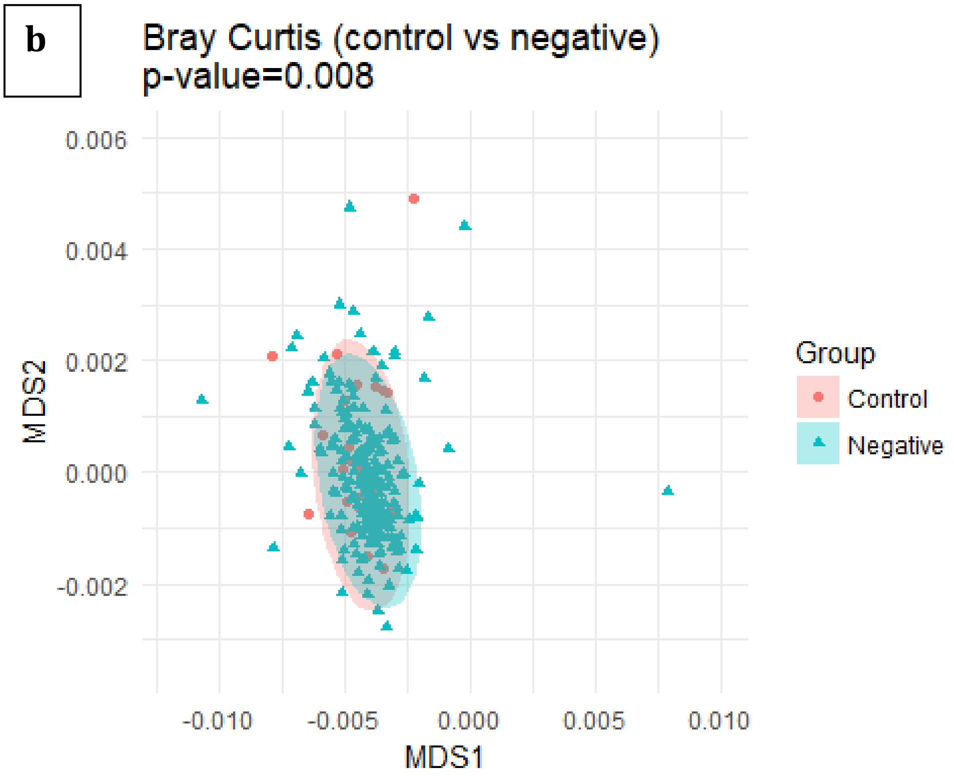
b
Bray Curtis (control vs negative)
p-value=0.008
MDS2
0.006
0.004
0.002
0.000
-0.002
-0.010
-0.005
0.000
0.005
0.010
MDS1
Group
Control
Negative

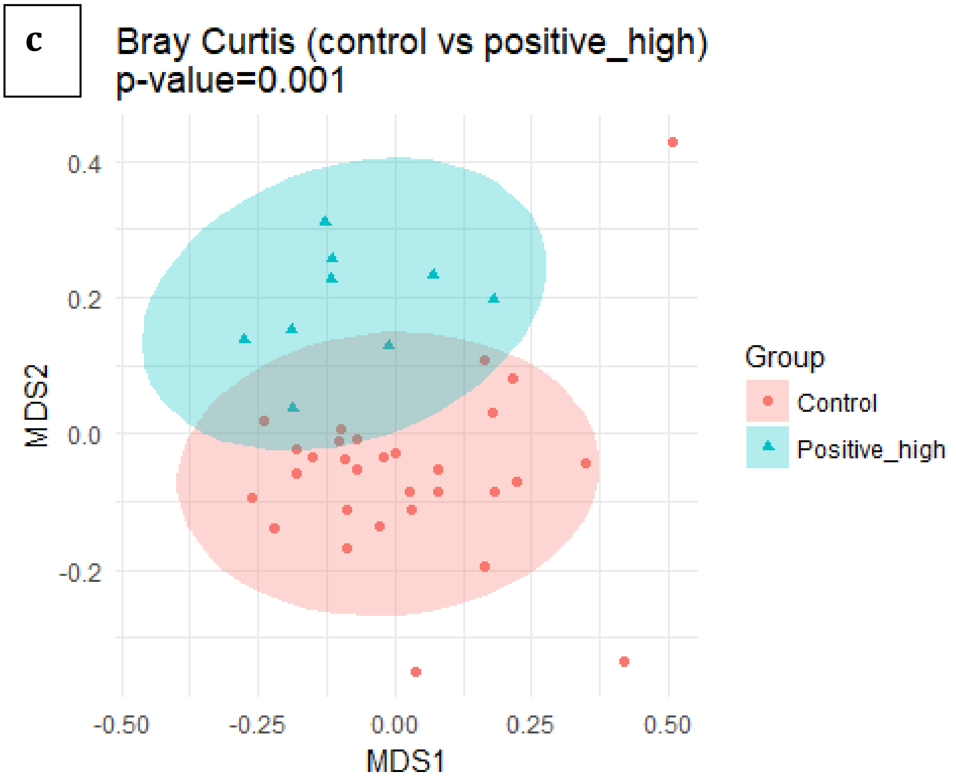
c
Bray Curtis (control vs positive_high)
p-value=0.001
MDS2
0.4
0.2
0.0
-0.2
-0.50
-0.25
0.00
0.25
0.50
MDS1
Group
Control
Positive_high

d

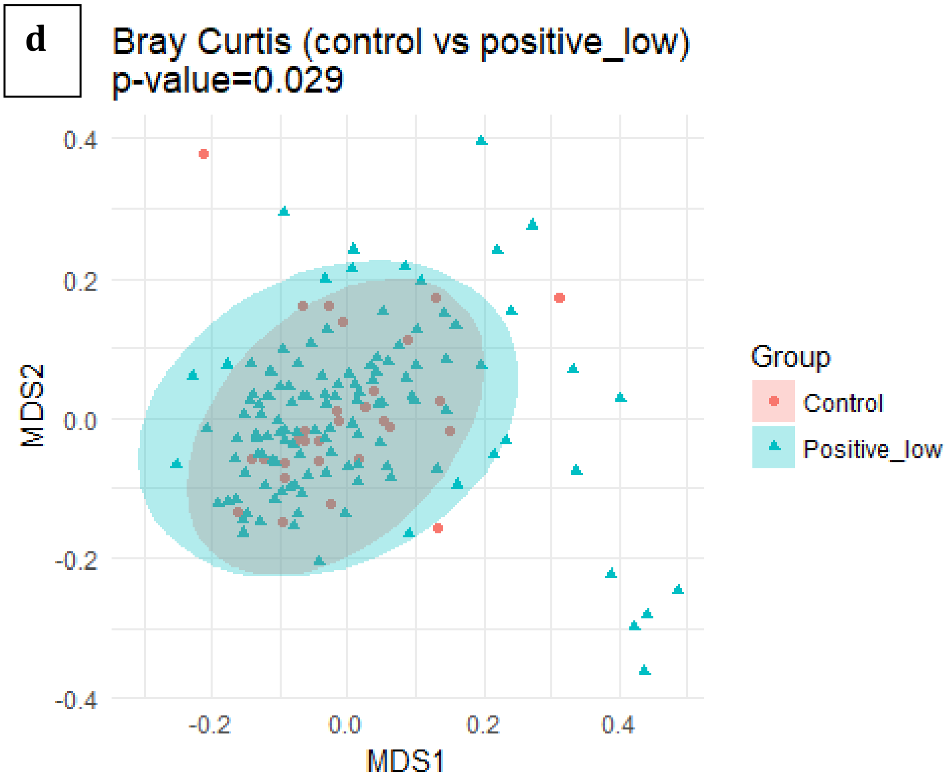
Bray Curtis (control vs positive_low)
p-value=0.029
MDS2
0.4
0.2
0.0
-0.2
-0.4
-0.2
0.0
0.2
0.4
MDS1
Group
Control
Positive_low

e

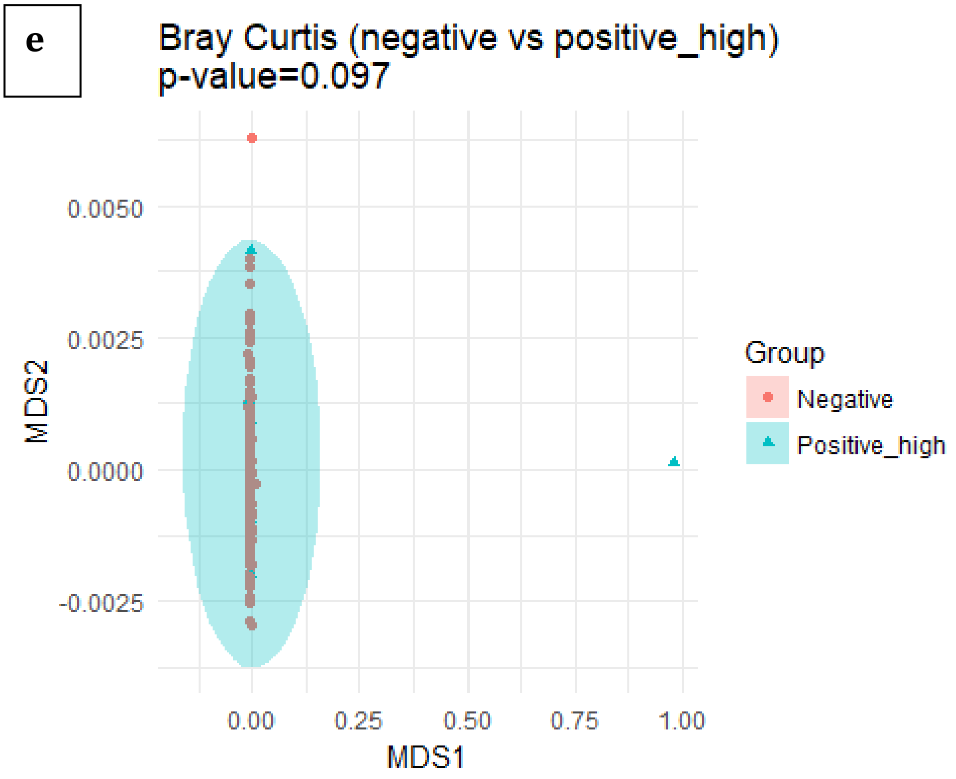
Bray Curtis (negative vs positive_high)
p-value=0.097
MDS2
0.0050
0.0025
0.0000
-0.0025
0.00
0.25
0.50
0.75
1.00
MDS1
Group
Negative
Positive_high

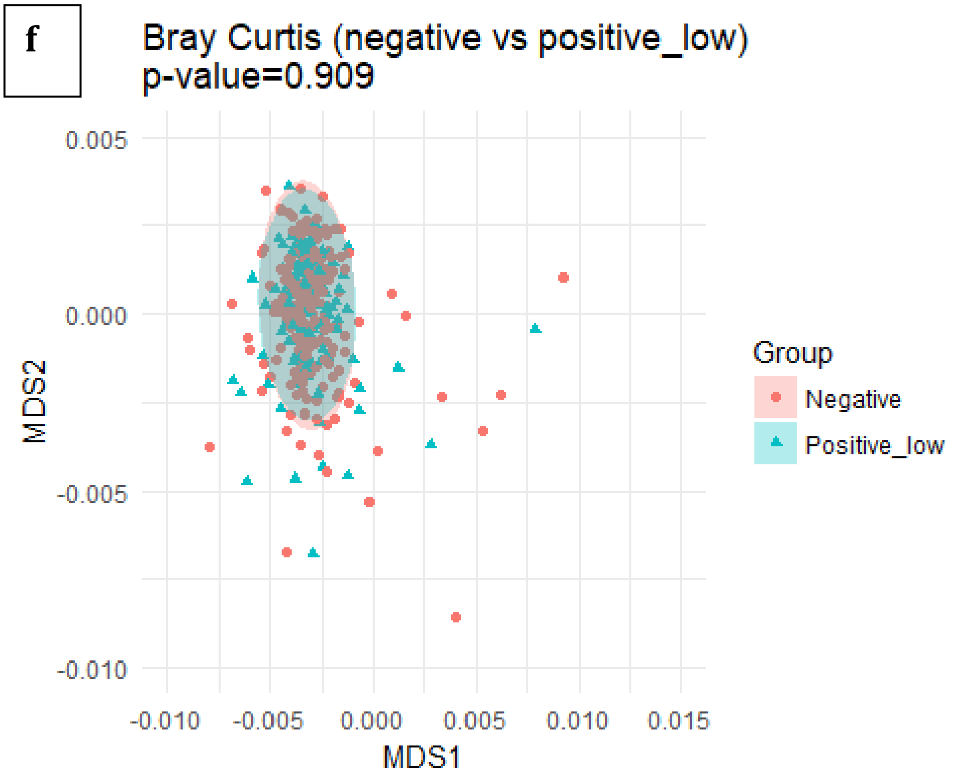
f
Bray Curtis (negative vs positive_low)
p-value=0.909
0.005
0.000
-0.005
-0.010
MDS2
-0.010
-0.005
0.000
0.005
0.010
0.015
MDS1
Group
Negative
Positive_low

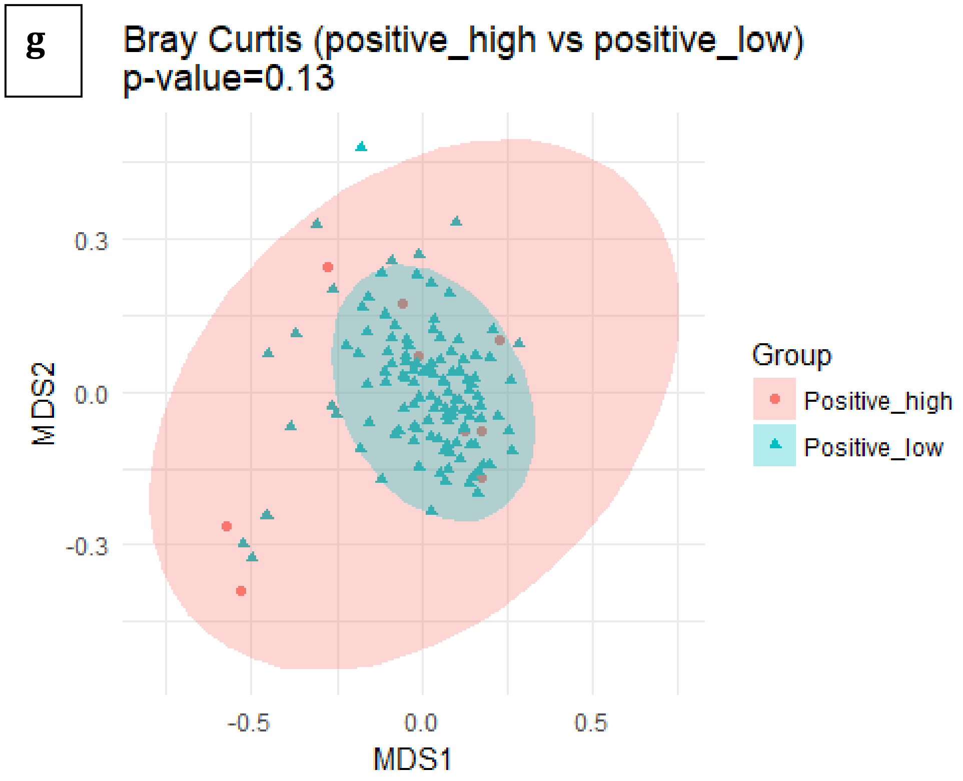
g
Bray Curtis (positive_high vs positive_low)
p-value=0.13
0.3
0.0
-0.3
MDS2
-0.5
0.0
0.5
MDS1
Group
Positive_high
Positive_low

Fig H. Weighted Unifrac Analysis of GAS Positive Samples Using OTU > 0.1 as Positive_high Cutoff.

a

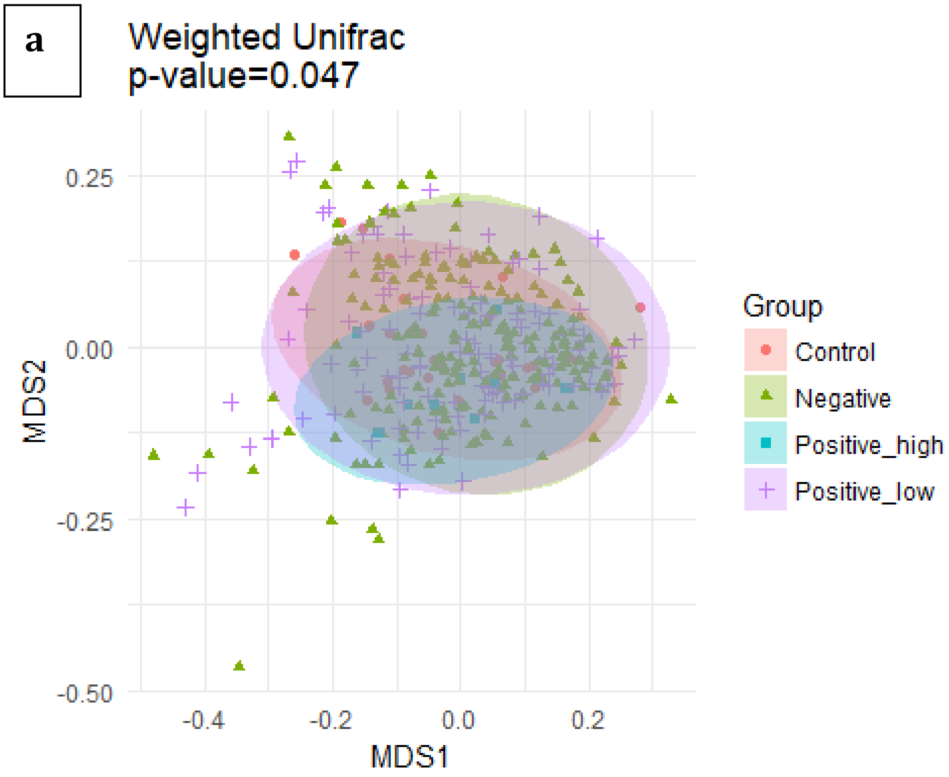

b

Weighted unifrac (control vs negative)
p-value=0.106

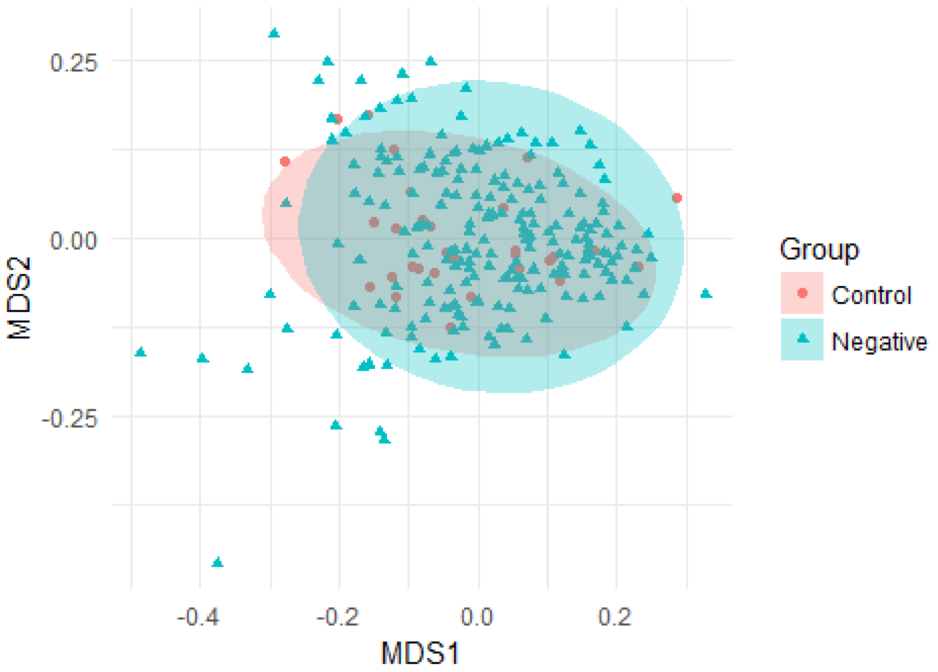

c

Weighted unifrac (control vs positive_high)
p-value=0.069

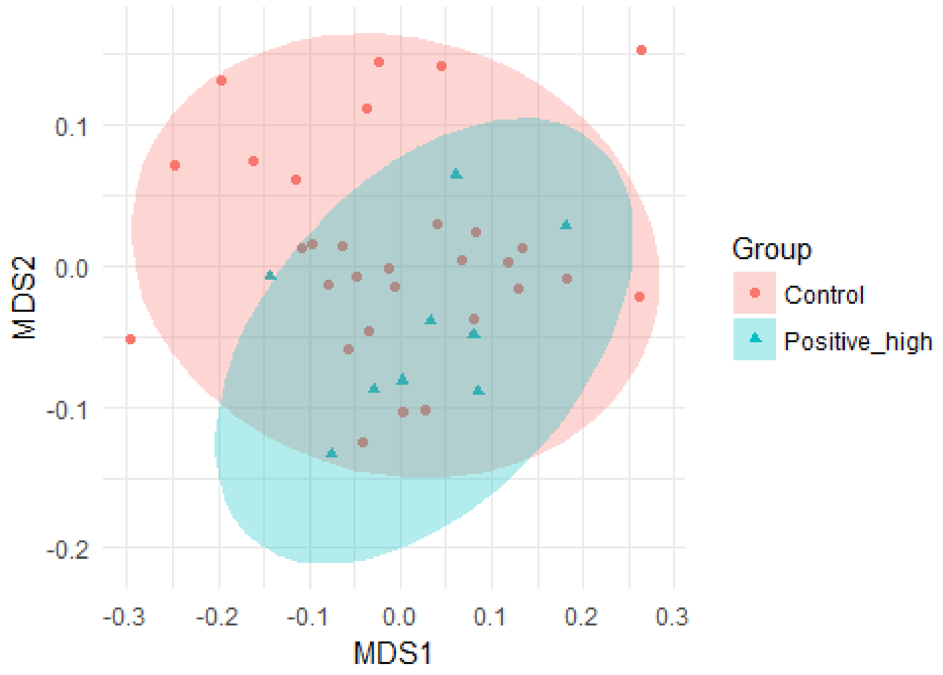

d

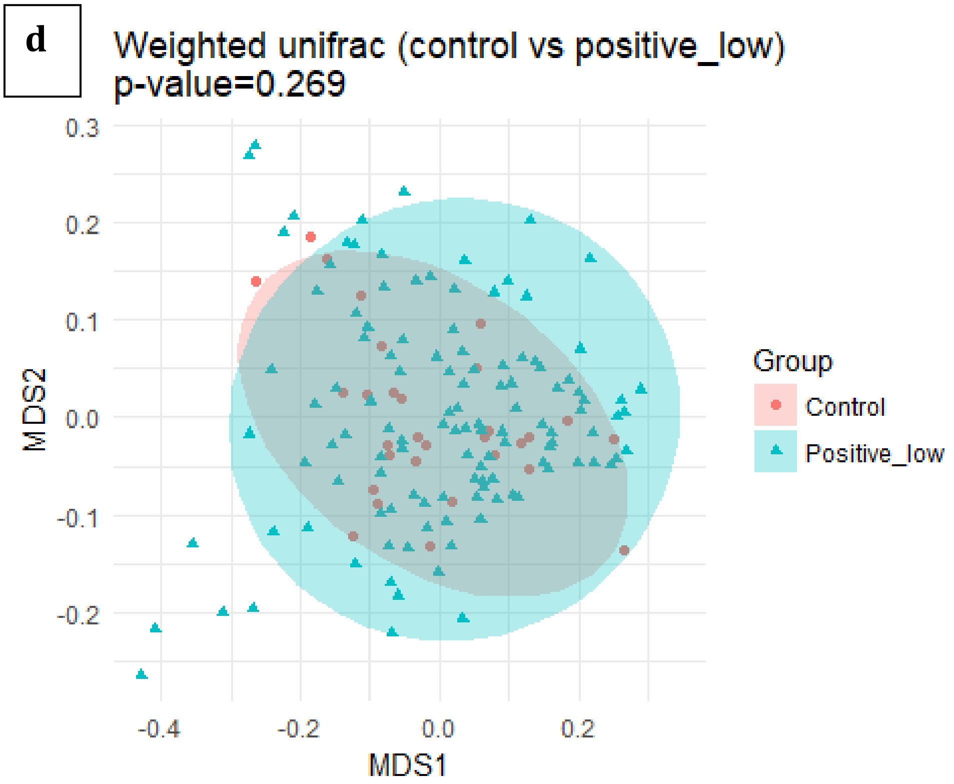
Weighted unifrac (control vs positive_low)
p-value=0.269
0.3
0.2
0.1
0.0
-0.1
-0.2
MDS2
-0.4
-0.2
0.0
0.2
MDS1
Group
Control
Positive_low

e

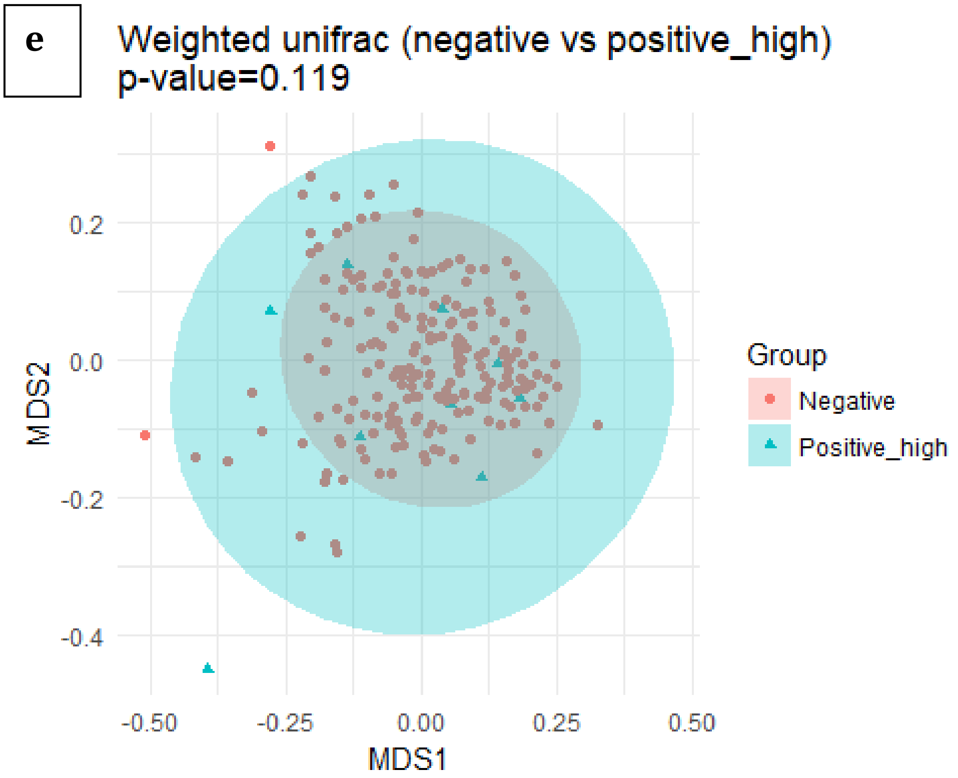
Weighted unifrac (negative vs positive_high)
p-value=0.119
0.2
0.0
-0.2
-0.4
MDS2
-0.50
-0.25
0.00
0.25
0.50
MDS1
Group
Negative
Positive_high

f

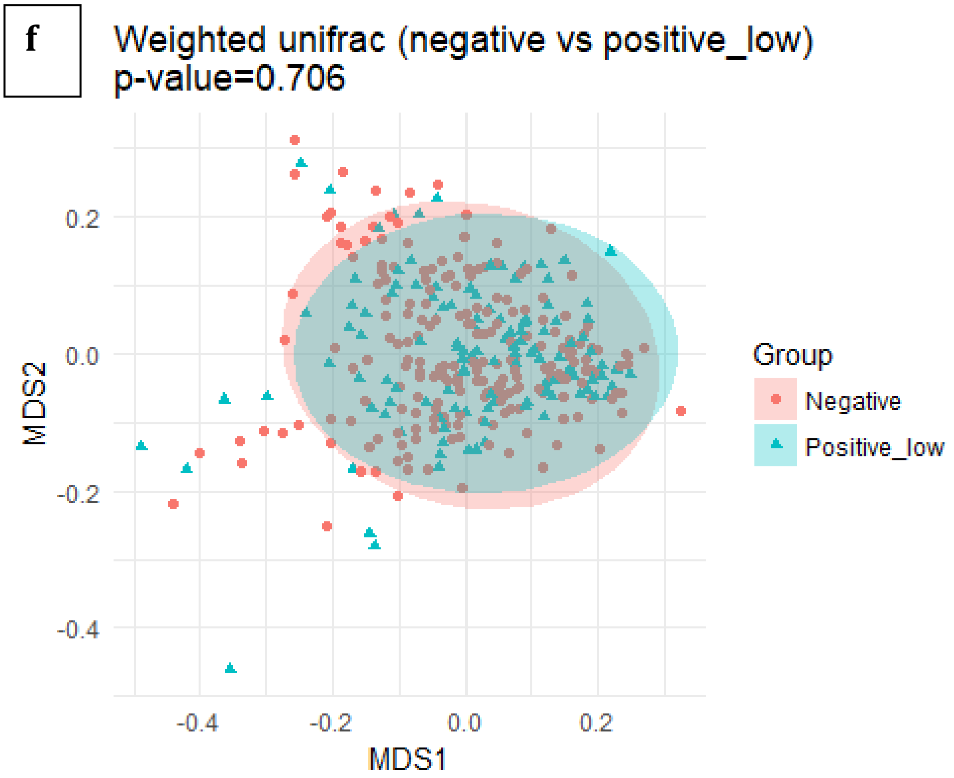
Weighted unifrac (negative vs positive_low)
p-value=0.706
0.2
0.0
-0.2
-0.4
MDS2
-0.4
-0.2
0.0
0.2
MDS1
Group
Negative
Positive_low

g

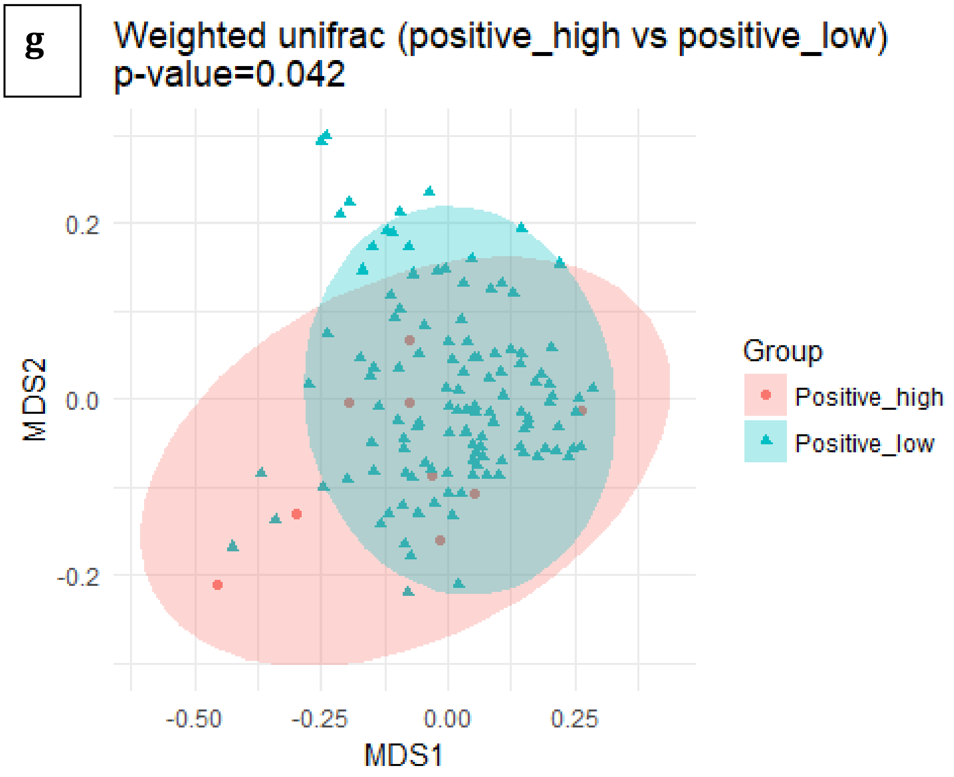
Weighted unifrac (positive_high vs positive_low)
p-value=0.042
0.2
0.0
-0.2
MDS2
-0.50
-0.25
0.00
0.25
MDS1
Group
Positive_high
Positive_low

Fig I. Unweighted Unifrac Analysis of GAS Positive Samples Using OTU > 0.1 as Positive_high Cutoff.

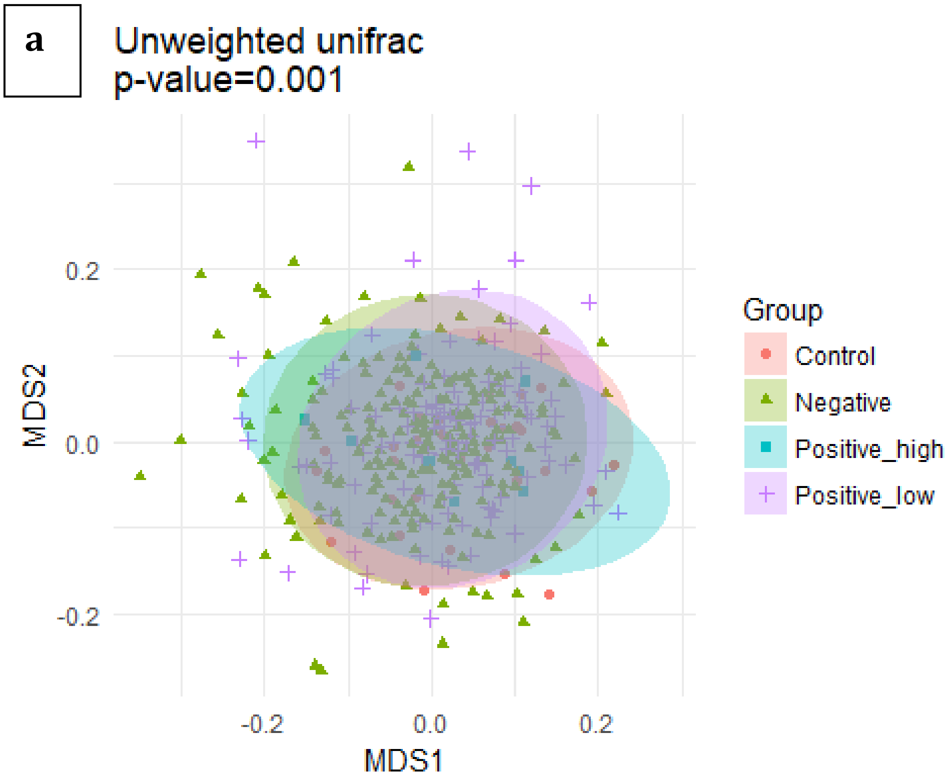

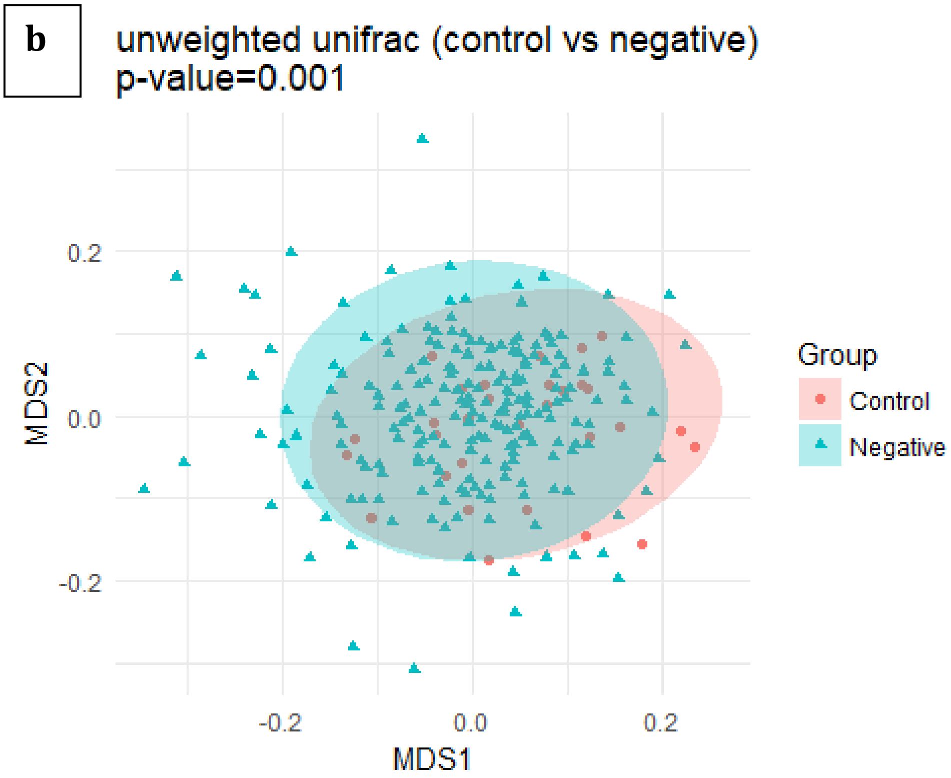
b
unweighted unifrac (control vs negative)
p-value=0.001
0.2
0.0
-0.2
MDS2
-0.2
0.0
0.2
MDS1
Group
Control
Negative

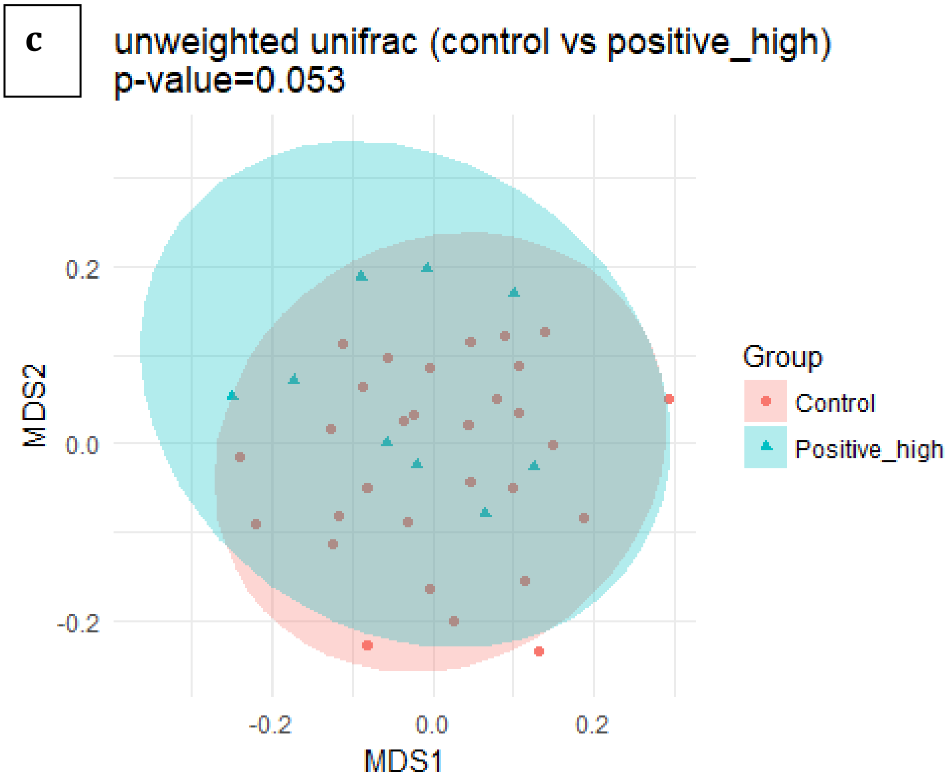
c
unweighted unifrac (control vs positive_high)
p-value=0.053
0.2
0.0
-0.2
MDS2
-0.2
0.0
0.2
MDS1
Group
Control
Positive_high

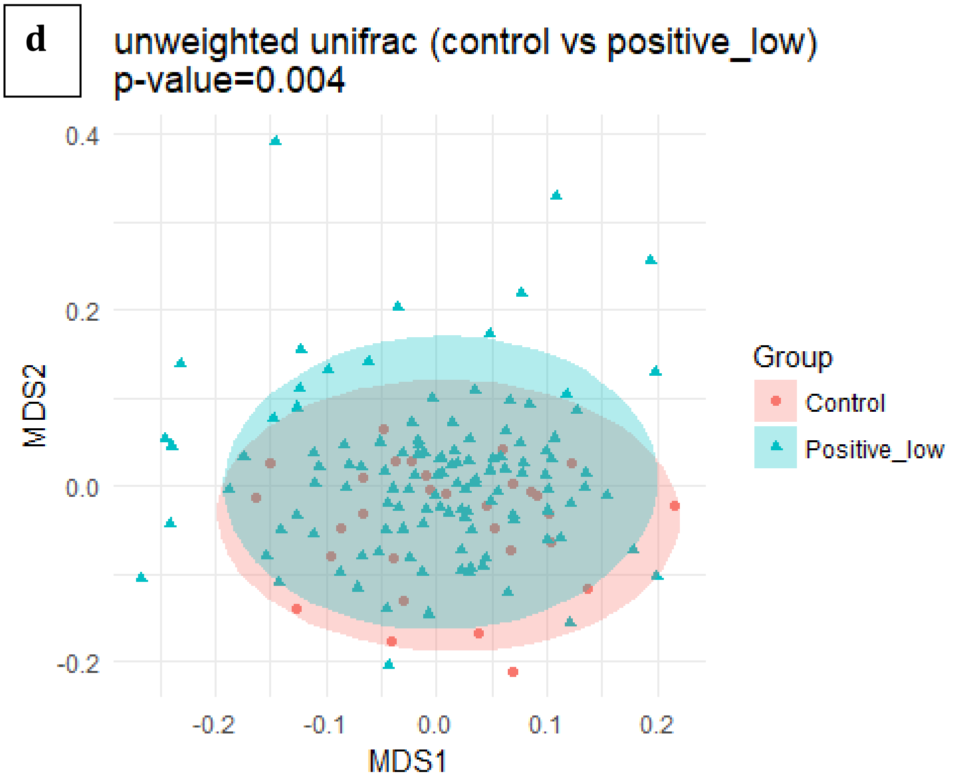
d
unweighted unifrac (control vs positive_low)
p-value=0.004
MDS2
MDS1
0.4
0.2
0.0
-0.2
-0.2
-0.1
0.0
0.1
0.2
Group
Control
Positive_low

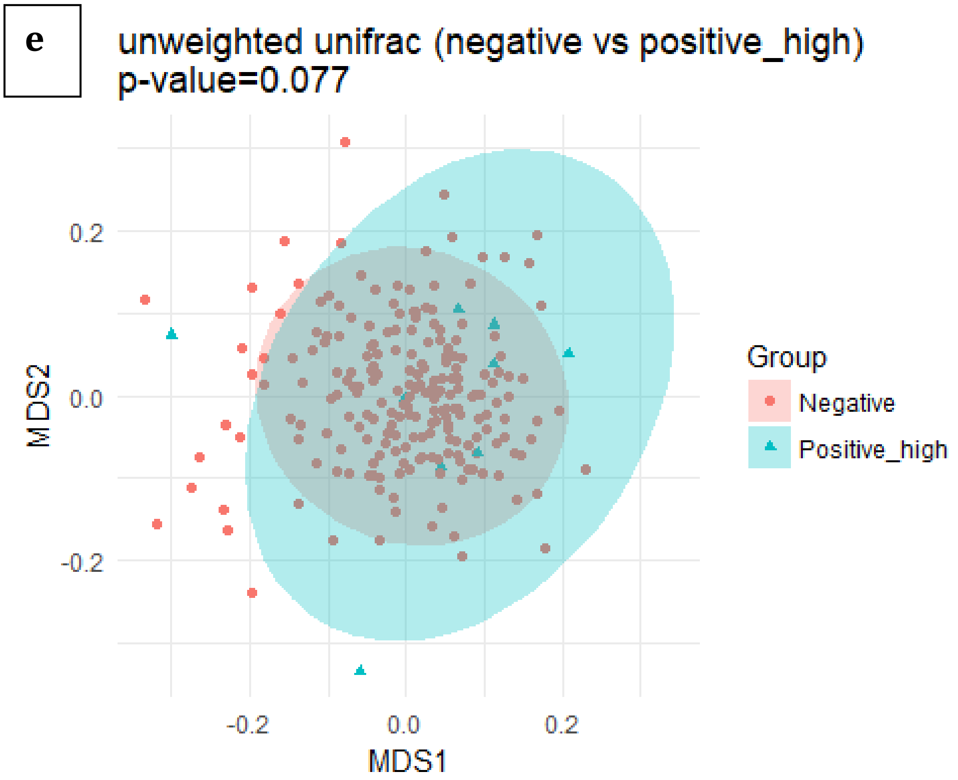
e
unweighted unifrac (negative vs positive_high)
p-value=0.077
MDS2
MDS1
0.2
0.0
-0.2
-0.2
0.0
0.2
Group
Negative
Positive_high

f

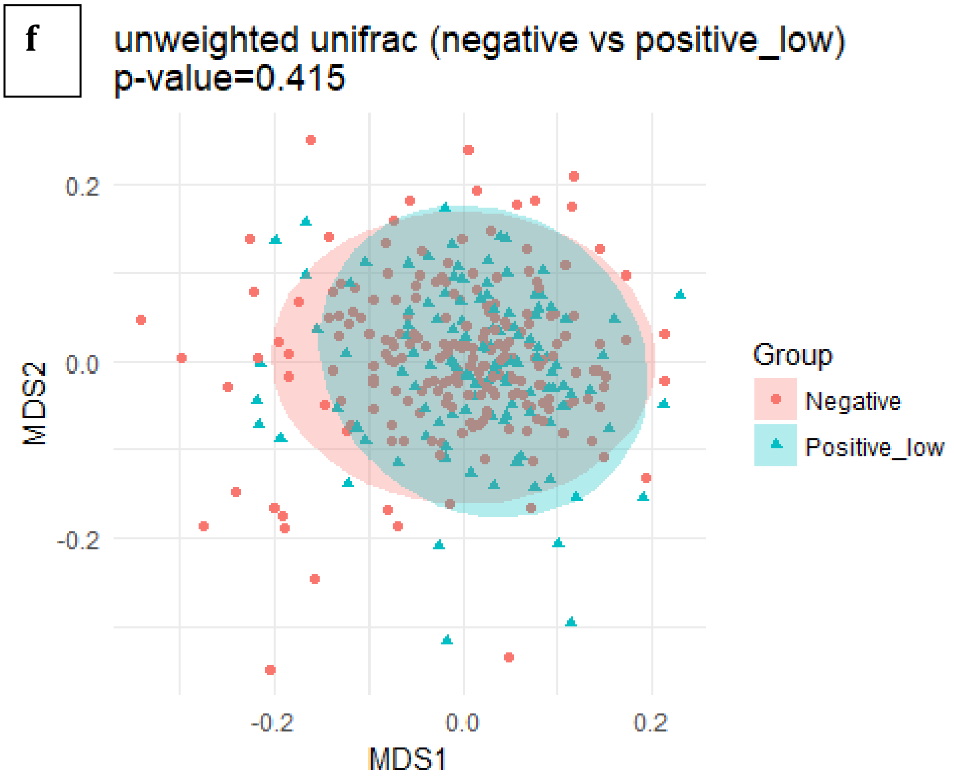
unweighted unifrac (negative vs positive_low)
p-value=0.415
MDS2
MDS1
0.2
0.0
-0.2
-0.2
0.0
0.2
Group
Negative
Positive_low

g

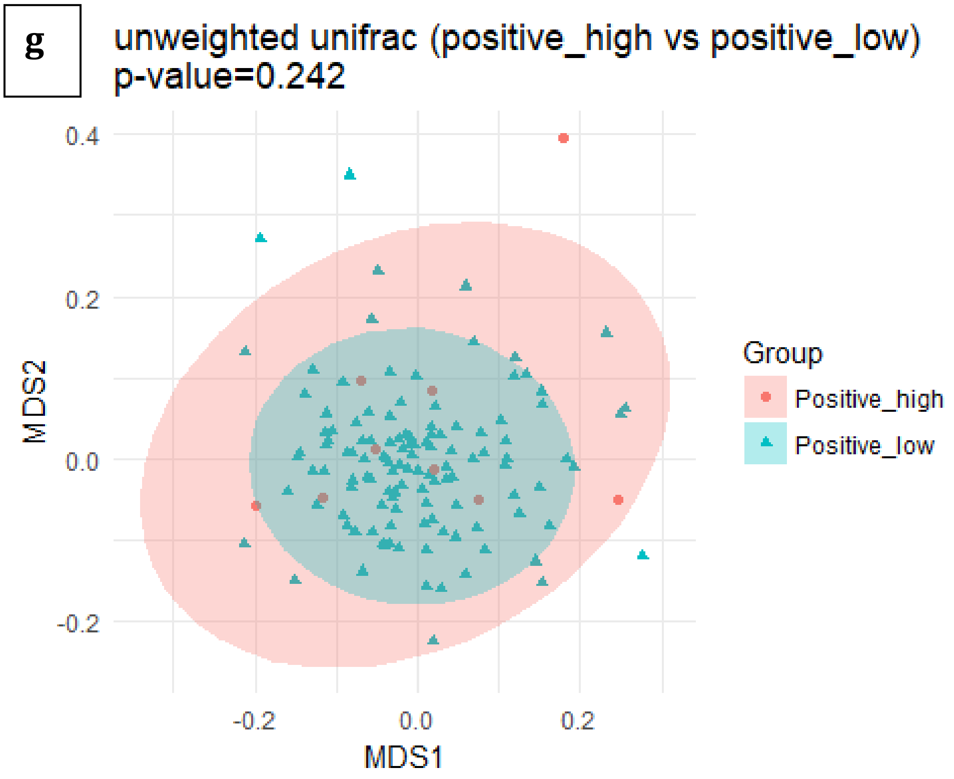
unweighted unifrac (positive_high vs positive_low)
p-value=0.242
MDS2
MDS1
0.4
0.2
0.0
-0.2
-0.2
0.0
0.2
Group
Positive_high
Positive_low

Table CC. P Values of Overall PERMANOVA.

	p-value
Bray Curtis	0.001
Weighted unifrac	0.047
Unweighted unifrac	0.001

Table DD. P Values of Pair-Wise PERMANOVA.

	p-value
Bray Curtis	
control vs negative	0.008
control vs positive_high	0.001
control vs positive_low	0.029
negative vs positive_high	0.097
negative vs positive_low	0.909
positive_high vs positive_low	0.130
Weighted unifrac	
control vs negative	0.106
control vs positive_high	0.069
control vs positive_low	0.269
negative vs positive_high	0.119
negative vs positive_low	0.706
positive_high vs positive_low	0.042
Unweighted unifrac	
control vs negative	0.001
control vs positive_high	0.053
control vs positive_low	0.004
negative vs positive_high	0.077
negative vs positive_low	0.415
positive_high vs positive_low	0.242
